# Synthesis, Optimization, and Biological Evaluation of Corrinated Conjugates of the GLP-1R Agonist Exendin-4

**DOI:** 10.1021/acs.jmedchem.1c00185

**Published:** 2021-03-06

**Authors:** Ian C. Tinsley, Tito Borner, MacKenzie L. Swanson, Oleg G. Chepurny, Sarah A. Doebley, Varun Kamat, Ian R. Sweet, George G. Holz, Matthew R. Hayes, Bart C. De Jonghe, Robert P. Doyle

**Affiliations:** † Department of Chemistry, Syracuse University, 111 College Place, Syracuse, New York 13244, United States; ‡ Department of Medicine, State University of New York, Upstate Medical University, Syracuse, New York 13210, United States; § Department of Medicine, University of Washington, Medicine Diabetes Institute, Seattle, Washington 98109, United States; ∥ Department of Psychiatry, University of Pennsylvania, Perelman School of Medicine, Philadelphia, Pennsylvania 19104, United States; ¶ Department of Biobehavioral Health Sciences, University of Pennsylvania, School of Nursing, Philadelphia, Pennsylvania 19104, United States

## Abstract

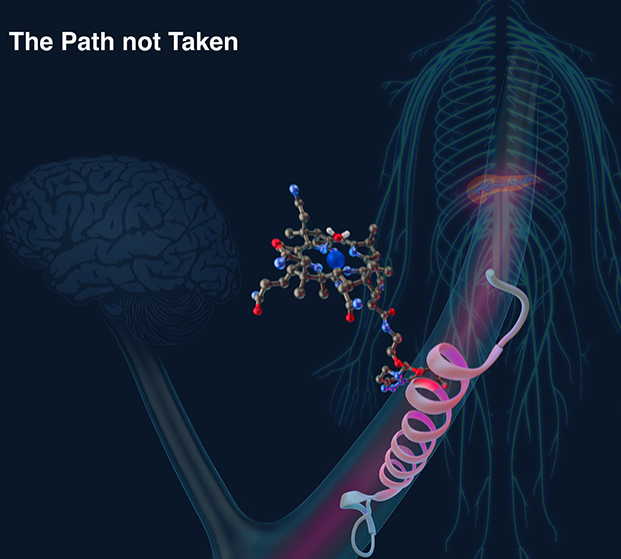

Corrination is the conjugation of a corrin ring containing molecule, such as vitamin B_12_ (B12) or B12 biosynthetic precursor dicyanocobinamide (Cbi), to small molecules, peptides, or proteins with the goal of modifying pharmacology. Recently, a corrinated GLP-1R agonist (GLP-1RA) exendin-4 (Ex4) has been shown *in vivo* to have reduced penetration into the central nervous system relative to Ex4 alone, producing a glucoregulatory GLP-1RA devoid of anorexia and emesis. The study herein was designed to optimize the lead conjugate for GLP-1R agonism and binding. Two specific conjugation sites were introduced in Ex4, while also utilizing various linkers, so that it was possible to identify Cbi conjugates of Ex4 that exhibit improved binding and agonist activity at the GLP-1R. An optimized conjugate (**22**), comparable with Ex4, was successfully screened and subsequently assayed for insulin secretion in rat islets and *in vivo* in shrews for glucoregulatory and emetic behavior, relative to Ex4.

## Introduction

**Figure 1:**
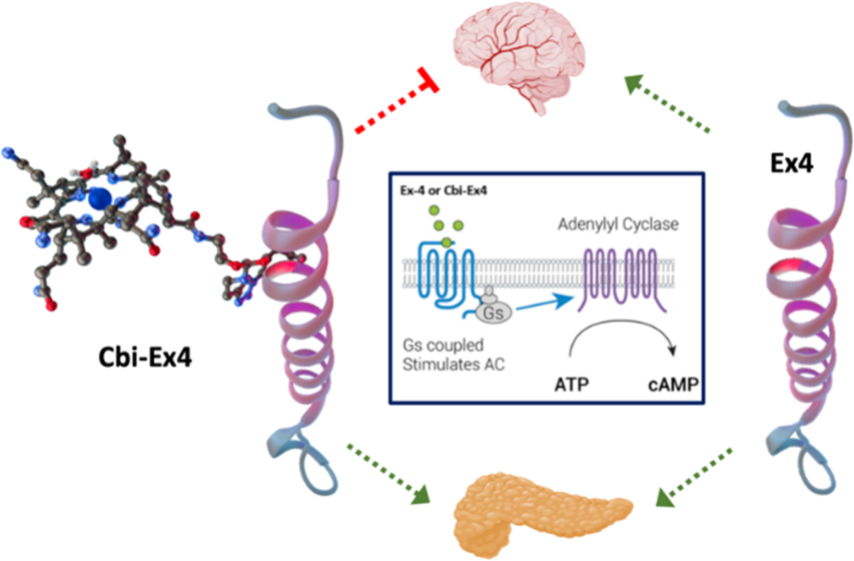
Corrination of Ex4 (Cbi-Ex4) does not prevent GLP-1R agonism in the pancreas but does mitigate agonism in the CNS as tracked by emesis and anorexia.^18^ Ex4 alone agonizes GLP-1R populations in both the pancreas and CNS.

The dramatic rise of type 2 diabetes (T2D) and obesity as comorbidities has driven a concomitant rise in new pharmaceutical interventions to treat such disorders, either together or individually.^1−7^ One extremely successful family of pharmaceuticals in this field has been that of glucagon-like peptide-1 receptor agonists (GLP-1RAs),^8−13^ such as exenatide (i.e., Exendin-4; Ex4), liraglutide,^14−17^ and semaglutide.^16−18^ Exenatide and liraglutide confer glucoregulation in tandem with a body weight reduction of 5–6% over ∼1 year,^16,17^ while semaglutide can produce a weight loss of 10–12% over the same time period.^16,17^ The hypophagic effects of all GLP-1RAs are however accompanied by nausea and vomiting (emesis) in upward of 25% of patients,^19−25^ the result of penetrance and direct action of the GLP-1RA in the central nervous system (CNS), particularly in the nucleus tractus solitarius and area postrema of the hind-brain.^26−29^ Thus, the development of a GLP-1RA that does not access the CNS would be expected then to mitigate the side effects of nausea and emesis and reduce the hypophagic effects. The reasons for removing the side effects are obvious, but less clear is the fact that there is a current unmet clinical need to broaden pharmacopoeia for certain subpopulations of T2D patients, including lean individuals, those in a state of cachexia (wasting of body tissues/chronic weight loss), or those who must avoid weight loss from other life-threatening diseases such as chronic obstructive pulmonary disease, cystic fibrosis, cancer, and HIV, among others.^30−34^ Thus, we set out to create GLP-1RAs with reduced brain penetrance but with retained, comparable pharmacodynamic profiles on pancreatic GLP-1R populations (Figure 1).^35−39^ Recently, we have taken the approach of utilizing components of the vitamin B_12_ (B12) dietary uptake pathway including that of the corrin ring containing B12^35−38,40^ itself as well as B12 precursors such as dicyanocobinamide (Cbi)^39^ in a process we have coined “corrination”. The benefits of corrination over other conjugation modes lie in the ability to specifically target select corrin binding proteins such as intrinsic factor, transcobalamin, or haptocorrin for use in targeting or to affect pharmacokinetics, modify solubility, and alter drug localization, all without having to reverse the process to allow for maintained drug function (pending suitable design). Our recent report explored the conjugation of Cbi with Ex4, to produce Cbi-Ex4 (**1**),^39^ and its effects were tested *in vivo* in the musk shrew (*Suncus murinus*), a mammal capable of emesis^41,42^ and responsive to GLP-1R targeting therapeutics.^39,^^43,44^ Our data showed that Cbi-Ex4 enhanced the glucoregulatory response following an intraperitoneal glucose tolerance test (IPGTT), without producing hypophagia, anorexia, body weight loss, and, more importantly, without emesis, all characteristics of classical Ex4-based therapies.^39^ We hypothesized that these results are due to reduced drug penetrance into the CNS (see Figure 1).^35−39^ This proof-of-concept corrinated Ex4 (**1**) was, however, notably less potent an agonist for the GLP-1R than Ex4 alone (200 vs 30 pM, respectively, in the same GLP-1R FRET assay).^39^ With that in mind, we set out to optimize **1** in terms of receptor binding and agonism for the purposes of future translation. As such, we needed to synthesize and characterize a new library of Cbi-Ex4 conjugates. Of note here is that there is a dearth of synthetic cobinamide chemistry in the literature, mostly focusing on the novel cobinamides to treat hydrogen sulfide^45,46^ or cyanide poisoning,^47−54^ and even less so in terms of conjugation chemistry (an excellent exception being that from the Gryko group using Cbi-PNA);^55^ hence, we wanted to produce Cbi-linkers that would be readily amenable to conjugation to Ex4 but indeed any targeted peptide moving forward. Here, we report a set of 16 new constructs (**12**–**27**) conjugated between Cbi and Ex4 at two positions (K12 and K40) by various linkers with a set of diverse chemical properties including hydrophobicity, amphiphilicity, and rigidity. The successful design and synthesis lead to the production of a new series of corrinated Ex4 constructs with comparable agonism and binding to unconjugated Ex4. One of these conjugates (**22**) displayed equipotency and binding at the GLP-1R *in vitro* and was subsequently selected for *ex vivo* screening for insulin secretion in rat islets and *in vivo* in shrews for glucoregulation, food intake, body weight, and emesis, compared to Ex4 and **1**.

## Results and Discussion

### Design

**Figure 2:**
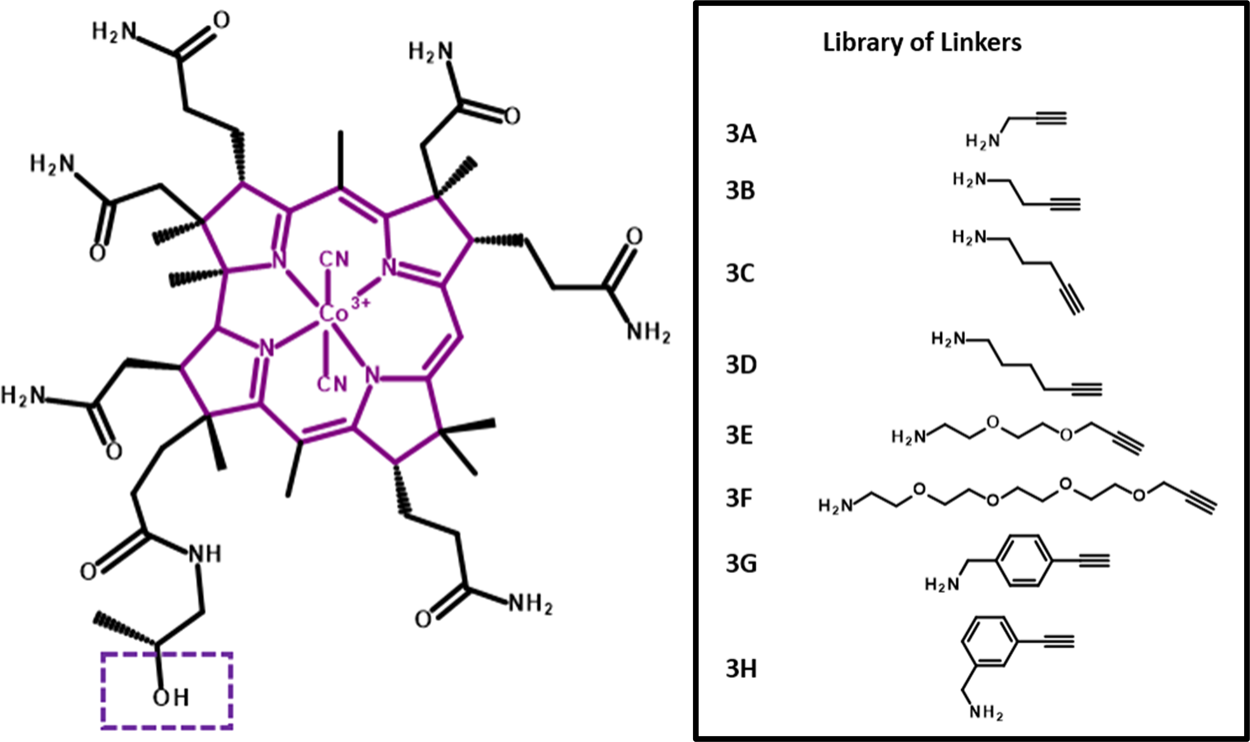
(Left) Structure of the dicyanocobinamide (CN_2_Cbi) starting material, a purple solid prepared via microwave chemistry from B12, with the hydroxyl group boxed used as a site of conjugation to linker series. (Right) Library of linkers used in the conjugation of Cbi to Ex4 peptides. The library of linkers chosen in this study included short hydrophobic alkane chains, amphiphilic PEG, and rigid substituted ethynyl phenyl methanamines, which were coupled to the Cbi hydroxyl group via CDT-mediated amide formation, resulting in Cbi compounds (**4**–**11**) with an available alkyne group for subsequent reaction with azido-modified Ex4 peptides via copper-mediated alkyne-azide click chemistry (Scheme 1).

**Scheme 1:**
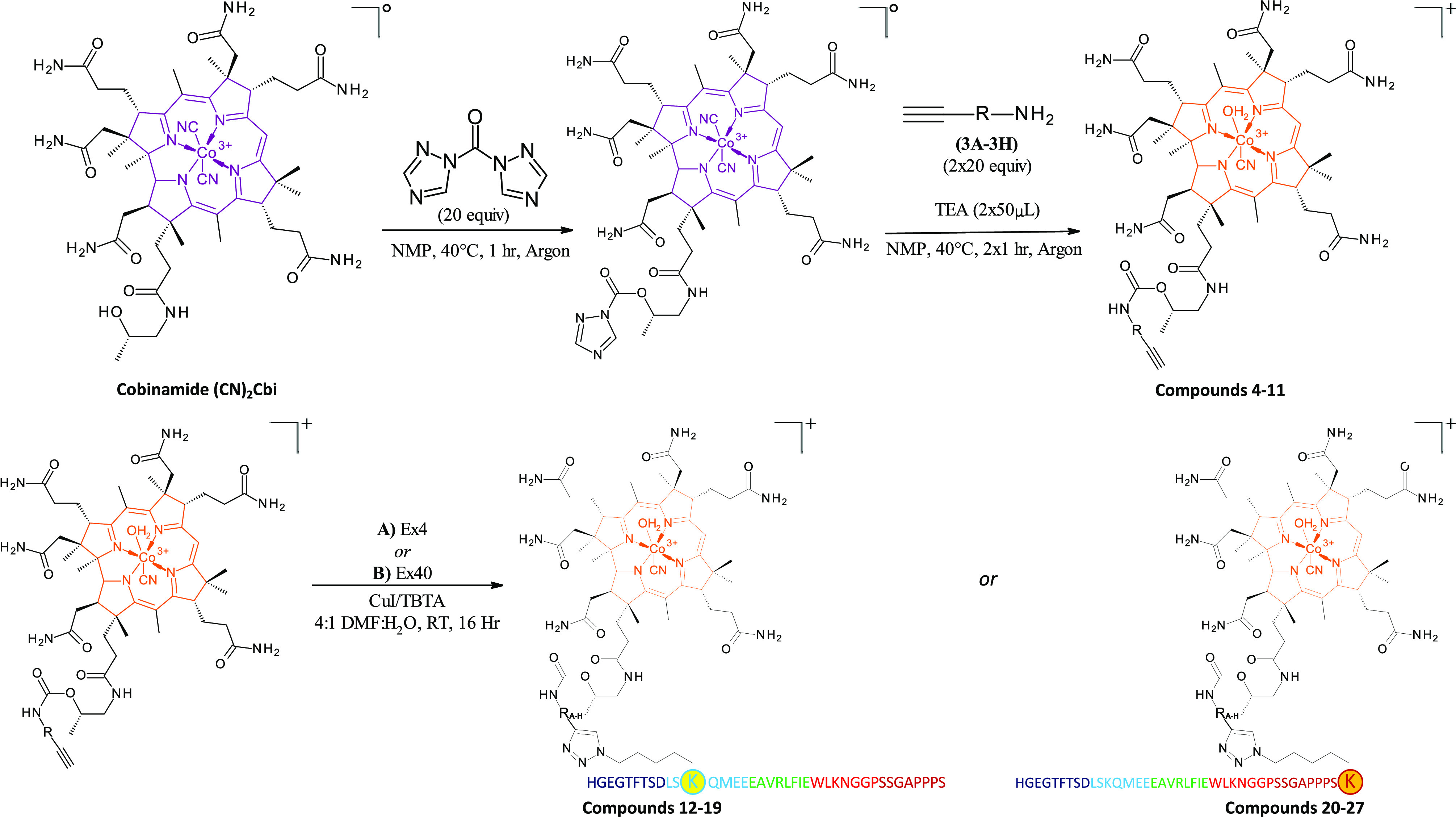
Synthesis of Cbi Linkers (**4**–**11**) and Cbi-Peptide Conjugates (**12**–**27**). Linkers **3A**–**3H** are shown in Figure 2

Our initial report on the effects of corrination on Ex4 included evidence for glucoregulation without emesis nor a reduction in food intake.^39^ In these subsequent studies, we have looked to optimize **1** with a focus on agonism and binding at the GLP-1R while also expanding to the little explored chemistry pertaining to the synthesis of Cbi conjugates. As such, the structure–activity data presented in these studies integrates synthesis, receptor agonism, receptor binding, *ex vivo* insulin secretion, *in vivo* glucoregulation, emetic response, and food intake to explore the role of the peptide conjugate site (Scheme 1) and/or the role of a specific set of spacers (linkers) as shown in Figure 2. In terms of the linkers, there are three subsets that were chosen to sample space across particular structural features, namely, steric, hydrophobic, and amphiphilic. The Ex4 conjugate site was restricted to K12 and a K40 residue added to the C-terminus of Ex4 (Ex40) since both sites were proven to be amenable to modification with minimal loss of function in our hands^35−39^ and in prior literature.^56−58^

### Chemistry

The Cbi linker compounds (**4**–**11**) and Ex4 conjugates of such (**12**–**27**) amounted to a library of 32 compounds. The experimental details are shown in Scheme 1, with linkers (Figure 2) indicated as R-groups. Initially, Cbi was produced in-house via microwave-assisted reaction of B12 with sodium cyanide in EtOH as previously described.^59^ The purple dicyanocobinamide (CN_2_Cbi) produced was then activated with an excess of 1,1′-carbonyl-di-(1,2,4-trizole) (CDT) in dry *N*-methyl-2-pyrrolidone (NMP) and heating at 40 °C under argon for ∼10 min or until complete dissolution was observed. Upon dissolution, a specific linker (Figure 2; **3A**–**3H**) was added in large excess (20×) relative to the activated CN_2_Cbi along with triethylamine (TEA), and the reaction was allowed to proceed for 1 h, again at 40 °C under argon with stirring. Initially, it was observed via RP-HPLC tracking on a C18 column (data not shown) that the reaction proceeded slowly, so in subsequent experiments, a second equivalent of linker (20×) and TEA were added at the 1 h time point and the reaction was allowed to proceed, as before, for an additional hour. Based on the additional step, yields for all Cbi-linkers were in the 30–50% range with facile purification to produce the Cbi-Linkers (**4**–**11**) with purities at, or in excess of, 97% (as tracked by RP-HPLC; see the Supporting Information). It should also be noted, and such is indicated in the color scheme used in Scheme 1 for the corrin rings, that a color change was observed upon formation of the Cbi-linkers, going from purple to orange upon purification of the Cbi-linkers in water. This color change was previously reported by Zhou and Zelder^60^ and assigned to the formation of isomers, namely, α-cyano-β-aqua- and α-aqua-β-cyano-Cbi (as indicated in Scheme 1). This suggestion of isomer formation was supported by the HPLC traces of the linkers, which clearly showed the presence of both isomers (subsequently confirmed by nuclear magnetic resonance (NMR); Figure S7). Given the fact that such isomers were not expected to negatively affect the subsequent chemistry or biology, isomers were combined prior to peptide coupling and were characterized via NMR, electrospray ionization-mass spectrometry (ESI-MS), and electronic absorption spectroscopy (EAS).

Coupling of **4**–**11** with K12-azido- or K40-azido-modified Ex4 (noted throughout here simply as Ex4 or Ex40) to produce conjugates **12**–**27** was achieved via copper(I)-mediated alkyne-azide cycloaddition over 16 h at room temperature in a 4:1 dimethylformamide (DMF)/H_2_O solvent system in the presence of the tertiary amine tris((1-benzyl-4-triazolyl)methyl)amine (TBTA) to stabilize copper(I).^61^ Yields obtained were quantitative based on the starting peptide, and all conjugates were produced to at least 97% purity prior to biological or *in vivo* evaluation (as tracked by RP-HPLC; shown in the Supporting Information).

### Structural Studies Using Circular Dichroism (CD)

**Figure 3:**
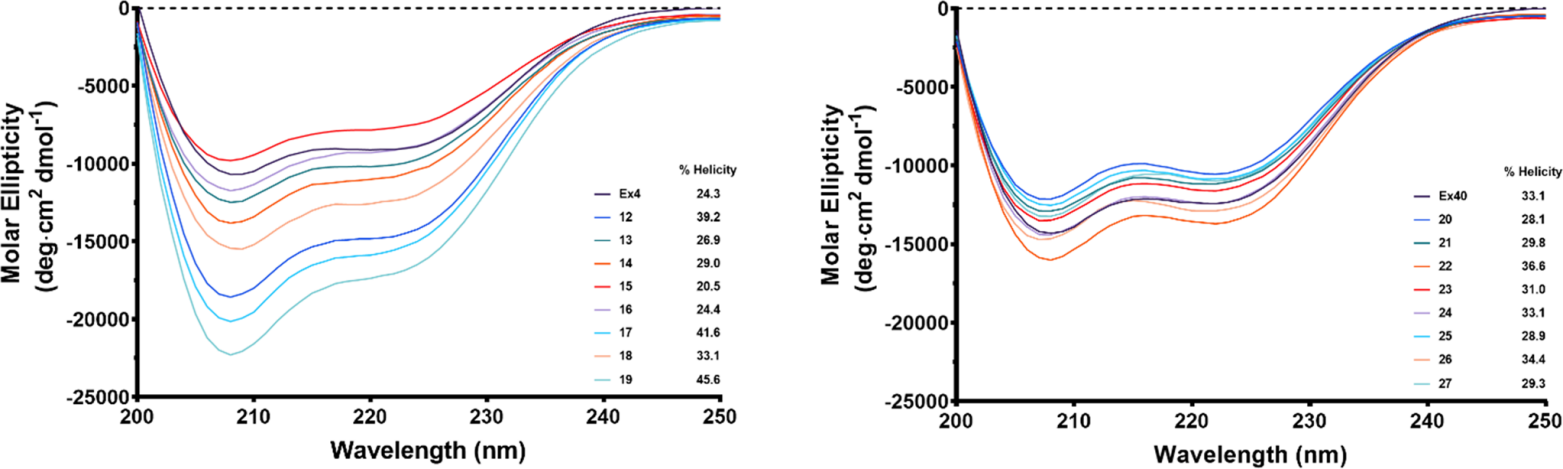
Effect of Cbi conjugation on the secondary structure of Ex4 or Ex40. CD spectra were collected with a sample concentration of 40 μM at pH 7 between 200 and 250 nm. % helicity was measured at 222 nm.

All conjugates were investigated for the effects of corrination on helicity at a concentration of 40 μM in water at pH 7.0 (Figure 3). Immediately, it was evident that there was minimal variation in percent helicity whereupon the Cbi was conjugated at the Ex4 C-terminal K40 (**20**–**27**; % helicity ranged from ∼28 to 36%; Ex40 control 33.1%; Table 1). There was however a noticeable variation in the percent helicity for the conjugates (**12**–**19**) whereupon Cbi was conjugated to the K12 within the Ex4 helix (% helicity range ∼20–46%; Ex4 control 24.3; Table 1). These results are consistent with the fact that conjugation at the C-terminus renders less consequence to the linker type or spacer distance between Cbi and the peptide, given that this region is away from the helical region of Ex4 and does not interfere with the role of this region with receptor binding/agonism. The variation noted upon conjugation at the K12 residue renders the linker critical with the highest % helicity noted as 41% with the amphiphilic PEG spacer **3F** (Figure 2, Table 1) and the lowest percent helicity noted with the small alkyl spacers **3B**–**3D** (Figure 2, Table 1). No overall correlation between the structure and agonism or binding was observed upon data fitting (not shown).

**Table 1:** EC_50_ and IC_50_ Values with Hill Slopes and % Helicity of Cbi Conjugates **12**–**27**

	EC_50_ (pM)^a^	EC_50_ Hill slope^b^	% helicity^c^	IC_50_ (nM)^d^	IC_50_ Hill slope^e^
Ex4	12.5 ± 3.7	1.1970	24.3	5.98 ± 0.94	–1.3010
**12**	75.4 ± 4.1	0.9110	39.2	24.6 ± 6.3	–1.1170
**13**	105.6 ± 4.1	1.0100	26.9	135 ± 122.5	–0.5661
**14**	33.2 ± 4.4	1.4170	29.0	79.5 ± 28.8	–0.7858
**15**	38.9 ± 15.6	1.0470	20.5	82.7 ± 15.4	–1.6990
**16**	34.1 ± 9.7	0.9976	24.4	36.5 ± 16.0	–1.1770
**17**	36.5 ± 7.6	1.0980	41.6	45.9 ± 16.3	–1.1740
**18**	40.5 ± 17.1	1.3030	33.1	34.3 ± 11.9	–1.1530
**19**	31.2 ± 16.9	1.1130	45.6	11.6 ± 2.4	–1.1270
Ex40	10.4 ± 3.9	1.1160	33.1	7.34 ± 1.37	–0.9107
**20**	13.4 ± 4.2	1.1890	28.1	26.2 ± 6.3	–1.1350
**21**	37.8 ± 11.5	1.1830	29.8	21.4 ± 8.0	–0.8940
**22**	20.7 ± 8.3	1.2090	36.6	11.9 ± 2.5	–1.3970
**23**	10.7 ± 6.0	1.0120	31.0	27.2 ± 6.3	–1.1780
**24**	16.4 ± 3.1	1.1370	33.1	15.1 ± 4.1	–1.2540
**25**	26.0 ± 14.1	0.9817	28.9	18.5 ± 4.6	–1.0840
**26**	27.9 ± 4.2	0.9957	34.4	26.4 ± 8.0	–1.2370
**27**	23.1 ± 7.1	1.2130	29.3	24.6 ± 6.3	–1.2790

a Data represents EC_50_ obtained using nonlinear regression analysis of data from highest FRET values obtained for each data point. Experiments were performed as three independent runs.

b Data represents the Hill slope obtained using nonlinear regression analysis of data from highest FRET values obtained for each data point. Experiments were performed as three independent runs.

c Data represents mean residue ellipticity [θ]_222_ determined from the CD spectra of a 40 μM solution of peptide in H_2_O at RT pH 7.0. Average [θ]_222_ values utilized to calculate percent helicity were obtained by performing the experiment in triplicate. Percent helicity was calculated using 100 × ([θ]_222_/^max^[θ]_222_). ^max^[θ]_222_ = −40,000 [1 – (2.5/*n*)], where *n* is the residue number. Experiments were performed as three independent runs.

d Data represents IC_50_ values obtained from competitive binding assays against red fluorescent GLP-1 using nonlinear regression analysis from highest values obtained for each data point. Experiments were performed as two independent runs.

e Hill slopes were obtained using nonlinear regression analysis from highest values obtained for each data point. Results are expressed as mean ± SEM.

### Biological Evaluation

To determine how the conjugation site of the peptide affects its function and the role linker choice plays in the attachment of the peptide to Cbi, we sought to screen **12**–**27** utilizing *in vitro* assays to determine potency and binding at the GLP-1R.

### *In Vitro* Functional Agonism (EC_50_) at Human GLP-1R

**Figure 4:**
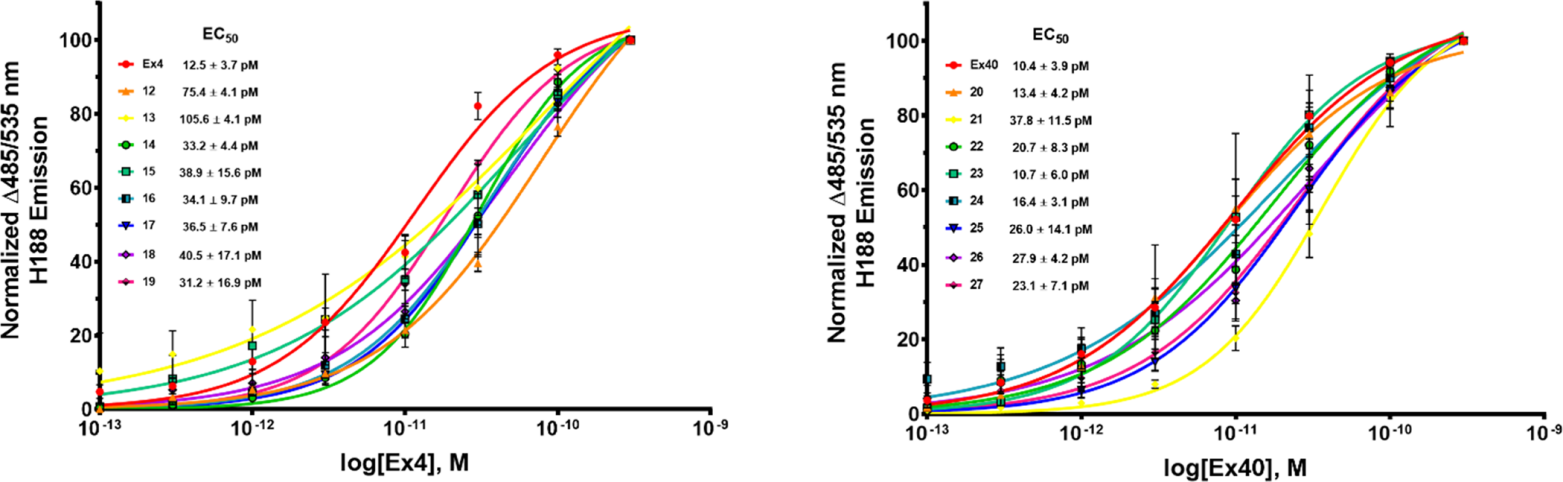
Conjugation of Cbi to Ex4 or Ex40 peptides maintains agonism at the GLP-1R. Nonlinear regression analysis was performed with GraphPad Prism 8. All compounds were assayed at least as triplicate independent runs. Data are shown as mean ± SEM.

Our previously published^39^ Cbi-Ex4 construct **1** resulted in agonism at the GLP-1R with an EC_50_ of 200 pM. The design of the newly constructed conjugates, **12**–**27**, aimed to increase the potency of the original construct to that comparable of unconjugated Ex4 (<30 pM). **12**–**27** were assessed utilizing *in vitro* screening in HEK C20 cells stably expressing the human GLP-1R and cAMP FRET reporter H188 produced in-house (see Methods).^62,63^ To determine if the azido modification to lysine at position K12 (Ex4) or the addition of this residue to position 40 (Ex40) had any effect on agonism at GLP-1R, they were also screened for agonism to compare with native Ex4 (nEx4; no azido modifications), resulting in EC_50_ values of 20 pM for nEx4, 12.5 ± 3.7 pM for Ex4, and 10.4 ± 3.9 pM for Ex40 (Table 1). All newly synthesized compounds were functional at the GLP-1R with improved potency in all cases (EC_50_ range 13.4 ± 4.2 to 105.6 ± 4.1 pM) to that of our previously reported construct **1** (200 pM; Table 1 and Figure 4). In general, the Ex4 conjugates **12**–**20** were inferior to the Ex40 conjugates (**20**–**27**) and displayed greater variance, with EC_50_ values ranging from 31 to 106 pM for **12**–**19** compared to 13 to 38 pM for **20**–**27**. As with the variance observed in the folded state for **12**–**19**, it is likely that proximity and/or interactions of the Cbi moiety to the helix interferes with receptor binding and/or interactions necessary for agonism. Conjugations performed at the C-terminal end of the peptide would be expected to place the corrin ring away from such residues as we have shown previously with B12 conjugates of the neuropeptide PYY_3–36_.^38^ One Cbi-Ex4 conjugate however (**19**), which bridged Ex4 at the K12 residue with Cbi through one of the hydrophobic, rigid linkers (**3H**), was shown to be equipotent to Ex4 with an EC_50_ value of 31.2 ± 16.9 pM and an IC_50_ of 11.6 ± 2.4 nM (compared to 5.98 ± 0.94 nM for Ex4 alone; Table 1). All Ex40-based conjugates (**20**–**27**) were shown to be equipotent to the unconjugated peptide. Such results are consistent with the fact that addition of, and conjugation to, the K40 residue is optimal when conjugating Cbi to Ex4.

### *In Vitro* Competitive Binding (IC_50_) at Human GLP-1R

As shown in Table 1, the presence of Cbi influences the ability of the conjugate to agonize the GLP-1R compared to that of Ex4 or Ex40. To further investigate how conjugation of Cbi affects Ex4 or Ex40, a series of competitive binding assays against GLP-1red (a red fluorescent analog of GLP-1) were conducted. nEx4 was utilized as a reference competitor with an IC_50_ value of 4.97 nM. We first aimed to determine if the azido modification to lysine at position K12 and the addition of this residue to position 40 had any effect on binding. It was found that both Ex4 and Ex40 had comparable binding to that of the reference compound with IC_50_ values of 5.98 ± 0.94 and 7.34 ± 1.37 nM, respectively. All conjugates had decreased binding (12–135 nM) compared to the unconjugated peptide azido-modified Ex4 (6 nM) and Ex40 (7 nM) and nEx4 controls (5 nM). When comparing the two clusters of conjugates, **20**–**27** showed greater binding affinity overall compared to **12**–**19**. The same trend was observed when agonizing the GLP-1R (Table 1) in which collectively **20**–**27** outperformed **12**–**19**. Two constructs, namely, **19** and **22**, did show promising binding with IC_50_ values of 11.6 ± 2.4 and 11.9 ± 2.5 nM, respectively. The increased binding observed in these two conjugates was consistent with their agonism (31 ± 16.9 and 20.7 ± 8.3 pM, respectively) of the GLP-1R. Based on overall binding potent agonism, facile synthesis, and high yield observed for **22**, especially related to those of **19**, we chose to further investigate **22** in *ex vivo* and *in vivo* experiments.

### Glucose-Stimulated Insulin Secretion (GSIS) in Rat Pancreatic Islets

**Figure 5:**
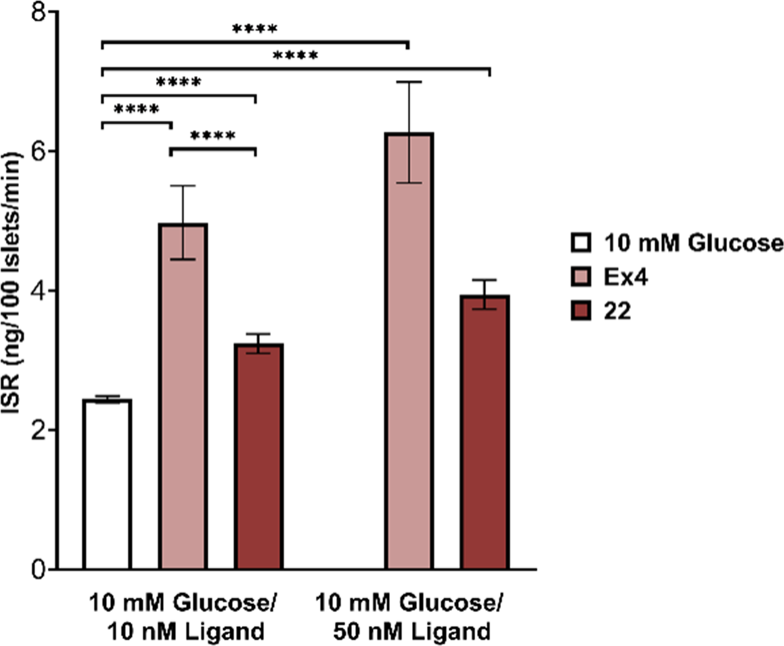
**22** increases glucose-stimulated insulin secretion in rat islets relative to glucose controls. Insulin secretion rate from static cultures of Sprague–Dawley rat islets incubated in media containing glucose (10 mM) and Ex4 (10 or 50 nM) or **22** (10 or 50 nM). Data was calculated from three independent experiments and analyzed with repeated-measurements two-way ANOVA followed by Tukey’s posthoc test. Results are expressed as mean ± SEM, **** *p* < 0.0001.

The effects of **22** on GSIS were evaluated using rat pancreatic islets (Figure 5). At 10 mM glucose, both **22** and nEx4 increased GSIS in a dose-responsive manner. The effect from **22** was observed to be one-third lower than that of nEx4. To investigate whether there was a difference between Ex40 and **22,** specific to the rat GLP-1R, we assayed each for functional (EC_50_) agonism. We observed a slight drop-off in potency with EC_50_ values of 7.8 and 22 pM recorded for Ex40 and **22** (Figures S106–S111), respectively. nEx4 control had an EC_50_ of 48 pM at the rat GLP-1R.

### *In Vivo* Functional Experiments

**Figure 6:**
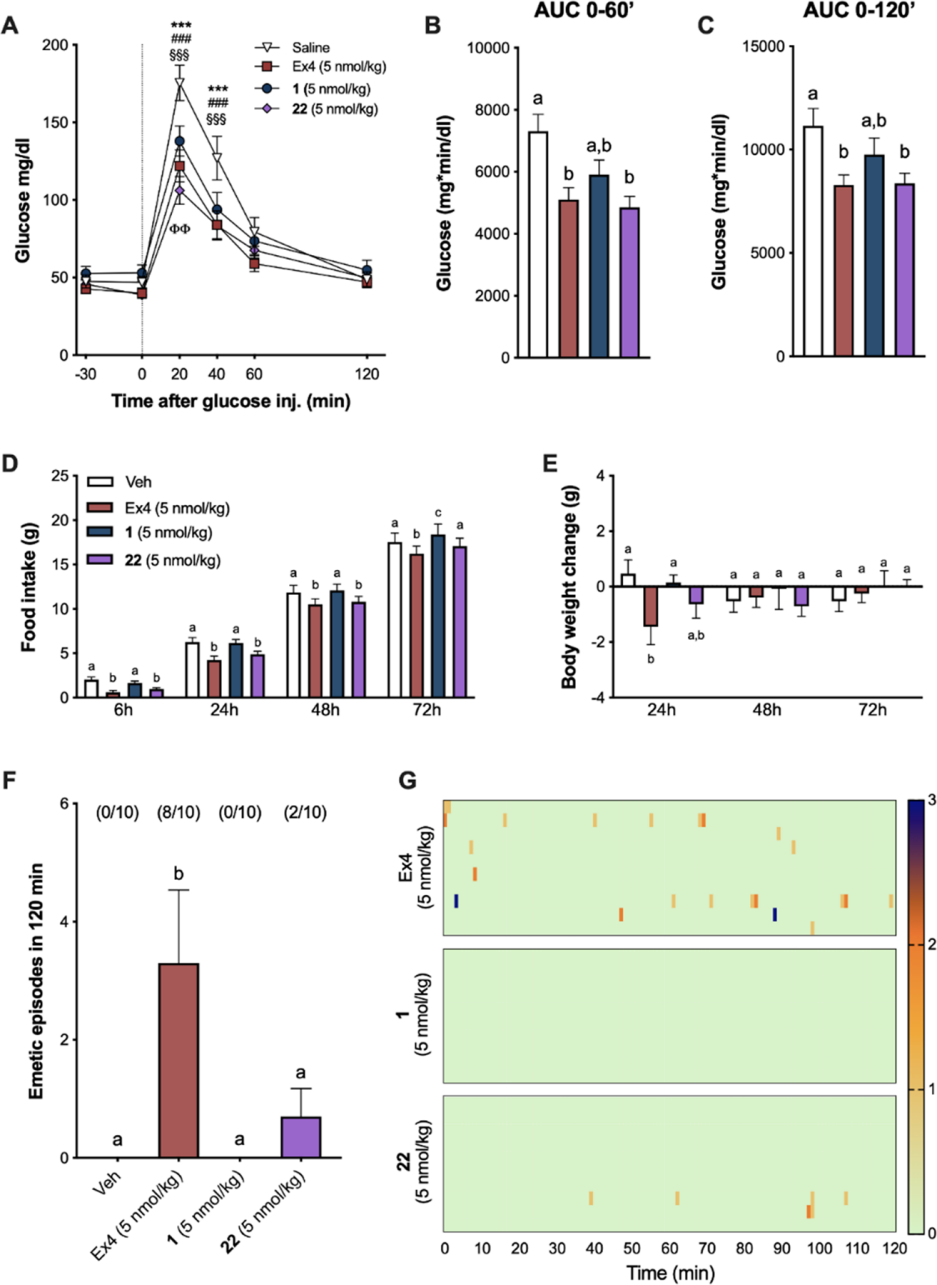
Cbi-Ex4 enhances glucose clearance without inducing emesis or body weight loss. (A) In an IPGTT, Ex4, **1**, and **22** (50 nmol/kg, IP) showed similar potency in suppressing BG levels after glucose administration (2 g/kg, IP) compared to saline; vehicle vs **1**: *** *P* < 0.001; vehicle vs **22**: ### *P* < 0.001; vehicle vs Ex4: §§§ *P* < 0.001; **1** vs **22**: ΦΦ *P* < 0.01 (*n* = 12 shrews). (B) Area under the curve (AUC) analysis from 0 (i.e., post-glucose bolus) to 60 min following **1**, **22**, and Ex4. (C) AUC analysis from 0 to 120 min; **22** and Ex4 similarly reduced AUCs compared to vehicle (*P* < 0.05). (D) Ex4 and **22** (5 nmol/kg, IP) induced anorexia at 6, 24, and 48 h (and at 72 h for Ex4 only), whereas **1** had no effect on food intake (*n* = 10). (E) Ex4-induced anorexia was accompanied by significant body weight loss at 24 h. No significant changes in body weight occurred after **1** and **22** administration compared to controls. (F) The number of single emetic episodes following Ex4, **1**, **22**, or saline systemic administration was recorded for 120 min. The number of animals exhibiting emesis, expressed as a fraction of the total number of animals tested, is indicated above each treatment group. Ex4 induced robust emetic responses that were not observed after **1** or saline injections. **22** induced emesis in two of the animals tested; however, the number of emetic episodes was significantly lower than that of Ex4 and did not differ from animals treated with vehicle or **1** (*n* = 10). (G) Heatmaps showing latency, number, and intensity of emesis following Ex4, **1**, and **22** dosing for each individual animal across time. All data expressed as mean ± SEM (*n* = 10). Data in panels (A), (D), and (E) were analyzed with repeated-measurements two-way ANOVA followed by Tukey’s posthoc test. Data in panels (B), (C), and (F) were analyzed with repeated-measurements one-way ANOVA followed by Tukey’s posthoc test. Means with different letters are significantly different (*P* < 0.05).

To functionally compare the activity of the corrinated conjugates *in vivo*, we first tested the effects of Ex4, **1** (the original proof-of-concept conjugate),^39^ and **22** on plasma glucose levels following an IPGTT in shrews. A shrew is a mammal capable of emesis^41,42^ with published sensitivity to GLP-1RAs^39,43,44^ and the same serum B12 binding proteins (IF and Haptocorrin) as that found in humans^39^ and thus was considered an ideal model for the *in vivo* studies. We observed that shrews treated with **1**, **22**, or nEx4 display similar improvements in glucose handling following glucose load compared to vehicle controls (Figure 6A). Posthoc analyses showed that all three compounds significantly suppressed blood glucose (BG) at 20 and at 40 min after glucose administration versus vehicle treatment (all *p* < 0.001). Remarkably, BG values at 20 min post glucose injection in animals receiving **22** were significantly lower than those in animals treated with **1**, denoting an improved glucose tolerance/superior pharmacological efficacy (*p* < 0.01). Additionally, the variation in plasma glucose concentrations, represented as area under the curves (0–60′ and 0–120′, respectively) for **22**, did not differ from nEx4 and were significantly lower than controls (Figure 6B,C, all *p* < 0.05).

We then analyzed the effects of **1**, **22**, and Ex4 on food intake and body weight at a single, proof-of-concept, dose, in line with previous reports.^35−37,39^ Ex4 administration produced anorexia in shrews at all measured time points (Figure 6D), while **1** had no impact on feeding. **22** treatment suppressed food intake similar to Ex4 at 6, 24, and 48 h. The anorectic effect of Ex4 treatment was paralleled by a significant reduction in body weight at 24 h (*p* < 0.05), which was not observed following treatment with **1** or **22** (Figure 6E).

To test whether **22** treatment retained the non-emetic properties of the lead conjugate **1**, indicative of an altered pharmacodynamic profile, despite its comparable potency and GLP-1R binding affinity compared to **1**, and comparable to nEx4, we compared the emetogenic properties of **1**, **22**, and native Ex4 in shrews. Ex4 induced profound emesis in the majority of the shrews tested (Figure 6F). Indeed, 80% of the animals exhibited emesis upon administration of native Ex4 within minutes after injection (29 ± 16 min). In line with a previous report, treatment with **1** did not cause emesis in any of the shrews tested.^39^ Importantly, the number of single emetic episodes occurring in the 120 min window after drug administration was also significantly reduced after **22** administration compared to Ex4 and did not differ from vehicle- or **1**-treated groups. Only two animals that received **22** experienced emesis with an average latency of 70 ± 29 min. The greater emetic effect observed for **22** over **1** is likely a consequence of the greater binding and agonism of **22** at the GLP-1R, an effect that is likely to be mitigated, without loss of glucoregulation, by use of lower doses of **22** (on-going work). The difference in the emetogenic profiles of the corrinated constructs versus native Ex4 is further emphasized by the graphical visualization of emetic intensity, recurrence, and latency for each individual animal across time (Figure 6G).

## Conclusions

Neuroendocrine associated pharmacological side effects such as nausea and emesis are often downplayed or dismissed, left in the shadow of the overriding target, be it glucoregulation, weight loss, *etc*. Indeed, the weight loss gleaned from GLP-1RAs, for example, has proven to be a beneficial “side effect” of T2D treatment due to the high comorbidity of T2D with obesity.^64^ In many cases, however, such as lean T2D patients, or patients with comorbidities where nutritional status is critical (cystic fibrosis, cancer-cachexia, sarcopenia, chronic obstructive pulmonary disease, *etc.*), removal of the CNS-associated side effects would be greatly beneficial. Indeed, GLP-1RAs have proven to be life-altering, and the ability to expand their use, or increase patient compliance when using, should not be understated. Herein, we conceived 16 new Cbi-Ex4/Ex40 conjugates with the aim of designing a construct with equipotent binding and agonism of the GLP-1R to that of Ex4 with a view of maintaining activity at peripheral GLP-1R populations and mitigating such activity in CNS populations. We were able to successfully design several constructs with comparable binding and agonism at GLP-1R as Ex4, with one conjugate, **22** (IC_50_ 11.9 ± 2.5 nM, EC_50_ 20.7 ± 8.3 pM), translated into *ex vivo* and *in vivo* studies. **22** increased glucose secretion compared to vehicle controls in a glucose-dependent insulin secretion assay in rat islets. In an *in vivo* IPGTT, **22** provided glucoregulation comparable to Ex4. Critically, **22** showed a near absence of emesis and mild body weight lowering actions compared to profound emesis and body weight loss observed for nEx4.

While corrination is poorly explored, especially as it pertains to applications that seek to prevent CNS penetration while maintaining peripheral activity of a target drug, it shows considerable promise as a platform technology, moving beyond GLP-1RAs. An additional highlight of Cbi is its water solubility (400 mg/mL), akin to glucose (450 mg/mL). Use of Cbi conjugation with drugs with physical properties not suited to drug development, such as glucagon^65−67^ for rescue of hypoglycemia, offers a great scope for exploration.

## Experimental Section

### General Considerations

All commercial reagents and anhydrous solvents purchased were used without further purification. All reactions containing air- and/or moisture-sensitive reactants were performed under argon. Compounds were purified using a Shimadzu Prominence HPLC using a C18 column (Eclipse XDB-C18 5 μm, 4.6 × 150 mm). All compounds had purities at, or in excess of, 97% (as tracked by RP-HPLC; see the Supporting Information). Products were analyzed for mass using a Shimadzu LCMS-8040. ^1^H NMR and ^13^C NMR spectra were acquired on a Bruker NMR spectrometer (Avance 400 MHz) in D_2_O at a D1 of 3 for all compounds. EAS was collected on a Varian Cary 50bio UV–visible spectrophotometer in a 1 mL quartz cuvette with baseline correction. CD spectra were recorded with a Jasco J-715 circular dichroism spectropolarimeter in 40 μM H_2_O in a 0.1 cm quart cuvette. Data was analyzed and fit using GraphPad Prism 8.0. We established a new clone of HEK293 cells designated here as HEK293-GLP-1R-H188-C20. This clone was obtained by G418 antibiotic resistance selection after cotransfection of cells with the human GLP-1R and the cAMP FRET reporter H188. Cells were grown in a Memmert Incubator I (Schwabach, Germany) at 37 °C gassed with 5% CO_2_ at ∼95% humidity. FRET assays were conducted utilizing a Molecular Devices FlexStation 3 Multi-Mode Microplate Reader. Data was analyzed utilizing Molecular Devices SoftMax Pro v.5.4.6. Linear regression analyses were performed utilizing GraphPad Prism 8.

### Materials

The following were purchased from Sigma Aldrich: acetonitrile (Cat # 14851), diethyl ether (Cat # 673811), ethanol (Cat # 459844), ethyl acetate (Cat # 319902), isopropyl alcohol (Cat # 34863), methanol (Cat # 34885), *n*-methyl-2-pyrrolidone (Cat # 328634), triethylamine (Cat # 8.08352), vitamin B_12_ (Cat # V2876), sodium cyanide (Cat # 205222), 1,1′-carbonyl-di-(1,2,4-trizole) (Cat # 21863), propargylamine (Cat # P50900), 1-amino-3-butyne (Cat # 715190), 4-pentyne-1-amine (Cat # 779407), hex-5-yne-1-amine (Cat # COM497512576), bovine serum albumin (Cat # A7030), Dulbecco’s modified Eagle medium (Cat # D6429), fetal bovine serum (Cat # 12303C), G-418 disulfate salt solution (Cat # G8168), and HEPES (Cat # H0887). The following were purchased from ThermoFisher Scientific: penicillin–streptomycin (Cat # 15140122) and trypsin–EDTA (Cat # 25200072). The following were purchased from Fisher Scientific: calcium chloride dihydrate (Cat # BP510), magnesium chloride hexahydrate (Cat # BP214), potassium chloride (Cat # BP366), and sodium chloride (Cat # BP358–1). The following were purchased from BroadPharm: propargyl-PEG2-amine (Cat # BP22519) and propargyl-PEG4-amine (Cat # BP22520). The following were purchased from Enamine: (4-ethynylphenyl)methanamine (Cat # EN300–233648) and (3-ethynylphenyl)methanamine (Cat # EN300–248722). Krebs-Ringer Bicarbonate (KRB) buffer was prepared as described previously.^68^d-Glucose (Cat # G 5767) and nEx4 (Cat # E7144) were purchased from Sigma-Aldrich. Ex4 K12-azido and Ex40 K40-azido were produced by Genscript (Piscataway, New Jersey, United States). The rat insulin radioimmunoassay (RIA) kit was purchased from Millipore Sigma, Burlington, MA Cat no. RI-13 K. The human GLP-1R plasmid was provided by M. Beinborn, Tufts University School of Medicine to author G.G.H.^62^ The H188 plasmid was provided by K. Jalink, Netherlands Cancer Institute to authors G.G.H. and R.P.D.^63^

### Methods

#### Synthesis of Dicyanocobinamide (Cbi) (**2**)

The synthesis of dicyanocobinamide (Cbi) was performed according to previously reported methods.^59^ Briefly, to a 10 mL microwave reaction vessel containing a magnetic stir bar were added B12 (cyanocobalamin) (303.8 mg, 0.225 mmol) (see Figures S1–S6), sodium cyanide (NaCN) (36.9 mg, 0.7096 mmol), and 5 mL of EtOH, and the vessel was sealed. The reaction was heated to 120 °C for 10 min. Following completion of the microwave reaction, the remaining solution was transferred and diluted using isopropyl alcohol (iPrOH). The reaction was purified utilizing a normal-phase silica column. The reaction was eluted using 100% methanol (MeOH). The product eluted as a purple product. The isolated product was precipitated utilizing diethyl ether (Et_2_O) and allowed to dry producing a solid, purple product, **2** (see Figure 2), in 80% yield (187.4 mg, 0.1795 mmol). Solubility was measured to be 400 mg/mL in distilled water. **2** was purified by RP-HPLC as described for method A1, to 98%; *t*_R_: 5.2 and 6.7 min; ESI-MS expected *m*/*z* = 1042, observed *m*/*z* = [M–CN]^+1^ 1016. ^1^H NMR (400 MHz, D_2_O): δ 5.90 (s, 1H), 3.95–3.87 (m, 1H), 3.85 (d, *J* = 8.4. Hz, 1H), 3.75 (d, *J* = 10.4 Hz, 1H), 3.41 (m, 1H), 3.36 (s, 5H), 3.30 (m, 2H), 3.25 (d, *J* = 4.7 Hz, 1H), 3.17 (dd, *J* = 6.8, 6.9 Hz, 1H), 2.92–2.86 (m, 1H), 2.75–2.73 (m, 2H), 2.65–2.39 (m, 7H), 2.32–2.26 (m, 15H), 2.19–2.08 (m, 5H), 2.03–1.95 (m, 1H), 1.91–1.76 (4H), 1.69 (s, 3H), 1.54 (s, 3H), 1.50 (s, 3H), 1.44 (s, 3H), 1.32 (s, 3H), 1.19 (s, 4H), 1.16 (d, *J* = 6.4 Hz, 4H). ^13^C NMR (400 MHz, D_2_O): 178.6, 178.4, 177.9, 177.5, 177.3, 176.8, 175.1, 175.0, 172.1, 163.4, 162.9, 105.1, 103.1, 91.0, 83.1, 75.0, 66.2, 64.2, 58.8, 56.3, 55.2, 53.2, 49.2, 48.9, 46.8, 46.3, 46.1, 43.7, 42.2, 39.0, 34.9, 32.5, 32.4, 31.9, 31.7, 31.5, 30.5, 26.6, 25.7, 24.9, 23.8, 21.7, 19.4, 18.7, 18.5, 17.4, 16.5, 15.1, 14.8. See Figures S7–S11.

#### General Procedure for Cobinamide Linker Coupling

To a 5 mL round bottom flask containing a stir bar were added **2** (15.8 mg, 0.0152 mmol) and 1 mL of NMP, and the solution was allowed to stir at 40 °C under argon until the starting material was fully dissolved. To the stirring solution of **2** was added CDT (51.4 mg, 0.313 mmol, ∼20 equiv.), and the solution was allowed to stir for 1 h at which time one of the **3A**–**3H** (see Figure 2) (19.2 μL, 16.5 mg, 0.300 mmol, ∼20 equiv.) and triethylamine (TEA) (50 μL) were added. Upon stirring for an additional hour, a second equivalent of linker (**3A**–**3H**) and TEA were added, and the solution was stirred for 1 h. The reaction was then poured into ethyl acetate (AcOEt) (50 mL) and centrifuged (5 min, 4000 rpm, RT). The crude solid was redissolved in a minimal amount of MeOH (∼5 mL) and precipitated with diethyl ether (Et_2_O) and centrifuged. The crude product was redissolved in 1 mL of deionized (DI) H_2_O and purified utilizing RP-HPLC as described below.

#### HPLC Purification Methods

**4**–**27** were purified using a Shimadzu Prominence HPLC using a C18 column (Eclipse XDB-C18 5 μm, 4.6 × 150 mm). Two differing purification methods were used: A1, H_2_O + 0.1% TFA and MeOH from 1% CH_3_OH/H_2_O + 0.1% TFA to 90% CH_3_OH/H_2_O + 0.1% TFA in 25 min; A2, H_2_O + 0.1% TFA and CH_3_CN from 1% CH_3_CN/H_2_O + 0.1% TFA to 70% CH_3_CN/H_2_O + 0.1% TFA in 15 min.

##### Cbi-Propyne (**4**)

**4** was prepared according to the general procedure described above, combining **2** (16.8 mg, 0.0161 mmol) with CDT (51.4 mg, 0.3000 mmol) and stirring the solution for 1 h at which time **3A** (19.2 μL, 16.5 mg, 0.300 mmol) and TEA (50 μL) were added and stirred for 1 h after which point a second equivalent amount of **3A** and TEA were added and allowed to stir for 1 h to give the target compound, which was purified using RP-HPLC method A1 to produce **4** as an orange solid to 97% purity. Yield 43% (7.8 mg, 0.007 mmol). The product obtained was in the form of two different isomers with the aquo-group located on the alpha and beta positions (α-cyano-β-aqua- and α-aqua-β-cyano-). *t*_R_: 8.8 and 9.5 min; ESI-MS expected *m*/*z* = 1115, observed *m*/*z* = [M^+^–H_2_O]^+1^: 1096, [M^+^–H_2_O + H^+^]^+2^: 549. ^1^H NMR (400 MHz, D_2_O): δ 6.50 (1H, s, Ar-H, β-aqua isomer) 6.42 (1H, s, Ar-H, α-aqua isomer). EAS Ext Coeff_354_ = 14,500 M^–1^ cm^–1^. See Figures S18–S22.

##### Cbi-Butyne (**5**)

**5** was prepared according to the general procedure described above, combining **2** (20.5 mg, 0.0197 mmol) with CDT (61.3 mg, 0.3740 mmol) and stirring the solution for 1 h at which time **3B** (32.0 μL, 27.0 mg, 0.391 mmol) and TEA (50 μL) were added and stirred for 1 h after which point a second equivalent of **3B** and TEA were added and allowed to stir for 1 h to give the target compound, which was purified using RP-HPLC method A1 to produce **5** as an orange solid to 95% purity. Yield 53% (11.4 mg, 0.010 mmol). The product obtained was in the form of two different isomers with the aquo-group located on the alpha and beta positions (α-cyano-β-aqua- and α-aqua-β-cyano-). *t*_R_: 7.4 and 7.9 min; ESI-MS expected *m*/*z* = 1129, observed *m*/*z* = [M^+^–H_2_O]^+1^: 1111, [M^+^–H_2_O + H^+^]^+2^: 556. ^1^H NMR (400 MHz, D_2_O): δ 6.49 (1H, s, Ar-H, β-aqua isomer) 6.42 (1H, s, Ar-H, α-aqua isomer). EAS Ext Coeff_354_ = 19,804 M^–1^ cm^–1^. See Figures S29–S33.

##### Cbi-Pentyne (**6**)

**6** was prepared according to the general procedure described above, combining **2** (17.3 mg, 0.0166 mmol) with CDT (49.1 mg, 0.299 mmol) and stirring the solution for 1 h at which time **3C** (29.0 μL, 24.9 mg, 0.300 mmol) and TEA (50 μL) were added and stirred for 1 h after which point a second equivalent amount of **3C** and TEA were added and allowed to stir for 1 h to give the target compound, which was purified using RP-HPLC method A1 to produce **6** as an orange solid to 97% purity. Yield 21% (4.0 mg, 0.003 mmol). The product obtained was in the form of two different isomers with the aquo-group located on the alpha and beta positions (α-cyano-β-aqua- and α-aqua-β-cyano-). *t*_R_: 10.4 and 11.0 min; ESI-MS-expected *m*/*z* = 1143, observed *m*/*z* = [M^+^–H_2_O]^+1^: 1124, [M^+^–H_2_O + H^+^]^+2^: 563. ^1^H NMR (400 MHz, D_2_O): δ 6.50 (1H, s, Ar-H, β-aqua isomer) 6.43 (1H, s, Ar-H, α-aqua isomer). EAS Ext Coeff_354_ = 15,001 M^–1^ cm^–1^. See Figures S40–S44.

##### Cbi-Hexyne (**7**)

**7** was prepared according to the general procedure described above, combining **2** (15.8 mg, 0.0151 mmol) with CDT (51.6 mg, 0.314 mmol) and stirring the solution for 1 h at which time **3D** (36.5 μL, 29.2 mg, 0.300 mmol) and TEA (50 μL) were added and stirred for 1 h after which point a second equivalent amount of **3D** and TEA were added and allowed to stir for 1 h to give the target compound, which was purified using RP-HPLC method A1 to produce **7** as an orange solid to 98% purity. Yield 35% (6.0 mg, 0.005 mmol). The product obtained was in the form of two different isomers with the aquo-group located on the alpha and beta positions (α-cyano-β-aqua- and α-aqua-β-cyano-). *t*_R_: 11.3 and 11.8 min; ESI-MS-expected *m*/*z* = 1157, observed *m*/*z* = [M^+^–H_2_O]^+1^: 1139, [M^+^–H_2_O + H^+^]^+2^: 570. ^1^H NMR (400 MHz, D_2_O): δ 6.49 (1H, s, Ar-H, β-aqua isomer) 6.42 (1H, s, Ar-H, α-aqua isomer). EAS Ext Coeff_354_ = 17,982 M^–1^ cm^–1^. See Figures S51–S55.

##### Cbi-PEG2-Alkyne (**8**)

**8** was prepared according to the general procedure described above, combining **2** (19.9 mg, 0.0191 mmol) with CDT (67.1 mg, 0.409 mmol) and stirring the solution for 1 h at which time **3E** (25.1 μL, 25.4 mg, 0.177 mmol) and TEA (50 μL) were added and stirred for 1 h after which point a second equivalent amount of **3E** and TEA were added and allowed to stir for 1 h to give the target compound, which was purified using RP-HPLC method A1 to produce **8** as an orange solid to 98% purity. Yield 39% (8.8 mg, 0.007 mmol). The product obtained was in the form of two different isomers with the aquo-group located on the alpha and beta positions (α-cyano-β-aqua- and α-aqua-β-cyano-). *t*_R_: 18.4 and 19.3 min; ESI-MS-expected *m*/*z* = 1204, observed *m*/*z* = [M^+^–H_2_O]^+1^: 1185, [M^+^–H_2_O + H^+^]^+2^: 593. ^1^H NMR (400 MHz, D_2_O): δ 6.50 (1H, s, Ar-H, β-aqua isomer) 6.43 (1H, s, Ar-H, α-aqua isomer). EAS Ext Coeff_354_ = 22,919 M^–1^ cm^–1^. See Figures S62–S66.

##### Cbi-PEG4-Alkyne (**9**)

**9** was prepared according to the general procedure described above, combining **2** (15.5 mg, 0.0149 mmol) with CDT (51.3 mg, 0.313 mmol) and stirring the solution for 1 h at which time **3F** (18.8 μL, 19.5 mg, 0.0843 mmol) and TEA (50 μL) were added and stirred for 1 h after which point a second equivalent amount of **3F** and TEA were added and allowed to stir for 1 h to give the target compound, which was purified using RP-HPLC method A1 to produce **9** as an orange solid to 97% purity. Yield 32% (6.1 mg, 0.005 mmol). The product obtained was in the form of two different isomers with the aquo-group located on the alpha and beta positions (α-cyano-β-aqua- and α-aqua-β-cyano-). *t*_R_: 20.0 and 21.8 min; ESI-MS-expected *m*/*z* = 1292, observed *m*/*z* = [M^+^–H_2_O]^+1^: 1273, [M^+^–H_2_O + H^+^]^+2^: 637. ^1^H NMR (400 MHz, D_2_O): δ 6.50 (1H, s, Ar-H, β-aqua isomer) 6.43 (1H, s, Ar-H, α-aqua isomer). EAS Ext Coeff_354_ = 19,439 M^–1^ cm^–1^. See Figures S73–S77.

##### Cbi-4EPMA-Alkyne (**10**)

**10** was prepared according to the general procedure described above, combining **2** (14.9 mg, 0.0143 mmol) with CDT (50.1 mg, 0.305 mmol) and stirring the solution for 1 h at which time **3G** (23.8 mg, 0.181 mmol) and TEA (50 μL) were added and stirred for 1 h after which point a second equivalent amount of **3G** and TEA were added and allowed to stir for 1 h to give the target compound, which was purified using RP-HPLC method A1 to produce **10** as an orange solid to 97% purity. Yield 10% (1.5 mg, 0.001 mmol). The product obtained was in the form of two different isomers with the aquo-group located on the alpha and beta positions (α-cyano-β-aqua- and α-aqua-β-cyano-). *t*_R_: 12.4 and 12.8 min; ESI-MS-expected *m*/*z* = 1191, observed *m*/*z* = [M^+^–H_2_O]^+1^: 1173. ^1^H NMR (400 MHz, D_2_O): δ 6.49 (1H, s, Ar-H, β-aqua isomer) 6.43 (1H, s, Ar-H, α-aqua isomer). EAS Ext Coeff_354_ = 15,886 M^–1^ cm^–1^. See Figures S84–S88.

##### Cbi-3EPMA-Alkyne (**11**)

**11** was prepared according to the general procedure described above, combining **2** (15.7 mg, 0.0151 mmol) with CDT (51.1 mg, 0.311 mmol) and stirring the solution for 1 h at which time **3H** (21.0 μL, 21.2 mg, 0.162 mmol) and TEA (50 μL) were added and stirred for 1 h after which point a second equivalent amount of **3H** and TEA were added and allowed to stir for 1 h to give the target compound, which was purified using RP-HPLC method A1 to produce **11** as an orange solid to 97% purity. Yield 14% (2.6 mg, 0.002 mmol). The product obtained was in the form of two different isomers with the aquo-group located on the alpha and beta positions (α-cyano-β-aqua- and α-aqua-β-cyano-). *t*_R_: 12.6 and 13.0 min; ESI-MS-expected *m*/*z* = 1191, observed *m*/*z* = [M^+^–H_2_O]^+1^: 1172, [M^+^–H_2_O + H^+^]^+2^: 587. ^1^H NMR (400 MHz, D_2_O): δ 6.49 (1H, s, Ar-H, β-aqua isomer) 6.42 (1H, s, Ar-H, α-aqua isomer). EAS Ext Coeff_354_ = 15,669 M^–1^ cm^–1^. See Figures S95–S99.

#### General Procedure for Copper-Mediated Alkyne-Azide

##### Cycloaddition

To a 5 mL round bottom flask containing a stir bar, copper(I) iodide (CuI) (3.3 mg, 0.017 mmol) and TBTA (7.0 mg, 0.013 mmol) were added to 1 mL of 4:1 DMF/H_2_O. The reaction mixture was allowed to stir at room temperature until a color change occurred (clear to yellowish brown) (∼15 min). The solution was treated with azido-exendin-4/40 (2.0 mg, 0.0004 mmol) (see Figures S12–S17) and the corresponding Cbi-alkynes (**4**–**11**) (4.8 mg, 0.004 mmol) and allowed to stir overnight.

##### Cbi-Pro-Ex4 (**12**)

**12** was prepared according to the general procedure described above; combining **4** (1.1 mg, 0.001 mmol) with CuI (4.0 mg, 0.021 mmol), TBTA (7.5 mg, 0.014 mmol), and Ex4 (3.1 mg 0.0007 mmol) gave the target compound, which was purified using RP-HPLC method A2 to produce **12** as a red solid to 98% purity. *t*_R_: 11.7 min; ESI-MS-expected *m*/*z* = 5327, observed *m*/*z* = [M^+^–H_2_O + 2H^+^]^+3^: 1771, [M^+^–H_2_O + 3H^+^]^+4^: 1328. See Figures S23–S25.

##### Cbi-But-Ex4 (**13**)

**13** was prepared according to the general procedure described above; combining **5** (5.7 mg, 0.005 mmol) with CuI (4.2 mg, 0.022 mmol), TBTA (7.5 mg, 0.014 mmol), and Ex4 (4.2 mg 0.0010 mmol) gave the target compound, which was purified using RP-HPLC method A2 to produce **13** as a red solid to 98% purity. *t*_R_: 12.0 min; ESI-MS-expected *m*/*z* = 5341, observed *m*/*z* = [M^+^–H_2_O + 2H^+^]^+3^: 1775, [M^+^–H_2_O + 3H^+^+CH_3_OH]^+4^: 1364, [M^+^–H_2_O + 3H^+^]^+4^: 1332. See Figures S34–S36.

##### Cbi-Pent-Ex4 (**14**)

**14** was prepared according to the general procedure described above; combining **6** (4.9 mg, 0.0043 mmol) with CuI (3.6 mg, 0.019 mmol), TBTA (6.2 mg, 0.012 mmol), and Ex4 (3.9 mg 0.0009 mmol) gave the target compound, which was purified using RP-HPLC method A2 to produce **14** as a red solid to 98% purity. *t*_R_: 12.0 min; ESI-MS-expected *m*/*z* = 5355, observed *m*/*z* = [M^+^–H_2_O + 2H^+^]^+3^: 1780, [M^+^–H_2_O + 3H^+^]^+4^: 1335. See Figures S45–S47.

##### Cbi-Hex-Ex4 (**15**)

**15** was prepared according to the general procedure described above; combining **7** (9.7 mg, 0.0084 mmol) with CuI (3.5 mg, 0.018 mmol), TBTA (6.6 mg, 0.012 mmol), and Ex4 (5.3 mg 0.0013 mmol) gave the target compound, which was purified using RP-HPLC method A3 to produce **15** as a red solid to 98% purity. *t*_R_: 12.0 min; ESI-MS-expected *m*/*z* = 5369, observed *m*/*z* = [M^+^–H_2_O + 2H^+^]^+3^: 1784, [M^+^–H_2_O + 3H^+^]^+4^: 1339. See Figures S56–S58.

##### Cbi-PEG2-Ex4 (**16**)

**16** was prepared according to the general procedure described above; combining **8** (2.0 mg, 0.0017 mmol) with CuI (3.6 mg, 0.019 mmol), TBTA (7.1 mg, 0.013 mmol), and Ex4 (3.0 mg 0.0007 mmol) gave the target compound, which was purified using RP-HPLC method A2 to produce **16** as a red solid to 98% purity. *t*_R_: 11.7 min; ESI-MS-expected *m*/*z* = 5416, observed *m*/*z* = [M^+^–H_2_O + 2H^+^]^+3^: 1800, [M^+^–H_2_O + 3H^+^]^+4^: 1350. See Figures S67–S69.

##### Cbi-PEG4-Ex4 (**17**)

**17** was prepared according to the general procedure described above; combining **9** (3.0 mg, 0.0023 mmol) with CuI (3.4 mg, 0.018 mmol), TBTA (7.3 mg, 0.014 mmol), and Ex4 (4.1 mg 0.0010 mmol) gave the target compound, which was purified using RP-HPLC method A2 to produce **17** as a red solid to 98% purity. *t*_R_: 12.3 min; ESI-MS-expected *m*/*z* = 5504, observed *m*/*z* = [M^+^–H_2_O + 2H^+^ + CH_3_CN]^+3^: 1870, [M^+^–H_2_O + 3H^+^ + CH_3_OH]^+4^: 1404, [M^+^–H_2_O + 4H^+^]^+5^: 1098. See Figures S79–S80.

##### Cbi-4EPMA-Ex4 (**18**)

**18** was prepared according to the general procedure described above; combining **10** (3.3 mg, 0.0028 mmol) with CuI (3.6 mg, 0.019 mmol), TBTA (7.2 mg, 0.014 mmol), and Ex4 (3.0 mg 0.0007 mmol) gave the target compound, which was purified using RP-HPLC method A2 to produce **18** as a red solid to 98% purity. *t*_R_: 12.2 min; ESI-MS-expected *m*/*z* = 5403, observed *m*/*z* = [M^+^–H_2_O + 2H^+^]^+3^: 1796, [M^+^–H_2_O + 3H^+^]^+4^: 1347. See Figures S89–S91.

##### Cbi-3EPMA-Ex4 (**19**)

**19** was prepared according to the general procedure described above; combining **11** (3.0 mg, 0.0025 mmol) with CuI (4.5 mg, 0.024 mmol), TBTA (7.6 mg, 0.014 mmol), and Ex4 (2.7 mg 0.0006 mmol) gave the target compound, which was purified using RP-HPLC method A2 to produce **19** as a red solid to 97% purity. *t*_R_: 12.6 min; ESI-MS-expected *m*/*z* = 5403, observed *m*/*z* = [M^+^–H_2_O + 2H^+^]^+3^: 1796, [M^+^–H_2_O + 2H^+^ + CH_3_OH]^+3^:1379, [M^+^–H_2_O + 3H^+^]^+4^: 1347. See Figures S100–S102.

##### Cbi-Pro-Ex40 (**20**)

**20** was prepared according to the general procedure described above; combining **4** (3.0 mg, 0.0027 mmol) with CuI (3.6 mg, 0.019 mmol), TBTA (7.0 mg, 0.013 mmol), and Ex40 (3.0 mg 0.0007 mmol) gave the target compound, which was purified using RP-HPLC method A2 to produce **20** as a red solid to 97% purity. *t*_R_: 11.8 min; ESI-MS-expected *m*/*z* = 5456, observed *m*/*z* = [M^+^–H_2_O + 2H^+^]^+3^: 1813, [M^+^–H_2_O + 3H^+^]^+4^: 1360, [M^+^–H_2_O + 4H^+^]^+5^: 1088, [M^+^–H_2_O + 5H^+^]^+6^: 907, [M^+^–H_2_O + 6H^+^]^+7^: 777. See Figures S26–S28.

##### Cbi-But-Ex40 (**21**)

**21** was prepared according to the general procedure described above; combining **5** (3.4 mg, 0.0030 mmol) with CuI (4.0 mg, 0.021 mmol), TBTA (6.5 mg, 0.012 mmol), and Ex40 (3.3 mg 0.0007 mmol) gave the target compound, which was purified using RP-HPLC method A2 to produce **21** as a red solid to 97% purity. *t*_R_: 11.7 min; ESI-MS-expected *m*/*z* = 5469, observed *m*/*z* = [M^+^–H_2_O + 2H^+^ + CH_3_CN]^+3^: 1859, [M^+^–H_2_O + 2H^+^]^+3^: 1819, [M^+^–H_2_O + 3H^+^ + CH_3_OH]^+4^: 1395, [M^+^–H_2_O + 3H^+^]^+4^: 1364, [M^+^–H_2_O + 4H^+^]^+5^: 1091. See Figures S37–S39.

##### Cbi-Pent-Ex40 (**22**)

**22** was prepared according to the general procedure described above; combining **6** (4.4 mg, 0.00388 mmol) with CuI (3.2 mg, 0.017 mmol), TBTA (6.5 mg, 0.012 mmol), and Ex40 (4.3 mg 0.0010 mmol) gave the target compound, which was purified using RP-HPLC method A2 to produce **22** as a red solid to 97% purity. *t*_R_: 11.8 min; ESI-MS-expected *m*/*z* = 5483, observed *m*/*z* = [M^+^–H_2_O + 2H^+^]^+3^: 1822, [M^+^–H_2_O + 3H^+^]^+4^: 1367, [M^+^–H_2_O + 5H^+^]^+6^: 912, [M^+^–H_2_O + 6H^+^]^+7^: 781. See Figures S48–S50.

##### Cbi-Hex-Ex40 (**23**)

**23** was prepared according to the general procedure described above; combining **7** (2.1 mg, 0.0018 mmol) with CuI (3.6 mg, 0.019 mmol), TBTA (7.1 mg, 0.013 mmol), and Ex40 (2.6 mg 0.0006 mmol) gave the target compound, which was purified using RP-HPLC method A2 to produce **23** as a red solid to 97% purity. *t*_R_: 11.8 min; ESI-MS-expected *m*/*z* = 5497, observed *m*/*z* = [M^+^–H_2_O + 2H^+^]^+3^: 1827, [M^+^–H_2_O + 3H^+^]^+4^: 1371, [M^+^–H_2_O + 5H^+^]^+6^: 914, [M^+^–H_2_O + 6H^+^]^+7^: 784, [M^+^–H_2_O + 7H^+^]^+8^: 685. See Figures S59–S61.

##### Cbi-PEG2-Ex40 (**24**)

**24** was prepared according to the general procedure described above; combining **8** (2.0 mg, 0.0017 mmol) with CuI (4.1 mg, 0.022 mmol), TBTA (7.4 mg, 0.014 mmol), and Ex40 (4.0 mg 0.0009 mmol) gave the target compound, which was purified using RP-HPLC method A2 to produce **24** as a red solid to 97% purity. *t*_R_: 11.6 min; ESI-MS-expected *m*/*z* = 5544, observed *m*/*z* = [M^+^–H_2_O + 2H^+^ + CH_3_CN]^+3^: 1884, [M^+^–H_2_O + 3H^+^ + CH_3_OH]^+4^: 1414, [M^+^–H_2_O + 3H^+^]^+4^: 1382. See Figures S70–S72.

##### Cbi-PEG4-Ex40 (**25**)

**25** was prepared according to the general procedure described above; combining **9** (3.0 mg, 0.0023 mmol) with CuI (3.3 mg, 0.017 mmol), TBTA (6.8 mg, 0.013 mmol), and Ex40 (4.1 mg 0.0009 mmol) gave the target compound, which was purified using RP-HPLC method A2 to produce **25** as a red solid to 97% purity. *t*_R_: 12.0 min; ESI-MS-expected *m*/*z* = 5632, observed *m*/*z* = [M^+^–H_2_O + 2H^+^ + CH_3_CN]^+3^: 1914, [M^+^–H_2_O + 3H^+^ + CH_3_OH]^+4^: 1436. See Figures S81–S83.

##### Cbi-4EPMA-Ex40 (**26**)

**26** was prepared according to the general procedure described above; combining **10** (2.1 mg, 0.0018 mmol) with CuI (3.0 mg, 0.016 mmol), TBTA (6.7 mg, 0.013 mmol), and Ex40 (2.3 mg 0.0005 mmol) gave the target compound, which was purified using RP-HPLC method A2 to produce **26** as a red solid to 98% purity. *t*_R_: 11.9 min; ESI-MS-expected *m*/*z* = 5531, observed *m*/*z* = [M^+^–H_2_O + 3H^+^ + CH_3_OH]^+4^: 1411, [M^+^–H_2_O + 3H^+^]^+4^: 1379, [M^+^–H_2_O + 4H^+^]^+5^: 1104. See Figures S92–S94.

##### Cbi-3EPMA-Ex4 (**27**)

**27** was prepared according to the general procedure described above; combining **11** (3.0 mg, 0.0025 mmol) with CuI (3.4 mg, 0.018 mmol), TBTA (7.0 mg, 0.013 mmol), and Ex40 (2.0 mg 0.0005 mmol) gave the target compound, which was purified using RP-HPLC method A2 to produce **27** as a red solid to 97% purity. *t*_R_: 11.9 min; ESI-MS-expected *m*/*z* = 5531, observed *m*/*z* = [M^+^–H_2_O + 2H^+^ + CH_3_CN]^+3^: 1880, [M^+^–H_2_O + 2H^+^]^+3^: 1838, [M^+^–H_2_O + 3H^+^ + CH_3_OH]^+4^: 1410, [M^+^–H_2_O + 3H^+^]^+4^: 1379, [M^+^–H_2_O + 4H^+^]^+5^: 1103. See Figures S103–S105.

#### Circular Dichroism (CD) Measurements

All CD spectra were recorded in three independent runs in 100 μL of H_2_O with a final concentration of 40 μM using a Jasco J-715 circular dichroism spectropolarimeter at 25 °C in a 0.1 cm path-length cuvette. The spectra were recorded from 250 to 200 nm and averaged over 6 scans with a resolution of 1.0 nm, a band width of 1.0 nm, and a response time of 4 s. The mean residue ellipticity was plotted versus wavelength using Prism GraphPad 8.

#### Statement on Biological Evaluations

All Cbi-peptide conjugates were at or above 97% purity as confirmed by RP-HPLC (see the figures in the Supporting Information) prior to use in *in vitro*, in islet, or in *in vivo* experiments.

All *in vitro* and islet experiments were conducted at least in triplicate independent runs, aside from the binding experiments, which were in duplicate independent runs. *In vivo* data was analyzed with repeated-measurements one-way or two-way ANOVA followed by Tukey’s posthoc test.

#### Agonism (EC_50_) at the Human GLP-1R for **12**–**27**

Agonism at the GLP-1R was monitored utilizing HEK-293 cells stably transfected with both the human GLP-1R and H188 cAMP FRET reporter cultured in DMEM with 15% FBS, 1% pen/strep, and 250 μg/mL geneticin/G-418. Cells were placed in a 96-well plate in suspension at 200 μL of standard extracellular saline with 11 mM glucose and 0.1% BSA at ∼60,000 cells/well. Peptides and conjugates were injected to each well at 5× the required concentration. Agonism was determined through an increase in the 485/535 nm FRET ratio, indicative of an increase in the cAMP level through binding to the H188 cAMP FRET reporter.

#### Agonism (EC_50_) at the Rat GLP-1R

Ex4, Ex40, and **22** were screened as previously described.^69^

#### Competitive Binding Assay (IC_50_) at Human GLP-1R for **12**–**27**

IC_50_ values were measured in CHO-K1 cells at the human GLP-1R by Euroscreen Fast (Gosselies, Belgium) using their proprietary Taglite fluorescence competitive binding assay (Cat No. FAST0154B). The agonist tracer was GLP-1red at 4 nM with reference competitor nEx-4. Conjugates were assayed in duplicate independent runs at nine concentrations per run ranging from 1 pM to 1 μM (Figure S110).

#### Statement on Animal Experiments

All procedures conducted in rats were approved by the Institutional Care and Use Committee of the University of Washington and conducted in compliance with the U.S. federal law and institutional guidelines, which are congruent with the NIH guide for the Care and Use of Laboratory Animals.

All procedures conducted in shrews were approved by the Institutional Care and Use Committee of the University of Pennsylvania and conducted in compliance with the U.S. federal law and institutional guidelines, which are congruent with the NIH guide for the Care and Use of Laboratory Animals.

#### Rat Islet Isolation and Culture

Islets were harvested from Sprague–Dawley rats (approximately 250 g; Envigo/Harlan) anesthetized by intraperitoneal injection of pentobarbital sodium (150 mg/kg rat). Islets were prepared and purified as described.^70,71^ Islets were then cultured for 18 h at 37 °C, gassed with 5% CO_2_ in an incubator in an RPMI medium supplemented with 10% heat-inactivated fetal bovine serum (Invitrogen).

#### Static Measurement of ISR

ISR was determined statically as described previously.^72^ Briefly, islets were handpicked into a Petri dish containing KRB buffer supplemented with 0.1% bovine serum albumin and 3 mM glucose and incubated at 37 °C gassed with 5% CO_2_ for 60 min. Subsequently, islets were placed into 96-well plates containing desired amounts of glucose and agents as indicated (Figure 5) and incubated for an additional 60 min. At the end of this period, the supernatant was assayed for insulin by RIA.

#### *In Vivo* Study Design in Shrews

Adult male shrews (*n* = 32, *Suncus murinus*) weighing ∼60 g were bred at the University of Pennsylvania. These animals generated in our lab were originally derived from a colony maintained at the University of Pittsburgh Cancer Institute (a Taiwanese strain derived from stock supplied by the Chinese University of Hong Kong). Shrews were single-housed in plastic cages (37.3 × 23.4 × 14 cm, Innovive), fed *ad libitum* with a mixture of feline (75%, Laboratory Feline Diet 5003, Lab Diet) and ferret food (25%, High Density Ferret Diet 5LI4, Lab Diet), and had *ad libitum* access to tap water except where noted. All animals were housed under a 12/12 h light/dark cycle in a temperature- and humidity-controlled environment. Shrews were habituated to single housing in their home cages and injected IP at least 1 week prior to experimentation. All experimental injections in shrews were separated by at least 72 h. Behavioral experiments were conducted in a within-subjects, Latin square design.

#### Effects of Ex4, **1**, and **22** on Energy Balance in Shrews

We first evaluated in shrews the effects on food intake and body weight of doses of nEx4, **1**, and **22**. Food intake was evaluated using our custom-made feedometers, consisting of a standard plexiglass rodent housing cage (29 × 19 × 12.7 cm) with mounted food hoppers resting on a plexiglass cup (to account for spillage). Shrews had *ad libitum* access to powdered food through a circular (3 cm diameter) hole in the cage. Food was removed 1 h prior to the dark onset. Shortly before the dark onset, shrews (*n* = 10) received IP injection of Ex4 (5 nmol/kg), **1** (5 nmol/kg), **22** (5 nmol/kg), or vehicle (1 mL/100 g BW sterile saline). Food intake was manually measured at 6, 24, and 48 h post injection. BW was measured at 0, 24, and 48 h. Treatments occurred in a within-subject, counter-balanced design and were at least 3 days apart.

#### Effects of Ex4, **1**, and **22** on Glucoregulation Following an IPGTT in Shrews

The protocol for performing an IPGTT in shrews was similar to that previously described.^18,51^ Briefly; 3 h before the dark onset, shrews (*n* = 12) were food- and water-deprived. Two hours later, baseline BG levels were determined from a small drop of tail blood and measured using a standard glucometer. nEx4 (5 nmol/kg), **1** (5 nmol/kg), **22** (5 nmol/kg), or vehicle (1 mL/100 g BW sterile saline) were then administered IP. BG was measured 30 min later (*t* = 0 min), and then each shrew received an IP bolus of glucose (2 g/kg). Subsequent BG readings were taken at 20, 40, 60, and 120 min after glucose injection. After the final BG reading, food and water were returned. IPGTT studies were carried out in a within-subject, counter-balanced design and separated by at least 72 h.

#### Effects of Ex4, **1**, and **22** on Emesis in Shrews

Shrews (*n* = 10) were habituated to IP injections and to clear plastic observation chambers (23.5 × 15.25 × 17.8 cm) for four consecutive days prior to experimentation. The animals were injected IP with nEx4, **1**, **22** (all at 5 nmol/kg), or vehicle, placed in their respective emetic cages, and then video-recorded (Vixia HF-R62, Canon) for 120 min. All recordings started within 2 min after drug administration. After 120 min, the animals were returned to their cages. Treatments were separated by 72 h. Analysis of emetic episodes was performed by an observer blinded to treatment groups. Emetic episodes were characterized by strong rhythmic abdominal contractions associated with either oral expulsion from the gastrointestinal tract (vomiting) or without the passage of materials (retching). Latency to the first emetic episode, the total number of emetic episodes, and the number of emetic episodes per minute were recorded.

## Supporting Information Available

RP-HPLC purity traces, electrospray ionization mass spectra, ^1^H and ^13^C nuclear magnetic resonance spectra, electronic absorption spectra, and in vitro dose response curves (PDF)

## Notes

^¥^ M.R.H., B.C.D.J., and R.P.D. are senior authors.

## ABBREVIATIONS

3EPMA: (3-ethynylphenyl)methanamine
4EPMA: (4-ethynylphenyl)methanamine
AUC: area under the curve
BG: blood glucose
BSA: bovine serum albumin
cAMP: cyclic adenosine monophosphate
Cbi: dicyanocobinamide
CD: circular dichroism
CDT: 1,1′-carbonyl-di-(1,2,4-triazole)
CNS: central nervous system
DMEM: Dulbecco’s modified Eagle medium
DMF: dimethylformamide
EAS: electronic absorption spectra
EC_50_: half-maximal effective concentration
EPAC: exchange protein directly activated by cAMP
Ex4: exendin-4 with K12 azido modification
Ex40: exendin-4 with an azido-lysine added as residue 40
FBS: fetal bovine serum
FRET: Forster resonance energy transfer
GLP-1R: glucagon like peptide-1 receptor
GPCR: G-protein coupled receptor
GSIS: glucose-stimulated insulin secretion
HEK: human embryonic kidney cells
IC_50_: half-maximal inhibitory concentration
IPGTT: intraperitoneal glucose tolerance test
ISR: insulin secretion rate
nEx4: native Exendin-4
NMP: 1-methyl-2-pyrrolidinone
NMR: nuclear magnetic resonance
PNS: peripheral nervous system
RIA: radioimmunoassay
RP-HPLC: reversed-phase high-performance liquid chromatography
SD: Sprague–Dawley rat
SEM: standard error of the mean
SES: standard extracellular saline
T2D: type 2 diabetes mellitus
BTA: tris(benzyltriazolylmethyl)amine
TEA: triethylamine
t_R_: retention time

## References

[ref1] Chen L.; Magliano D. J.; Zimmet P. Z. The worldwide epidemiology of type 2 diabetes mellitus--present and future perspectives. Nat. Rev. Endocrinol. 2012, 8, 228–236. 10.1038/nrendo.2011.183. 22064493

[ref2] Flegal K. M.; Carroll M. D.; Ogden C. L.; Curtin L. R. Prevalence and trends in obesity among US adults, 1999-2008. JAMA 2010, 303, 235–241. 10.1001/jama.2009.2014. 20071471

[ref3] Sherwin R.; Jastreboff A. M. Year in diabetes 2012: The diabetes tsunami. J. Clin. Endocrinol. Metab. 2012, 97, 4293–4301. 10.1210/jc.2012-3487. 23185035 PMC3513534

[ref4] Franks P. W.; McCarthy M. I. Exposing the exposures responsible for type 2 diabetes and obesity. Science 2016, 354, 69–73. 10.1126/science.aaf5094. 27846494

[ref5] Upadhyay J.; Polyzos S. A.; Perakakis N.; Thakkar B.; Paschou S. A.; Katsiki N.; Underwood P.; Park K. H.; Seufert J.; Kang E. S.; Sternthal E.; Karagiannis A.; Mantzoros C. S. Pharmacotherapy of type 2 diabetes: An update. Metabolism. 2018, 78, 13–42. 10.1016/j.metabol.2017.08.010. 28920861

[ref6] Sadry S. A.; Drucker D. J. Emerging combinatorial hormone therapies for the treatment of obesity and T2DM. Nat. Rev. Endocrinol. 2013, 9, 425–433. 10.1038/nrendo.2013.47. 23478327

[ref7] Drucker D. J.; Sherman S. I.; Bergenstal R. M.; Buse J. B. The safety of incretin-based therapies--review of the scientific evidence. J. Clin. Endocrinol. Metab. 2011, 96, 2027–2031. 10.1210/jc.2011-0599. 21734003

[ref8] Hayes M. R.; Mietlicki-Baase E. G.; Kanoski S. E.; De Jonghe B. C. Incretins and amylin: neuroendocrine communication between the gut, pancreas, and brain in control of food intake and blood glucose. Annu. Rev. Nutr. 2014, 34, 237–260. 10.1146/annurev-nutr-071812-161201. 24819325 PMC4458367

[ref9] Holst J. J. The physiology of glucagon-like peptide 1. Physiol. Rev. 2007, 87, 1409–1439. 10.1152/physrev.00034.2006. 17928588

[ref10] Baggio L. L.; Drucker D. J. Biology of incretins: GLP-1 and GIP. Gastroenterology 2007, 132, 2131–2157. 10.1053/j.gastro.2007.03.054. 17498508

[ref11] Lovshin J. A.; Drucker D. J. Incretin-based therapies for type 2 diabetes mellitus. Nat. Rev. Endocrinol. 2009, 5, 262–269. 10.1038/nrendo.2009.48. 19444259

[ref12] Hayes M. R.; Schmidt H. D. GLP-1 influences food and drug reward. Curr. Opin. Behav. Sci. 2016, 9, 66–70. 10.1016/j.cobeha.2016.02.005. 27066524 PMC4822543

[ref13] Kanoski S. E.; Hayes M. R.; Skibicka K. P. Glp-1 and weight loss: Unraveling the diverse neural circuitry. Am. J. Physiol. Regul. Integr. Comp. Physiol. 2016, 310, R885–R895. 10.1152/ajpregu.00520.2015. 27030669 PMC4888559

[ref14] Knudsen L. B.; Bielsen P. F.; Huusfeldt P. O.; Johansen N. L.; Madsen K.; Pederson F. Z.; Thogersen H.; Wilken M.; Agerso H. Potent Derivatives of glucagon-like peptide-1 with pharmacokinetic properties suitable for once daily administration. J. Med. Chem. 2000, 43, 1664–1669. 10.1021/jm9909645. 10794683

[ref15] Madsen K.; Knudsen L. B.; Agresoe H.; Nielsen P. F.; Thogersen H.; Wilken M.; Johansen N. L. Structure-activity and protraction relationship of long-acting glucagon-like peptide-1 derivative: importance of fatty acid length, polarity, and bulkiness. J. Med. Chem. 2007, 50, 6126–6132. 10.1021/jm070861j. 17975905

[ref16] Knudsen L. B.; Lau J. The discovery and development of liraglutide and semaglutide. Front. Endocrinol. 2019, 10, 155 10.3389/fendo.2019.00155. PMC6474072 31031702

[ref17] O’Niel P. M.; Birkenfeld A. L.; McGowan B.; Mosenzon O.; Pederson S. D.; Wharton S.; Carson C. G.; Jepsen C. H.; Kabisch M.; Wilding J. P. H. Efficacy and safery of semaglutide compared with liraglutide and placebo for weightloss in patients with obesity: a randomised, double-blind, placebo and acive controlled, dose-ranging, phase 2 trial. Lancet 2018, 392, 637–649. 10.1016/S0140-6736(18)31773-2. 30122305

[ref18] Lau J.; Bloch P.; Schaffer L.; Pettersson I.; Spetzler J.; Kofoed J.; Madsen K.; Knudsen L. B.; McGuire J.; Steensgaard D. B.; Strauss H. M.; Gram D. X.; Knudsen S. M.; Bielsen F. S.; Thygesen P.; Reedtz-Runge S.; Kruse T. Discovery of the once-weekly glucagon-like peptide-1 (GLP-1) analog semaglutide. J. Med. Chem. 2015, 58, 7370–7380. 10.1021/acs.jmedchem.5b00726. 26308095

[ref19] Bergenstal R. M.; Wysham C.; Macconell L.; Malloy J.; Walsh B.; Yan P.; Wilhelm K.; Malone J.; Porter L. E. Efficacy and safety of exenatide once weekly versus sitagliptin or pioglitazone as an adjunct to metformin for treatment of type 2 diabetes (DURATION-2): a randomised trial. Lancet 2010, 376, 431–439. 10.1016/S0140-6736(10)60590-9. 20580422

[ref20] Buse J. B.; Henry R. R.; Han J.; Kim D. D.; Fineman M. S.; Baron A. D. Effects of exenatide (exendin-4) on glycemic control over 30 weeks in sulfonylurea-treated patients with type 2 diabetes. Diabetes Care 2004, 27, 2628–2635. 10.2337/diacare.27.11.2628. 15504997

[ref21] DeFronzo R. A.; Ratner R. E.; Han J.; Kim D. D.; Fineman M. S.; Baron A. D. Effects of exenatide (exendin-4) on glycemic control and weight over 30 weeks in metformin-treated patients with type 2 diabetes. Diabetes Care 2005, 28, 1092–1100. 10.2337/diacare.28.5.1092. 15855572

[ref22] Kendall D. M.; Riddle M. C.; Rosenstock J.; Zhuang D.; Kim D. D.; Fineman M. S.; Baron A. D. Effects of exenatide (exendin-4) on glycemic control over 30 weeks in patients with type 2 diabetes treated with metformin and a sulfonylurea. Diabetes Care 2005, 28, 1083–1091. 10.2337/diacare.28.5.1083. 15855571

[ref23] John L. E.; Kane M. P.; Busch R. S.; Hamilton R. A. Expanded use of exenatide in the management of type 2 diabetes. Diabetes Spectrum. 2007, 20, 59–63. 10.2337/diaspect.20.1.59.

[ref24] Weinstock R. S.; Guerci B.; Umpierrez G.; Nauck M. A.; Skrivanek Z.; Milicevic Z. Safety and efficacy of once-weekly dulaglutide versus sitagliptin after 2 years in metformin-treated patients with type 2 diabetes (AWARD-5): a randomized, phase III study. Diabetes, Obes. Metab. 2015, 17, 849–858. 10.1111/dom.12479. 25912221 PMC5008205

[ref25] Wang T.; Gou Z.; Wang F.; Ma M.; Zhai S. D. Comparison of GLP-1 analogues versus sitagliptin in the management of type 2 diabetes: systematic review and meta-analysis of head-to-head studies. PLoS One 2014, 9, e103798 10.1371/journal.pone.0103798. 25089625 PMC4121242

[ref26] Kanoski S. E.; Fortin S. M.; Arnold M.; Grill H. J.; Hayes M. R. Peripheral and central GLP-1 receptor populations mediate the anorectic effects of peripherally administered GLP-1 receptor agonists, liraglutide and exendin-4. Endocrinology 2011, 152, 3103–3112. 10.1210/en.2011-0174. 21693680 PMC3138234

[ref27] Sisley S.; Gutierrez-Aguilar R.; Scott M.; D’Alessio D. A.; Sandoval D. A.; Seeley R. J. Neuronal GLP1R mediates liraglutide’s anorectic but not glucose-lowering effect. J. Clin. Invest. 2014, 124, 2456–2463. 10.1172/JCI72434. 24762441 PMC4038572

[ref28] Secher A.; Jelsing J.; Baquero A. F.; Hecksher-Sorensen J.; Cowley M. A.; Dalboge L. S.; Hansen G.; Grove K. L.; Pyke C.; Raun K.; Schaffer L.; Tang-Christensen M.; Verma S.; Witgen B. M.; Vrang N.; Knudsen L. B. The arcuate nucleus mediates GLP-1 receptor agonist liraglutide-dependent weight loss. J. Clin. Invest. 2014, 124, 4473–4488. 10.1172/JCI75276. 25202980 PMC4215190

[ref29] Kanoski S. E.; Rupprecht L. E.; Fortin S. M.; De Jonghe B. C.; Hayes M. R. The role of nausea in food intake and body weight suppression by peripheral GLP-1 receptor agonists, exendin-4 and liraglutide. Neuropharmacology 2012, 62, 1916–1927. 10.1016/j.neuropharm.2011.12.022. 22227019 PMC4183930

[ref30] Moheet A.; Moran A. CF-related diabetes: Containing the metabolic miscreant of cystic fibrosis. Pediatr. Pulmonol. 2017, 52, S37–S43. 10.1002/ppul.23762. 28714601

[ref31] Husain N. E.; Noor S.; Elmadhoun W.; Almobarak A.; Awadalla H.; Woodward C.; Mital D.; Ahmed M. Diabetes, metabolic syndrome and dyslipidemia in people living with HIV in Africa: re-emerging challenges not to be forgotten. HIV AIDS 2017, Volume 9, 193–202. 10.2147/HIV.S137974. PMC5685138 29184449

[ref32] Gallo M.; Muscogiuri G.; Felicetti F.; Faggiano A.; Trimarchi F.; Arvat E.; Vigneri R.; Colao A. Adverse glycaemic effects of cancer therapy: indications for a rational approach to cancer patients with diabetes. Metabolism. 2018, 78, 141–154. 10.1016/j.metabol.2017.09.013. 28993227

[ref33] Ho T.-W.; Huang C.-T.; Ruan S.-Y.; Tsai Y.-J.; Lai F.; Yu C.-J. Diabetes mellitus in patients with chronic obstructive pulmonary disease-The impact on mortality. PLoS One 2017, 12, e0175794 10.1371/journal.pone.0175794. 28410410 PMC5391945

[ref34] Honors M. A.; Kinzig K. P. The role of insulin resistance in the development of muscle wasting during cancer cachexia. J. Cachexia Sarcopenia Muscle. 2012, 3, 5–11. 10.1007/s13539-011-0051-5. 22450024 PMC3302982

[ref35] Bonaccorso R. L.; Chepurny O. G.; Becker-Pauly C.; Holz G. G.; Doyle R. P. Enhanced peptide stability against protease digestion induced by intrinsic factor binding of a vitamin B12 conjugate of exendin-4. Mol. Pharmaceutics 2015, 12, 3502–3506. 10.1021/acs.molpharmaceut.5b00390. PMC4660977 26260673

[ref36] Mietlicki-Baase E. G.; Liberini C. G.; Workinger J. L.; Bonaccorso R. L.; Borner T.; Reiner D. J.; Koch-Lasowski K.; McGrath L. E.; Lhamo R.; Stein L. M.; De Jonghe B. C.; Holz G. G.; Roth C. L.; Doyle R. P.; Hayes M. R. A vitamin B12 conjugate of exendin-4 improves glucose tolerance without associated nausea or hypophagia in rodents. Diabetes, Obes. Metab. 2018, 20, 1223–1234. 10.1111/dom.13222. 29327400 PMC5899935

[ref37] Borner T.; Shaulson E. D.; Tinsley I. C.; Stein L. M.; Horn C. C.; Hayes M. R.; Doyle R. P.; De Jonghe B. C. A second-generation glucagon-like peptide-1 receptor agonist mitigates vomiting and anorexia while retaining glucoregulatory potency in lean diabetic and emetic and mammalian models. Diabetes, Obes. Metab. 2020, 22, 1729–1741. 10.1111/dom.14089. 32410372 PMC7927944

[ref38] Henry K. E.; Elfers C. T.; Burke R. M.; Chepurny O. G.; Holz G. G.; Blevins J. E.; Roth C. L.; Doyle R. P. Vitamin B12 conjugation of peptide-YY(3-36) decreases food intake compared to native peptide-YY(3-36) upon subcutaneous administration in male rats. Endocrinology 2015, 156, 1739–1749. 10.1210/en.2014-1825. 25658456 PMC4398759

[ref39] Borner T.; Workinger J. L.; Tinsley I. C.; Fortin S. M.; Stein L. M.; Chepurny O. G.; Holz G. G.; Wierzba A. J.; Gryko D.; Nexø E.; Shaulson E. D.; Bamezai A.; Rodriguez Da Silva V. A.; De Jonghe B. C.; Hayes M. R.; Doyle R. P. Corrination of a GLP-1 receptor agonist for glycemic control without emesis. Cell Rep. 2020, 31, 107768 10.1016/j.celrep.2020.107768. 32553160 PMC7376604

[ref40] Workinger J. L.; Kuda-Wedagedara A. N. W.; Julin M. M.; White J. M.; Nexo E.; Viola N. T.; Doyle R. P. Systemically administered plant recombinant holo-intrinsic factor targets the liver and is not affected by endogenous B12 levels. Sci. Rep. 2019, 9, 12269 10.1038/s41598-019-48555-w. 31439908 PMC6706418

[ref41] De Jonghe B. C.; Lawler M. P.; Horn C. C.; Tordoff M. G. Pica as an adaptive response: kaolin consumption helps rats recover from chemotherapy-induced illness. Physiol. Behav. 2009, 97, 87–90. 10.1016/j.physbeh.2009.02.009. 19419663 PMC2680461

[ref42] Ueno S.; Matsuki N.; Saito H. Suncus murinus: a new experimental model in emesis research. Life Sci. 1987, 41, 513–518. 10.1016/0024-3205(87)90229-3. 3600192

[ref43] Chan S. W.; Lin G.; Yew D. T. W.; Yeung C. K.; Rudd J. A. Separation of emetic and anorexic responses of exendin-4, a GLP-1 receptor agonist in Suncus murinus (house musk shrew). Neuropharmacology 2013, 70, 141–147. 10.1016/j.neuropharm.2013.01.013. 23357334

[ref44] Chan S. W.; Lin G.; Yew D. T. W.; Rudd J. A. A physiological role of glucagon-like peptide-1 receptors in the central nervous system of Suncus murinus (house musk shrew). Eur. J. Pharmacol. 2011, 668, 340–346. 10.1016/j.ejphar.2011.06.036. 21756894

[ref45] Anantharam P.; Whitley E. M.; Mahama B.; Kim D. S.; Sarkar S.; Santana C.; Chan A.; Kanthasamy A. G.; Kanthasamy A.; Boss G. R.; Rumbeiha W. Cobinamide is effective for treatment of hydrogen sulfide-induced neurological sequelae in a mouse model. Anna. N.Y. Acad. Sci. 2017, 1408, 61–78. 10.1111/nyas.13559. PMC5734662 29239480

[ref46] Hendry-Hofer T. B.; Ng P. C.; McGrath A. M.; Mukai D.; Brenner M.; Mahon S.; Maddry J. K.; Boss G. R.; Bebarta V. S. Intramuscular aminotetrazole cobinamide as a treatment for inhaled hydrogen sulfide poisoning in a large swine model. Ann. N.Y. Acad. Sci. 2020, 1479, 159–167. 10.1111/nyas.14339. 32233102

[ref47] Ma J.; Dasgupta P. K.; Zelder F. H.; Boss G. R. Cobinamide chemistries for photometric cyanide determination. A merging zone liquid core waveguide cyanide analyzer using cyanoaquacobinamide. Anal. Chim. Acta 2012, 736, 78–84. 10.1016/j.aca.2012.05.028. 22769008 PMC3392611

[ref48] Männel-Croisé C.; Probst B.; Zelder F. A straightforward method for the colorimetric detection of endogenous biological cyanide. Anal. Chem. 2009, 81, 9493–9498. 10.1021/ac901977u. 19842647

[ref49] Brenner M.; Mahon S. B.; Lee J.; Kim J.; Mukai D.; Goodman S.; Kreuter K. A.; Ahdout R.; Mohammad O.; Sharma V. S.; Blackledge W.; Boss G. R. Comparison of cobinamide to hydroxocobalamin in reversing cyanide physiologic effects in rabbits using diffuse optical spectroscopy monitoring. J. Biomed. Opt. 2010, 15, 017001 10.1117/1.3290816. 20210475 PMC2816993

[ref50] Broderick K. E.; Potluri P.; Zhuang S.; Scheffler I. E.; Sharma V. S.; Pilz R. B.; Boss G. R. Cyanide detoxification by the cobalamin precursor cobinamide. Exp. Biol. Med. 2016, 231, 641–649. 10.1177/153537020623100519. 16636313

[ref51] Chan A.; Jiang J.; Fridman A.; Guo L. T.; Shelton G. D.; Liu M.-T.; Green C.; Haushalter K. J.; Patel H. H.; Lee J.; Yoon D.; Burney T.; Mukai D.; Mahon S. B.; Brenner M.; Pilz R. B.; Boss G. R. Nitrocobinamide, a new cyanide antidote that can be administered by intramuscular injection. J. Med. Chem. 2015, 58, 1750–1759. 10.1021/jm501565k. 25650735 PMC4494098

[ref52] Chan A.; Balasubramanian M.; Blackledge W.; Mohammad O. M.; Alvarez L.; Boss G. R.; Bigby T. D. Cobinamide is superior to other treatments in a mouse model of cyanide poisoning. Clin. Toxicol. 2010, 48, 709–717. 10.3109/15563650.2010.505197. PMC3119202 20704457

[ref53] Lee J.; Mahon S. B.; Mukai D.; Burney T.; Katebian B. S.; Chan A.; Bebarta V. S.; Yoon D.; Boss G. R.; Brenner M. The vitamin B12 analog cobinamide is an effective antidote for oral cyanide poisoning. J. Med. Toxicol. 2016, 12, 370–379. 10.1007/s13181-016-0566-4. 27631586 PMC5135677

[ref54] Greenawald L. A.; Snyder J. L.; Fry N. L.; Sailor M. J.; Boss G. R.; Finklea H. O.; Bell S. Development of a cobinamide-based end-of-service-life indicator for detection of hydrogen cyanide gas. Sens. Actuators B Chem. 2015, 221, 379–385. 10.1016/j.snb.2015.06.085. 26213448 PMC4511729

[ref55] Wierzba A. J.; Maximova K.; Wincenciuk A.; Równicki M.; Wojciechowska M.; Nexø E.; Trylska J.; Gryko D. Does a conjugation site affect transport of vitamin B12-peptide nucleic acid conjugates into bacterial cells. Chem. – Eur. J. 2018, 24, 18772–18778. 10.1002/chem.201804304. 30286265

[ref56] Evers A.; Haack T.; Lorenz M.; Bossart M.; Elvert R.; Henkel B.; Stengelin S.; Kurz M.; Glien M.; Dudda A.; Lorenz K.; Kadereit D.; Wagner M. Design of novel exendin-based dual glucagon-like peptide 1 (GLP-1)/glucagon receptor agonists. J. Med. Chem. 2017, 60, 4293–4303. 10.1021/acs.jmedchem.7b00174. 28448133

[ref57] Dai S.; Liu S.; Li C.; Zhou Z.; Wu Z. Site-selective modification of exendin 4 with variable molecular weight dextrans by oxime-ligation chemistry for improving type 2 diabetic treatment. Carbohydr. Polym. 2020, 249, 116864 10.1016/j.carbpol.2020.116864. 32933691

[ref58] Lee J. G.; Ryu J. H.; Kim S. M.; Park M. Y.; Kim S. H.; Shin Y. G.; Sohn J. W.; Kim H. H.; Park Z. Y.; Seong J. Y.; Kim J. I. Replacement of the C-terminal Trp-cage of exendin-4 with a fatty acid improves therapeutic utility. Biochem. Pharmacol. 2018, 151, 59–68. 10.1016/j.bcp.2018.03.004. 29522713

[ref59] Ó Proinsias K.; Karczewski M.; Zieleniewska A.; Gryko D. Microwave-assisted cobinamide synthesis. J. Org. Chem. 2014, 79, 7752–7757. 10.1021/jo501364b. 25058239

[ref60] Zhou K.; Zelder F. Identification of diastereomeric cyano-aqua cobinamides with a backbone-modified vitamin B12 derivative and with ^1^H NMR spectroscopy. Eur. J. Inorg. Chem. 2011, 53–57. 10.1002/ejic.201001146.

[ref61] Berg R.; Straub B. F. Advancements in the mechanistic understanding of the copper-catalyzed azide-alkyne cycloaddition. Beilstein J. Org. Chem. 2013, 9, 2715–2750. 10.3762/bjoc.9.308. 24367437 PMC3869285

[ref62] Tibaduiza E. C.; Chen C.; Beinborn M. A Small molecule ligand of the glucagon-like peptide 1 receptor targets its amino-terminal hormone binding domain. J. Biol. Chem. 2001, 276, 37787–37793. 10.1074/jbc.M106692200. 11498540

[ref63] Klarenbeek J.; Goedhart J.; van Batenburg A.; Groenewald D.; Jalink K. Fourth generation EPAC-based FRET sensors for cAMP feature exceptional brightness, photostability and dynamic range: characterization of dedicated sensors for FLIM, for ratiometry and with high affinity. PLoS One 2015, 10, e0122513 10.1371/journal.pone.0122513. 25875503 PMC4397040

[ref64] Khaodhiar L.; McCowen K. C.; Blackburn G. L. Obesity and its comorbid conditions. Clin. Cornerstone. 1999, 2, 17–31. 10.1016/S1098-3597(99)90002-9. 10696282

[ref65] Mroz P. A.; Perez-Tilve D.; Mayer J. P.; DiMarchi R. D. Stereochemical inversion as a route to improved biophysical properties of therapeutic peptides exemplified by glucagon. Commun. Chem. 2019, 2, 2 10.1038/s42004-018-0100-5.

[ref66] Chabenne J. R.; Mroz P. A.; Mayer J. P.; DiMarchi R. D. Structural refinement of glucagon for therapeutic use. J. Med. Chem. 2020, 63, 3447–3460. 10.1021/acs.jmedchem.9b01493. 31774682

[ref67] Chabenne J. R.; DiMarchi M. A.; Gelfanov V. M.; DiMarchi R. D. Optimization of the native glucagon sequence for medicinal purposes. J. Diabetes Sci. Technol. 2010, 4, 1322–1331. 10.1177/193229681000400605. 21129326 PMC3005041

[ref68] Chen W.; Lisowski M.; Khalil G.; Sweet I. R.; Shen A. Q. Microencapsulated 3-Dimensional sensor for the measurement of oxygen in single isolated pancreatic islets. PLoS One 2012, 7, e33070 10.1371/journal.pone.0033070. 22479359 PMC3315556

[ref69] Milliken B. T.; Chepurny O. G.; Doyle R. P.; Holz G. G. FRET reporter assays for cAMP and calcium in a 96-well format using genetically encoded biosensors expressed in living cells. Bio-Protoc. 2020, 10, e3641 10.21769/BioProtoc.3641. 32775537 PMC7409823

[ref70] Sweet I. R.; Cook D. L.; DeJulio E.; Wallen A. R.; Khalil G.; Callis J.; Reems J. Regulation of ATP/ADP in pancreatic islets. Diabetes 2004, 53, 401–409. 10.2337/diabetes.53.2.401. 14747291

[ref71] Matsumoto S.; Shibata S.; Kirchhof N.; Hiraoka K.; Sageshima J.; Zhang X. W.; Gilmore T.; Ansite J.; Zhang H. J.; Sutherland D. E. R.; Hering B. J. Immediate reversal of diabetes in primates following intraportal transplantation of porcine islets purified on a new histidine-lactoioniate-iodixanol gradient. Transplantation. 1999, 67, S220 10.1097/00007890-199904150-00880.

[ref72] Jung S.-R.; Reed B. J.; Sweet I. R. A highly energetic process couples calcium influx through L-type calcium channels to insulin secretion in pancreatic beta-cells. Am. J. Physiol. 2009, 297, E717–E72.7. 10.1152/ajpendo.00282.2009 PMC2739700 19584201

