# Supplementary material for: Synthesis, Optimization, and Biological Evaluation of Corrinated Conjugates of the GLP-1R Agonist Exendin-4
Source: J Med Chem. 2021 Mar 6;64(6):3479–92. doi: 10.1021/acs.jmedchem.1c00185 (PMC8279408; doi:10.1021/acs.jmedchem.1c00185)
Supplement: Supplementary file 1 — jm1c00185_si_001.pdf [file jm1c00185_si_001.pdf]

Supporting Information

Design, Synthesis and Biological Evaluation of Corrinated Conjugates of the GLP-1R Agonist Exendin-4

Ian C. Tinsley[1,†,□], Tito Borner[5,¶,□], MacKenzie L. Swanson[1,†], Oleg G. Chepurny[2,‡], Sarah A. Doebley[5,¶], Varun Kamat[3,§], Ian R. Sweet[3,§], George G. Holz[2,‡], Matthew R. Hayes[4,|,¥], Bart C. De Jonghe[5,¶,¥], and Robert P. Doyle[1,2,†,‡,¥,*]

[†] Department of Chemistry, Syracuse University, 111 College Place, Syracuse, New York 13244, United States
[‡] Department of Medicine, State University of New York, Upstate Medical University, Syracuse, New York 13245, United States
[§] Department of Medicine, University of Washington, Medicine Diabetes Institute, Seattle, Washington 98109, United States
[|] Department of Psychiatry, University of Pennsylvania, Perelman School of Medicine, Philadelphia, PA 19104, United States
[¶] Department of Biobehavioral Health Sciences, University of Pennsylvania, School of Nursing, Philadelphia, Pennsylvania 19104, United States

[□] contributed equally to the work

[¥] senior authors

*corresponding author: rpdoyle@syr.edu

Table of Contents **Page Number**

Figure S1. RP-HPLC trace of commercial vitamin B12 at 6.8 min. 8

Figure S2. ESI-MS of commercial vitamin B12, expected m/z = 1355, observed m/z = $[M+H^+]^{+1}$ 1356. 8

Figure S3. Electronic absorption spectroscopy of commercial vitamin B12 in water. 9

Figure S4. ^{1}H NMR of commercial vitamin B12 (400 MHz, 298K, D_2O). 10

Figure S5. ^{1}H NMR of commercial vitamin B12 (400 MHz, 298K, D_2O) (Aromatic). 11

Figure S6. ^{13}C NMR of commercial vitamin B12 (400 MHz, 298K, D_2O). 12

Figure S7. RP-HPLC trace showing the α- and β-isomer products of **2** at 5.2 and 6.7 min. 13

Figure S8. ESI-MS of **2**, expected m/z = 1042, observed m/z = $[M\text{-}CN]^{+1}$ 1016. 13

Figure S9. Electronic absorption spectroscopy of **2** in water. 14

Figure S10. ^{1}H NMR of **2** (400 MHz, 298K, D_2O). 15

Figure S11. ^{13}C NMR of **2** (400 MHz, 298K, D_2O). 16

Figure S12. RP-HPLC trace showing Ex4 at 11.8 min. 17

Figure S13. ESI-MS of Ex4, expected m/z = 4213, observed m/z = $[M+3H^+]^{+3}$ 1405, $[M+4H^+]^{+4}$ 1054 m/z. 17

Figure S14. *In vitro* dose escalation study of Ex4 showing increase in cAMP levels in 18

GLP-1R stably transfected HEK-293-H188 c20 cells.

Figure S15. RP-HPLC trace showing Ex40 at 11.0 min. **18**

Figure S16. ESI-MS of Ex40, expected m/z = 4341, observed m/z = $[M+3H^+]^{+3}$ 1447, $[M+4H^+]^{+4}$ 1086, $[M+5H^+]^{+5}$ 869 m/z. **19**

Figure S17. *In vitro* dose escalation study of Ex40 showing increase in cAMP levels in GLP-1R stably transfected HEK-293-H188 c20 cells. **19**

Figure S18. RP-HPLC trace showing the α- and β-isomer products of **4** at 8.8 and 9.5 min. **20**

Figure S19. ESI-MS of **4**, expected m/z = 1115, observed m/z = $[M^+-H_2O]^{+1}$ 1096, $[M^+-H_2O+H^+]^{+2}$: 549 m/z. **20**

Figure S20. Electronic absorption spectroscopy of **4** in water. **21**

Figure S21. ^{1}H NMR of **4**. (400 MHz, 298K, D_2O). **22**

Figure S22. ^{1}H NMR of **4**. (400 MHz, 298K, D2O) (Aromatic). Characteristic signals (H19) of β- (6.50) and α- (6.42) aquo-isomers of **4** are observed.[1] **23**

Figure S23. RP-HPLC trace showing product **12** at 11.7 min. **24**

Figure S24. ESI-MS of **12**, expected m/z = 5327, observed m/z = $[M^+-H_2O+2H^+]^{+3}$ 1771, $[M^+-H_2O+3H^+]^{+4}$: 1328 m/z. **24**

Figure S25. *In vitro* dose escalation study of **12** showing increase in cAMP levels in GLP-1R stably transfected HEK-293-H188 c20 cells. **25**

Figure S26. RP-HPLC trace showing product **20** at 11.8 min. **25**

Figure S27. ESI-MS of **20**, expected m/z = 5456, observed m/z = $[M^+-H_2O+2H^+]^{+3}$ 1813, $[M^+-H_2O+3H^+]^{+4}$ 1360, $[M^+-H_2O+4H^+]^{+5}$ 1088, $[M^+-H_2O+5H^+]^{+6}$ 907, $[M^+-H_2O+6H^+]^{+7}$ 777 m/z. **26**

Figure S28. In vitro dose escalation study of **20** showing increase in cAMP levels in GLP-1R stably transfected HEK-293-H188 c20 cells. **26**

Figure S29. RP-HPLC trace showing the α- and β-isomer products of **5** at 7.4 and 7.9 min. **27**

Figure S30. ESI-MS of **5**, expected m/z = 1129, observed m/z = $[M+-H_2O]^{+1}$ 1111, $[M+-H_2O+H^+]^{+2}$ 556 m/z. **27**

Figure S31. Electronic absorption spectroscopy of **5** in water. **28**

Figure S32. ^{1}H NMR of **5** (400 MHz, 298K, D_2O). **29**

Figure S33. ^{1}H NMR of **5** (400 MHz, 298K, D_2O) (Aromatic). Characteristic signals (H19) of β-(6.49) and α- (6.42) aquo-isomers of **5** are observed.[1] **30**

Figure S34. RP-HPLC trace showing product **13** at 12.0 min. **31**

Figure S35. ESI-MS of **13**, expected m/z = 5341, observed m/z = $[M^+\text{-}H_2O+2H^+]^{+3}$ 1775, $[M^+\text{-}H_2O+3H^++CH_3OH]^{+4}$ 1364, $[M^+\text{-}H_2O+3H^+]^{+4}$ 1332 m/z. **31**

Figure S36. *In vitro* dose escalation study of **13** showing increase in cAMP levels in GLP-1R stably transfected HEK-293-H188 c20 cells. **32**

Figure S37. RP-HPLC trace showing product **21** at 11.8 min. **32**

Figure S38. ESI-MS of **21**, expected m/z = 5469, observed m/z = $[M^+\text{-}H_2O+2H^++CH_3CN]^{+3}$ 1859, $[M^+\text{-}H_2O+2H^+]^{+3}$ 1819, $[M^+\text{-}H_2O+3H^++CH_3OH]^{+4}$ 1395, $[M^+\text{-}H_2O+3H^+]^{+4}$ 1364, $[M^+\text{-}H_2O+4H^+]^{+5}$ 1091 m/z. **33**

Figure S39. *In vitro* dose escalation study of **21** showing increase in cAMP levels in GLP-1R stably transfected HEK-293-H188 c20 cells. **33**

Figure S40. RP-HPLC trace showing the α- and β-isomer products of **6** at 10.4 and 11.0 min. **34**

Figure S41. ESI-MS of **6**, expected m/z = 1143, observed m/z = $[M^+\text{-}H_2O]^{+1}$ 1124, $[M^+\text{-}H_2O+H^+]^{+2}$ 563 m/z. **34**

Figure S42. Electronic absorption spectra of **6** in water. **35**

Figure S43. ^{1}H NMR of **6** (400 MHz, 298K, D_2O). **36**

Figure S44. ^{1}H NMR of **6** (400 MHz, 298K, D_2O) (Aromatic). Characteristic signals (H19) of β- (6.50) and α- (6.43) aquo-isomers of **6** are observed.[1] **37**

Figure S45. RP-HPLC trace showing product **14** at 12.0 min. **38**

Figure S46. ESI-MS of **14**, expected m/z = 5355, observed m/z = $[M^+\text{-}H_2O+2H^+]^{+3}$ 1780, $[M^+\text{-}H_2O+3H^+]^{+4}$ 1335 m/z. **38**

Figure S47. *In vitro* dose escalation study of **14** showing increase in cAMP levels in GLP-1R stably transfected HEK-293-H188 c20 cells. **39**

Figure S48. RP-HPLC trace showing product **22** at 11.8 min. **39**

Figure S49. ESI-MS of **22**, expected m/z = 5483, observed m/z = $[M^+\text{-}H_2O+2H^+]^{+3}$ 1822, $[M^+\text{-}H_2O+3H^+]^{+4}$ 1367, $[M^+\text{-}H_2O+5H^+]^{+6}$ 912, $[M^+\text{-}H_2O+6H^+]^{+7}$ 781 m/z. **40**

Figure S50. *In vitro* dose escalation study of **22** showing increase in cAMP levels in GLP-1R stably transfected HEK-293-H188 c20 cells. **40**

Figure S51. RP-HPLC trace showing the α- and β-isomer products of **7** 11.3 and 11.8 min. **41**

Figure S52. ESI-MS of **7**, expected m/z = 1157, observed m/z = $[M^+\text{-}H_2O]^{+1}$ 1139, $[M^+\text{-}H_2O+H^+]^{+2}$ 570 m/z. **41**

Figure S53. Electronic absorption spectra of **7** in water. **42**

Figure S54: ^{1}H NMR of **7** (400 MHz, 298K, D_2O). **43**

Figure S55. ^{1}H NMR of **7** (400 MHz, 298K, D_2O) (Aromatic). Characteristic signals (H19) of β- (6.49) and α- (6.42) aquo-isomers of **7** are observed.[1] **44**

Figure S56. RP-HPLC trace showing product **15** at 12.0 min. **45**

Figure S57. ESI-MS of **15**, expected m/z = 5369, observed m/z = $[M^+\text{-}H_2O+2H^+]^{+3}$ 1784, $[M^+\text{-}H_2O+3H^+]^{+4}$ 1339 m/z. **45**

Figure S58. *In vitro* dose escalation study of **15** showing increase in cAMP levels in GLP-1R stably transfected HEK-293-H188 c20 cells. **46**

Figure S59. RP-HPLC trace showing product **23** at 11.8 min. **46**

Figure S60. ESI-MS of **23**, expected m/z = 5497, observed m/z = $[M^+\text{-}H_2O+2H^+]^{+3}$ 1827, $[M^+\text{-}H_2O+3H^+]^{+4}$ 1371, $[M^+\text{-}H_2O+5H^+]^{+6}$ 914, $[M^+\text{-}H_2O+6H^+]^{+7}$ 784, $[M^+\text{-}H_2O+7H^+]^{+8}$ 685 m/z. **47**

Figure S61. *In vitro* dose escalation study of **23** showing increase in cAMP levels in GLP-1R stably transfected HEK-293-H188 c20 cells. **47**

Figure S62. RP-HPLC trace showing the α- and β-isomer products of **8** at 18.4 and 19.3 min. **48**

Figure S63. ESI-MS of **8**, expected m/z = 1204, observed m/z = $[M^+\text{-}H_2O]^{+1}$ 1185, $[M^+\text{-}H_2O+H^+]^{+2}$ 593 m/z. **48**

Figure S64. Electronic absorption spectra of **8** in water. **49**

Figure S65. ^{1}H NMR of **8** (400 MHz, 298K, D_2O). **50**

Figure S66. ^{1}H NMR of **8** (400 MHz, 298K, D_2O) (Aromatic). Characteristic signals (H19) of β- (6.5003) and α- (6.4301) aquo-isomers of **8** are observed.[1] **51**

Figure S67. RP-HPLC trace showing product **16** at 11.7 min. **52**

Figure S68. ESI-MS of **16**, expected m/z = 5416, observed m/z = $[M^+\text{-}H_2O+2H^+]^{+3}$ 1800, $[M^+\text{-}H_2O+3H^+]^{+4}$ 1350 m/z. **52**

Figure S69. *In vitro* dose escalation study of **16** showing increase in cAMP levels in GLP-1R stably transfected HEK-293-H188 c20 cells. **53**

Figure S70. RP-HPLC trace showing product **24** at 11.6 min. **53**

Figure S71. ESI-MS of **24**, expected m/z = 5544, observed m/z = $[M^+\text{-}H_2O+2H^++CH_3CN]^{+3}$ 1884, $[M^+\text{-}H_2O+3H^++CH_3OH]^{+4}$ 1414, $[M^+\text{-}H_2O+3H^+]^{+4}$ 1382 m/z. **54**

Figure S72. *In vitro* dose escalation study of **24** showing increase in cAMP levels in GLP-1R stably transfected HEK-293-H188 c20 cells. **54**

Figure S73. RP-HPLC trace showing the α- and β-isomer products of **9** at 20.0 and 21.8 min. **55**

Figure S74. ESI-MS of **9**, expected m/z = 1292, observed m/z = $[M^+-H_2O]^{+1}$ 1273, $[M^+-H_2O+H^+]^{+2}$ 637 m/z. **55**

Figure S75. Electronic absorption spectra of **9** in water. **56**

Figure S76. ^{1}H NMR of **9** (400 MHz, 298K, D_2O). **57**

Figure S77. ^{1}H NMR of **9** (400 MHz, 298K, D_2O) (Aromatic). Characteristic signals (H19) of β- (6.50) and α- (6.43) aquo-isomers of **9** are observed.[1] **58**

Figure S78. RP-HPLC trace showing product **17** at 12.3 min. **59**

Figure S79. ESI-MS of **17**, expected m/z = 5504, observed m/z = $[M^+-H_2O+2H^++CH_3CN]^{+3}$ 1870, $[M^+-H_2O+3H^++CH_3OH]^{+4}$ 1404, $[M^+-H_2O+4H^+]^{+5}$ 1098 m/z. **59**

Figure S80. *In vitro* dose escalation study of **17** showing increase in cAMP levels in GLP-1R stably transfected HEK-293-H188 c20 cells. **60**

Figure S81. RP-HPLC trace showing product **25** at 12.0 min. **60**

Figure S82. ESI-MS of **25**, expected m/z = 5632, observed m/z = $[M^+-H_2O+2H^++CH_3CN]^{+3}$ 1914, $[M^+-H_2O+3H^++CH_3OH]^{+4}$ 1436 m/z. **61**

Figure S83. *In vitro* dose escalation study of **25** showing increase in cAMP levels in GLP-1R stably transfected HEK-293-H188 c20 cells. **61**

Figure S84. RP-HPLC trace showing the α- and β- isomer products of **10** at 12.4 and 12.8 min. **62**

Figure S85. ESI-MS of **10**, expected m/z = 1191, observed m/z = $[M^+-H_2O]^{+1}$ 1173. **62**

Figure S86. Electronic absorption spectra of **10** in water. **63**

Figure S87. ^{1}H NMR of **10** (400 MHz, 298K, D_2O). **64**

Figure S88. ^{1}H NMR of **10** (400 MHz, 298K, D_2O) (Aromatic). Characteristic Signals (H19) of β- (6.49) and α- (6.43) aquo-isomers of **10** are Identified.[1] Additional peak groupings between 7.56-7.51 and 7.35-7.28 are indicative of the phenyl ring linker. **65**

Figure S89. RP-HPLC trace showing product **18** at 12.2 min. **66**

Figure S90. ESI-MS of **18**, expected m/z = 5403, observed m/z = $[M^+-H_2O+2H^+]^{+3}$ 1796, $[M^+-H_2O+CH_3OH+3H^+]^{+4}$ 1379, $[M^+-H_2O+3H^+]^{+4}$ 1347 m/z. **66**

Figure S91. *In vitro* dose escalation study of **18** showing increase in cAMP levels in GLP-1R stably transfected HEK-293-H188 c20 cells. **67**

Figure S92. RP-HPLC trace showing product **26** at 11.9 min. **67**

Figure S93. ESI-MS of **26**, expected m/z = 5531, observed m/z = $[M^+-H_2O+CH_3OH+3H^+]^{+4}$ 1411, $[M^+-H_2O+3H^+]^{+4}$ 1379, , $[M-H_2O+4H^+-CN]^{+4}$ 1353, **68**

$[M^+-H_2O+4H^+]^{+5}$ 1104 m/z.
Figure S94. *In vitro* dose escalation study of **26** showing increase in cAMP levels in GLP-1R stably transfected HEK-293-H188 c20 cells. **68**
Figure S95. RP-HPLC trace showing the α- and β- isomer products of **11** at 12.6 and 13.0 min. **69**
Figure S96. ESI-MS of **11**, expected m/z = 1191, observed m/z = $[M^+-H_2O]^{+1}$ 1172, $[M^+-H_2O+H^+]^{+2}$ 587 m/z. **69**
Figure S97. Electronic absorption spectra of **11** in water. **70**
Figure S98. ^{1}H NMR of **11**. (400 MHz, 298K, D_2O). **71**
Figure S99. ^{1}H NMR of **11**. (400 MHz, 298K, D_2O) (Aromatic). Characteristic signals (H19) of β- (6.49) and α- (6.42) aquo-isomers of **11** are observed.[1] Additional peak groupings between 7.48-7.37 are indicative of the phenyl ring linker. **72**
Figure S100. RP-HPLC trace showing product **19** at 12.6 min. **73**
Figure S101. ESI-MS of **19**, expected m/z = 5403, observed m/z = $[M^+-H_2O+2H^+]^{+3}$ 1796, $[M^+-H_2O+CH_3OH+3H^+]^{+4}$ 1379, $[M^+-H_2O+3H^+]^{+4}$ 1347 m/z. **73**
Figure S102. *In vitro* dose escalation study of **19** showing increase in cAMP levels in GLP-1R stably transfected HEK-293-H188 c20 cells. **74**
Figure S103. RP-HPLC trace showing product **27** at 11.9 min. **74**
Figure S104. ESI-MS of **27**, expected m/z = 5531, observed m/z = $[M^+-H_2O+CH_3CN+2H^+]^{+3}$ 1880, $[M^+-H_2O+2H^+]^{+3}$ 1838, $[M^+-H_2O+CH_3OH+3H^+]^{+4}$ 1410, $[M^+-H_2O+3H^+]^{+4}$ 1379, $[M^+-H_2O+4H^+]^{+5}$ 1103, $[M^+-H_2O+5H^+]^{+6}$ 919 m/z. **75**
Figure S105. *In vitro* dose escalation study of **27** showing increase in cAMP levels in GLP-1R stably transfected HEK-293-H188 c20 cells. **75**
Figure 106. *In vitro* dose escalation study of Ex40 showing increase in cAMP levels in rGLP-1R transiently transfected HEK-293-H188 c24 cells. **76**
Figure 107. Non-linear regression dose response analysis with hill slope of Ex40. **77**
Figure 108. *In vitro* dose escalation study of **22** showing increase in cAMP levels in rGLP-1R transiently transfected HEK-293-H188 c24 cells. **78**
Figure 109. Non-linear regression dose response analysis with hill slope of **22.** **79**
Figure 110. *In vitro* dose escalation competition binding studies of **12**-**27** compared with Ex4 and Ex40 controls against fluorescent GLP-1red. **79**

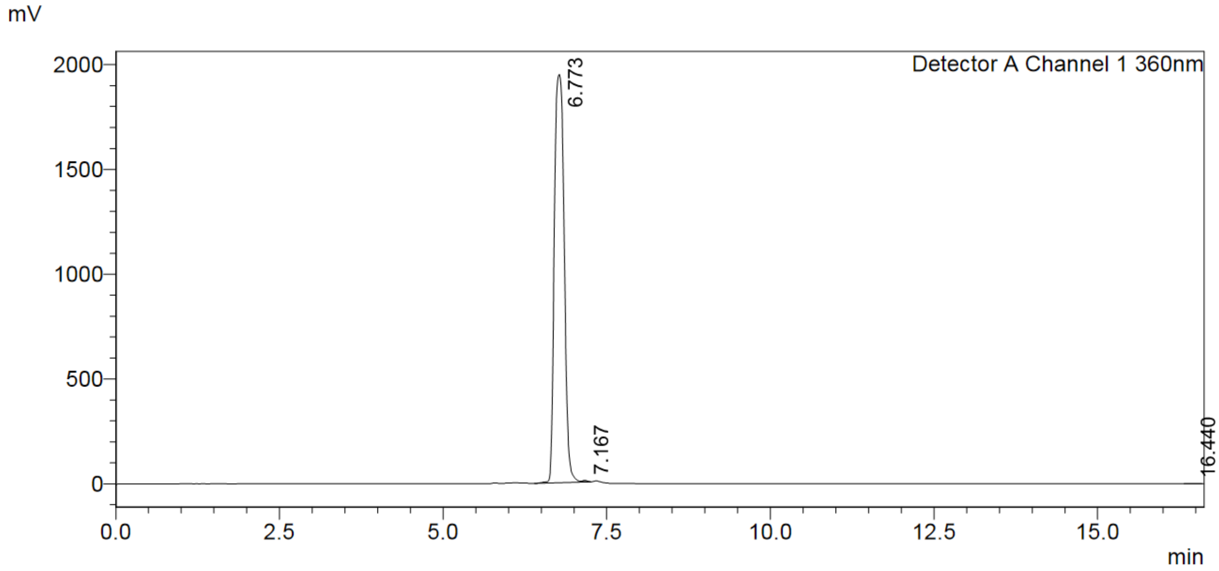

Figure S1. RP-HPLC trace of commercial vitamin B12 at 6.8 min.

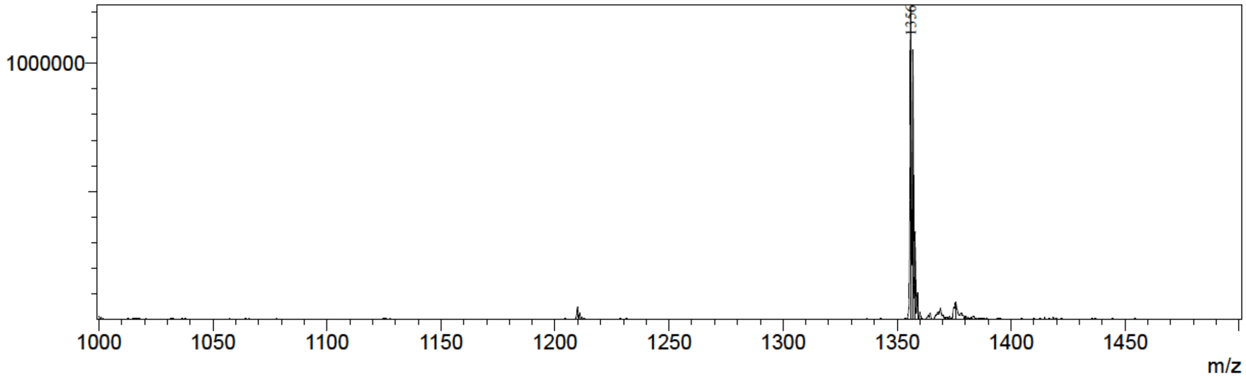

Figure S2. ESI-MS of commercial vitamin B12, expected m/z = 1355, observed m/z = $[M^{+}+H^{+}]^{+1}$ 1356.

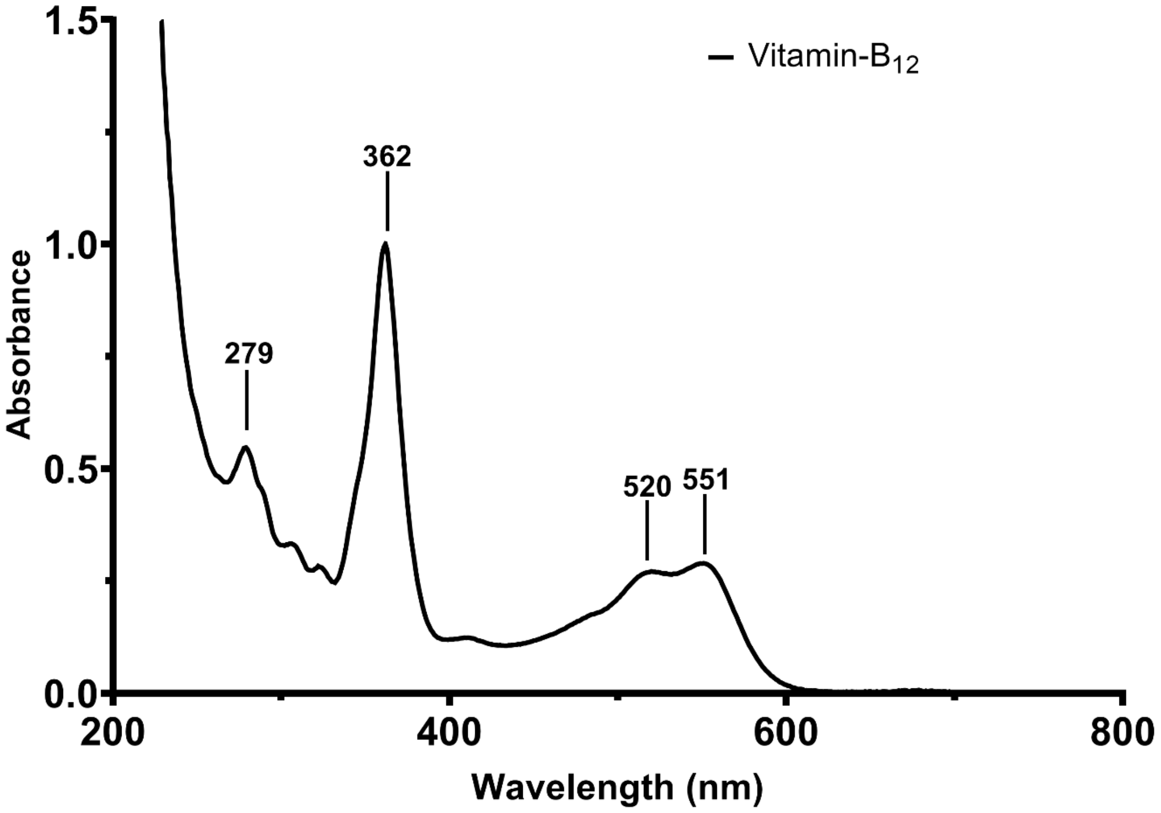

Figure S3. Electronic absorption spectroscopy of commercial vitamin B12 in water used herein.

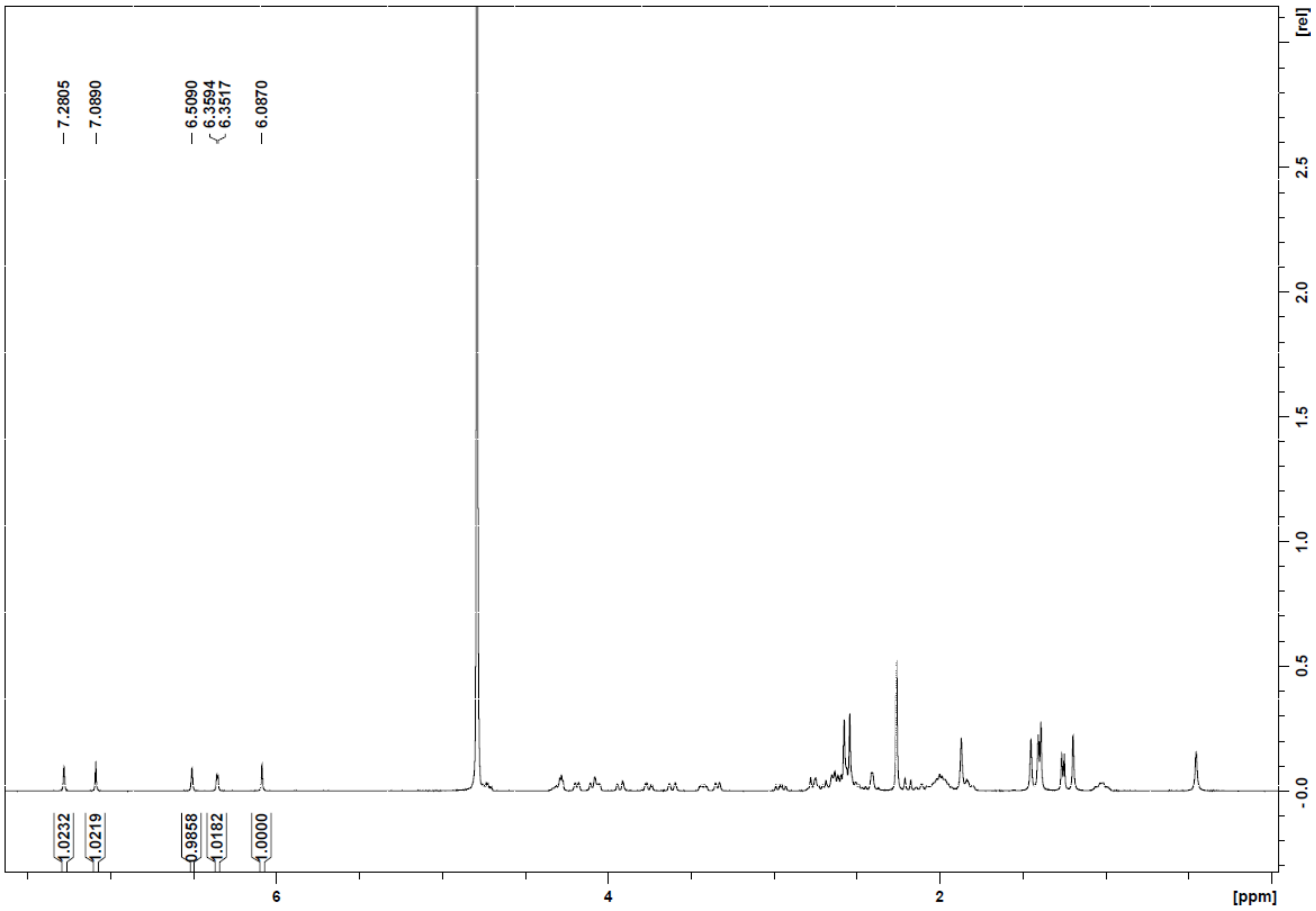

Figure S4. ^{1}H NMR of commercial vitamin B12 (400 MHz, 298K, D_2O) used herein.

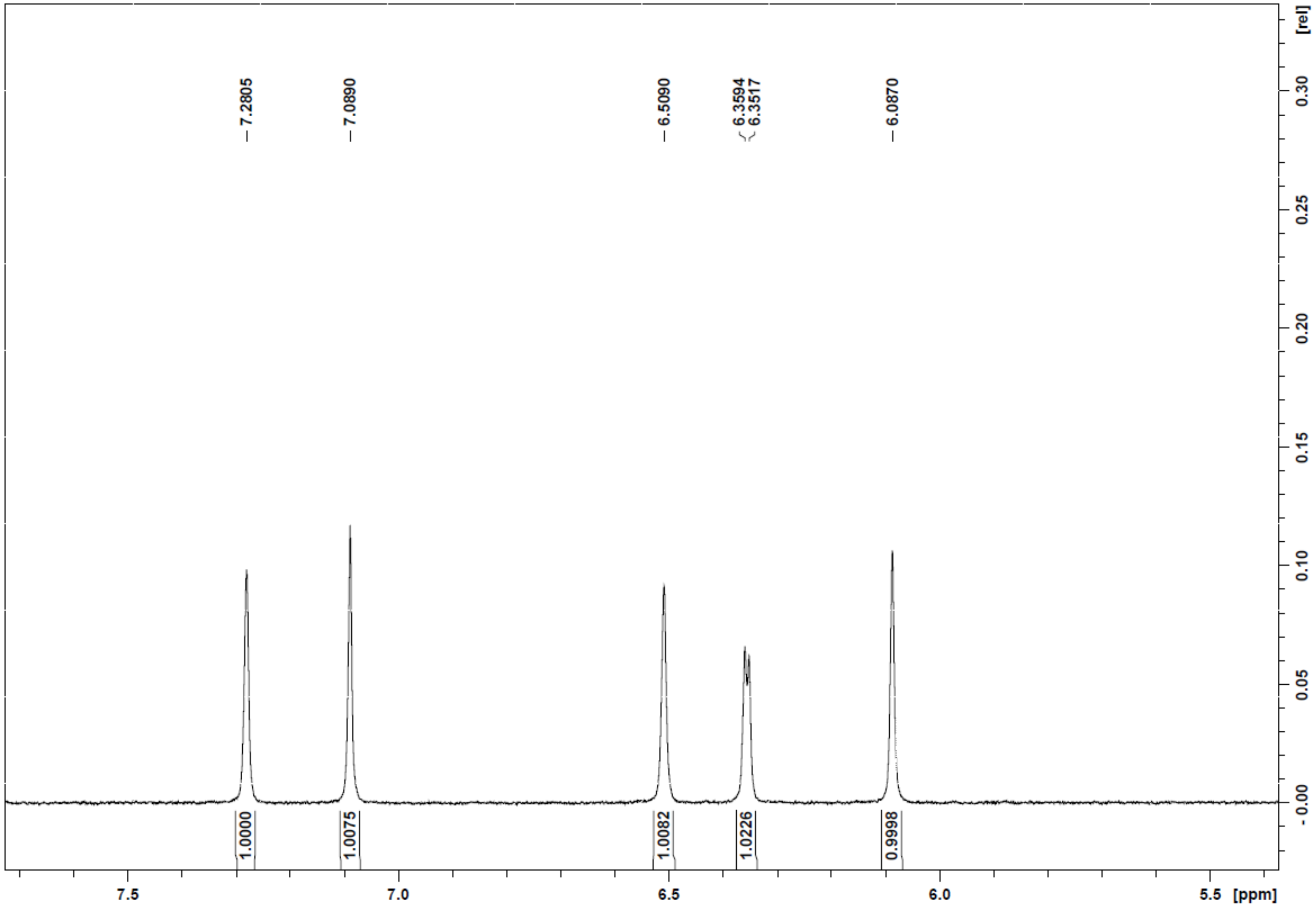

Figure S5. ^{1}H NMR of commercial vitamin B12 (400 MHz, 298K, D_2O) (Aromatic region enlarged).

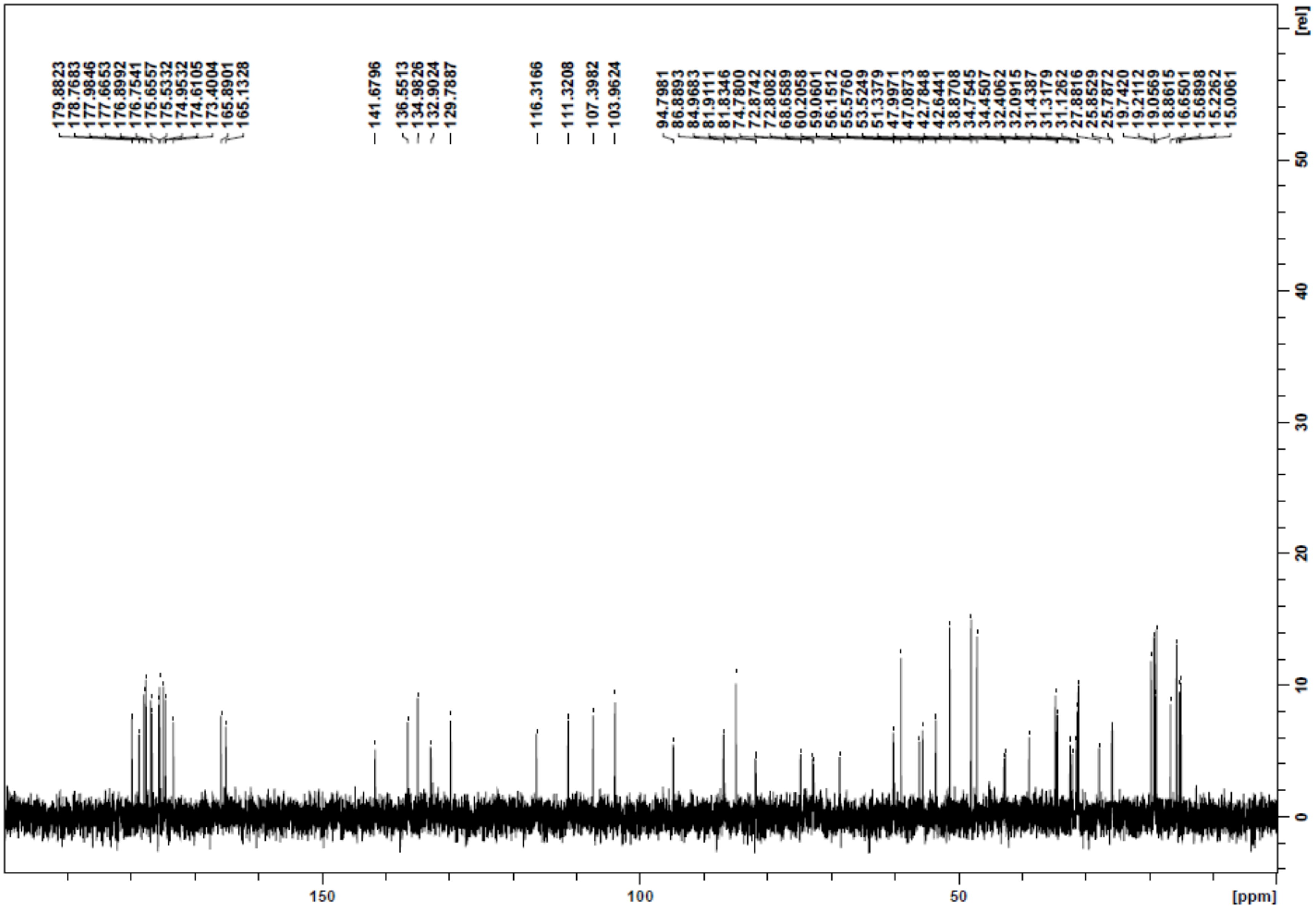

Figure S6. ^{13}C NMR of commercial vitamin B12 (400 MHz, 298K, D_2O).

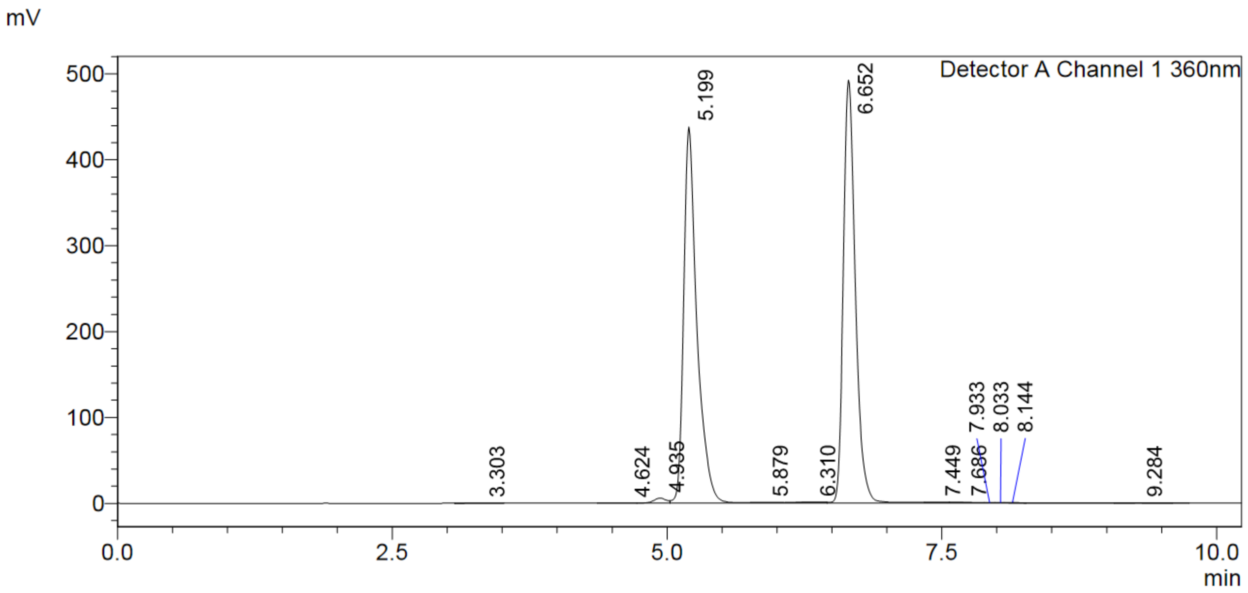

Figure S7. RP-HPLC trace showing the α- and β-isomer products of **2** at 5.2 and 6.7 min.

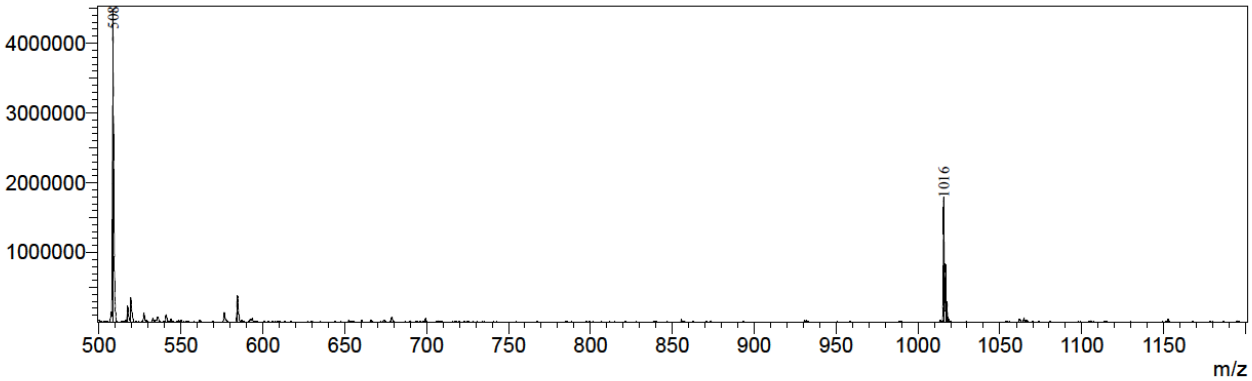

Figure S8. ESI-MS of **2**, expected m/z = 1042, observed m/z = $[M\text{-}CN]^{+1}$ 1016.

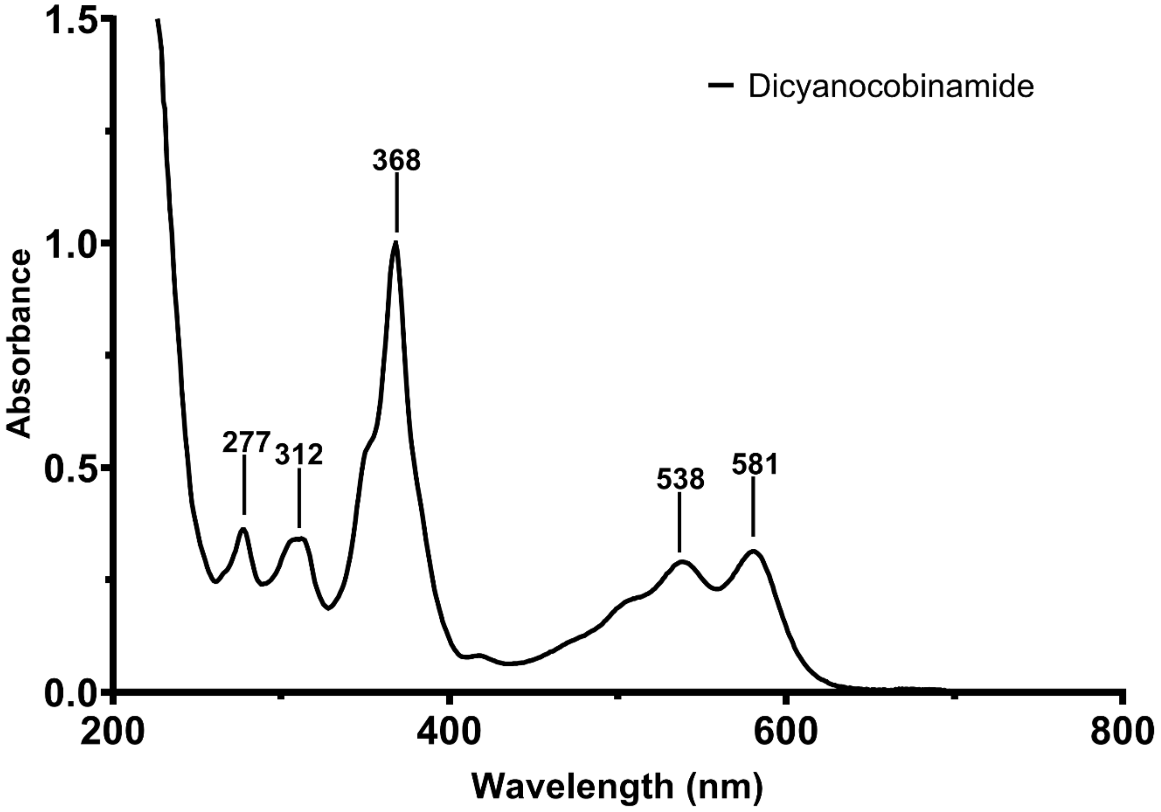

Figure S9. Electronic absorption spectroscopy of **2** in water.

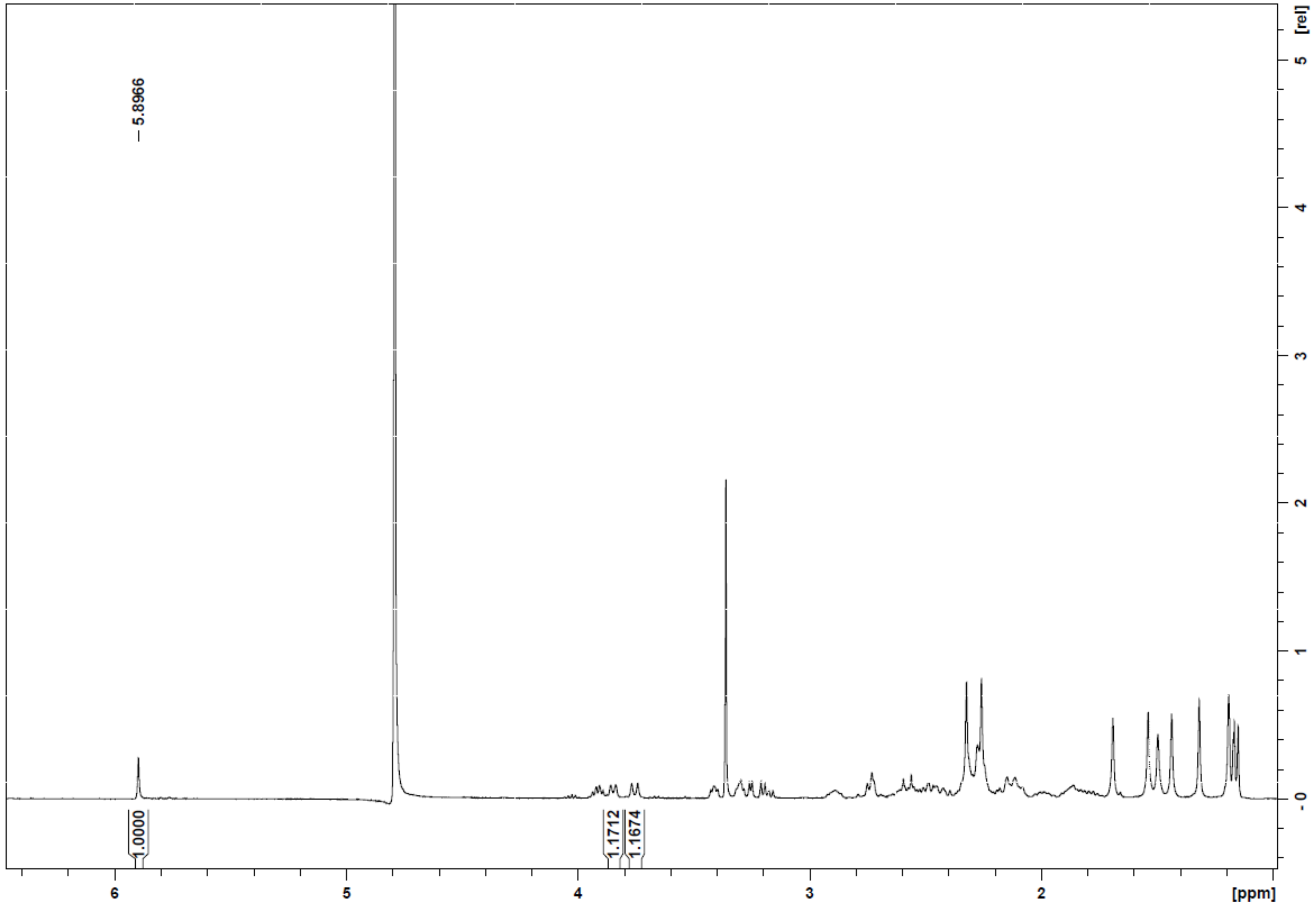

Figure S10. ^{1}H NMR of **2** (400 MHz, 298K, D_2O).

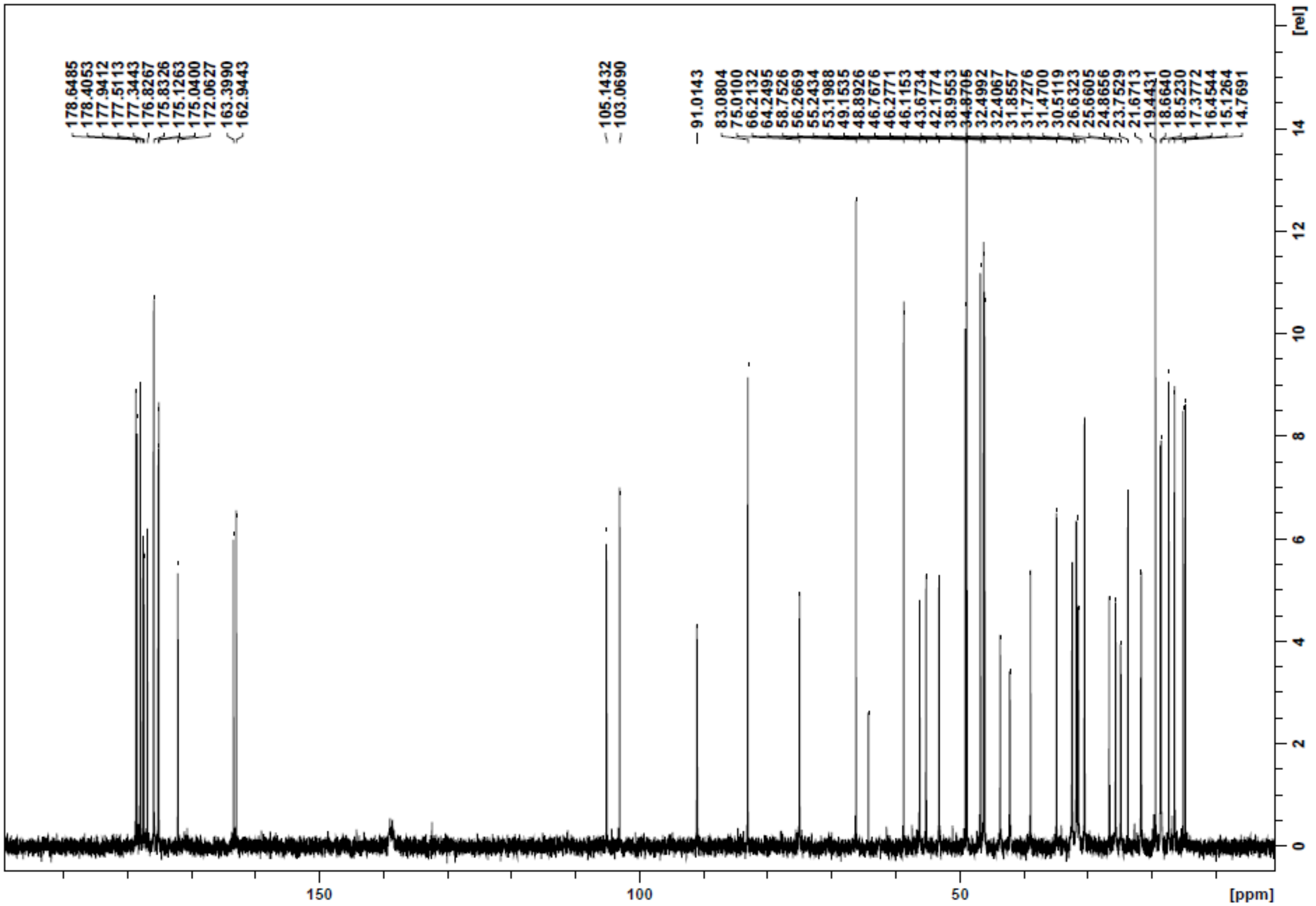

Figure S11. ^{13}C NMR of **2** (400 MHz, 298K, D_2O).

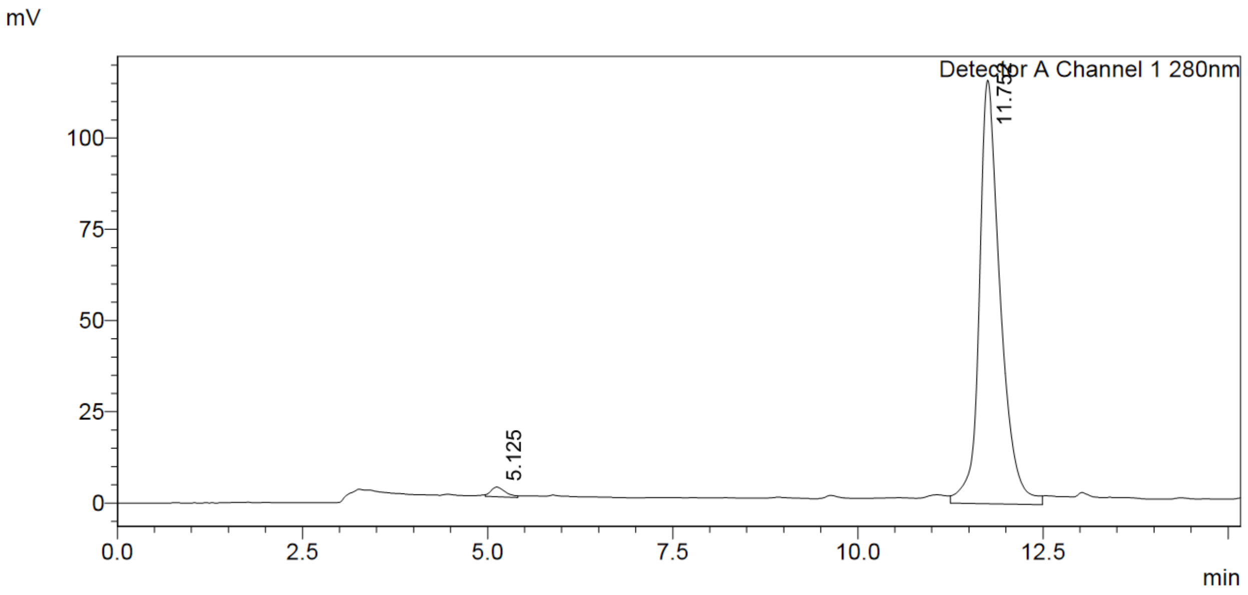

Figure S12. RP-HPLC trace showing Ex4 at 11.8 min.

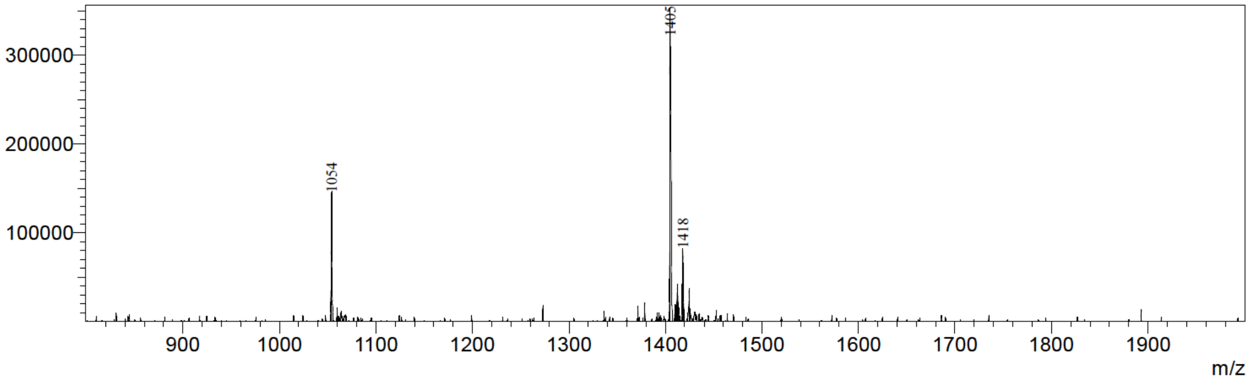

Figure S13. ESI-MS of Ex4, expected m/z = 4213, observed m/z = $[M+3H^{+}]^{+3}$ 1405, $[M+4H^{+}]^{+4}$ 1054 m/z.

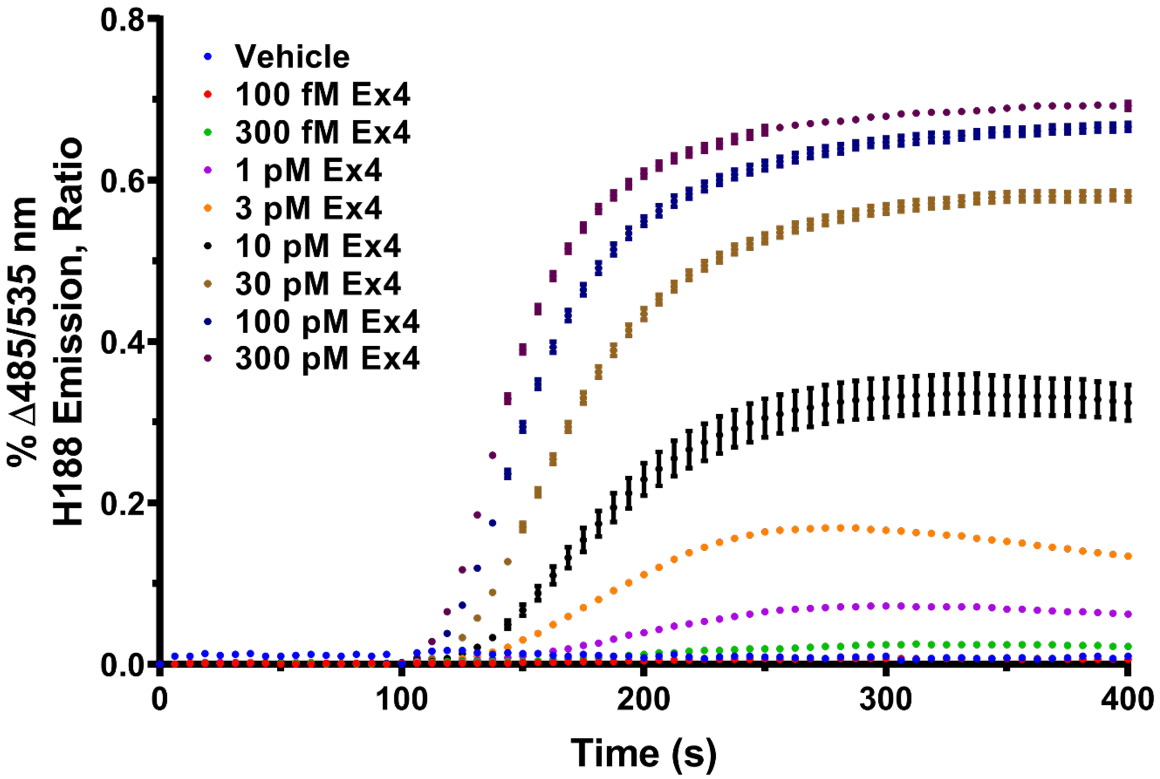

Figure S14. *In vitro* dose escalation study of Ex4 showing increase in cAMP levels in GLP-1R stably transfected HEK-293-H188 c20 cells.

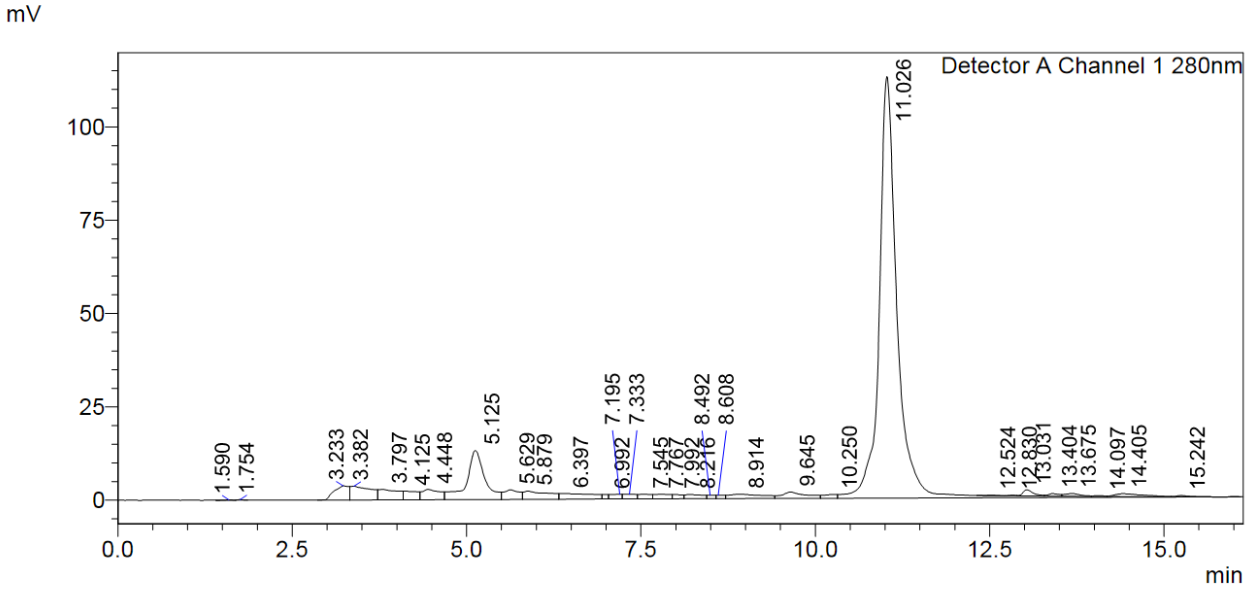

Figure S15. RP-HPLC trace showing Ex40 at 11.0 min.

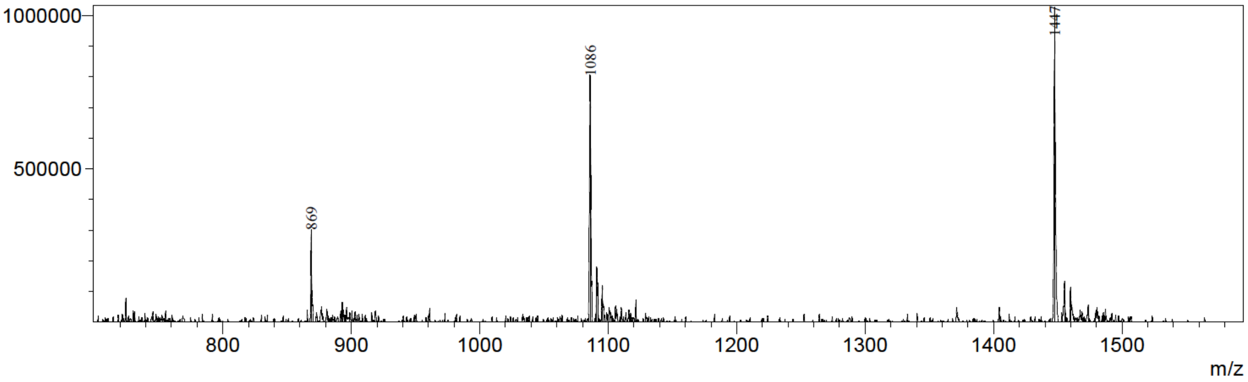

Figure S16. ESI-MS of Ex40, expected m/z = 4341, observed m/z = $[M+3H^+]^{+3}$ 1447, $[M+4H^+]^{+4}$ 1086, $[M+5H^+]^{+5}$ 869 m/z.

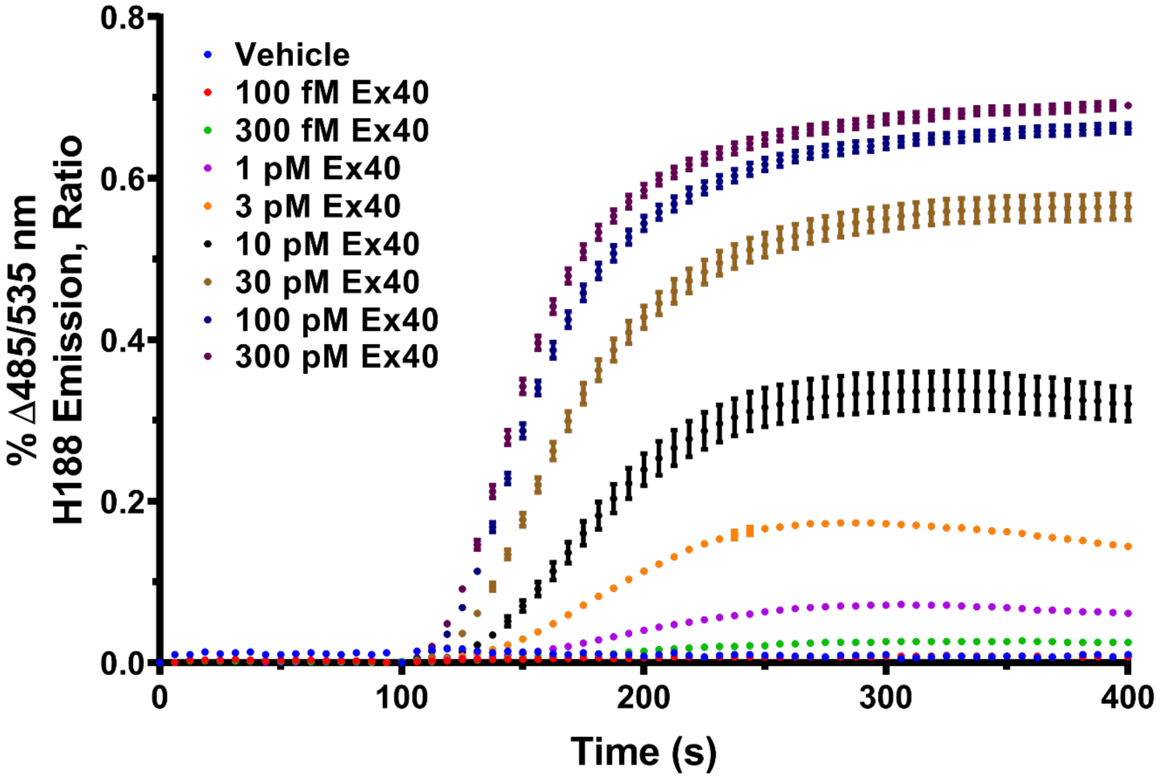

Figure S17. *In vitro* dose escalation study of Ex40 showing increase in cAMP levels in GLP-1R stably transfected HEK-293-H188 c20 cells.

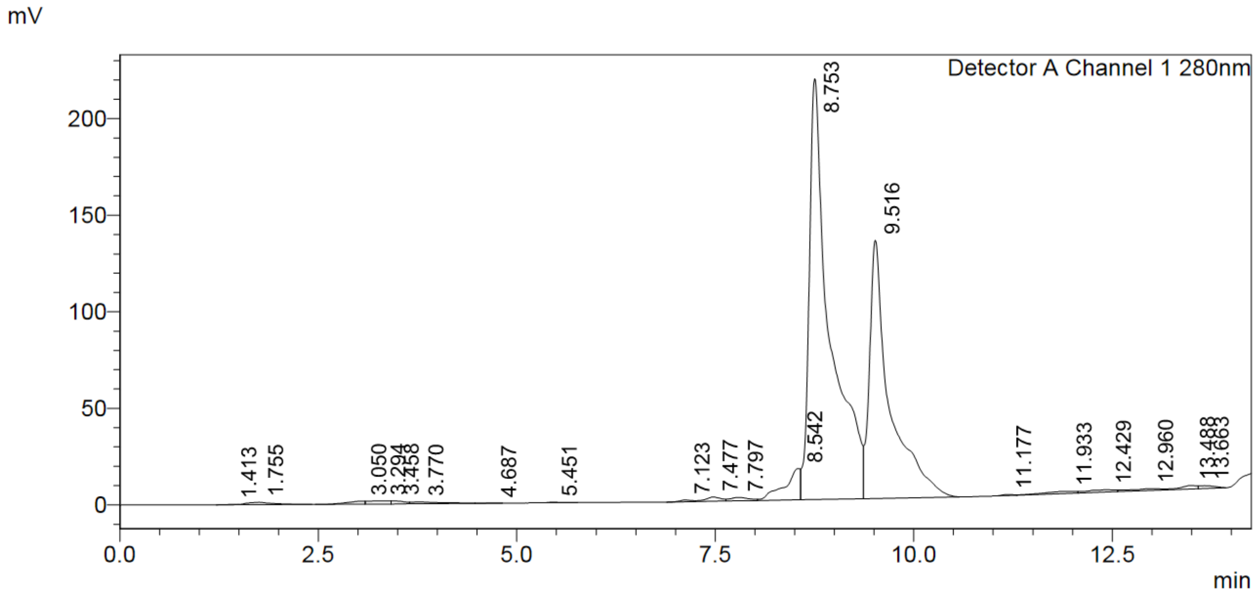

Figure S18. RP-HPLC trace showing the α- and β-isomer products of **4** at 8.8 and 9.5 min.

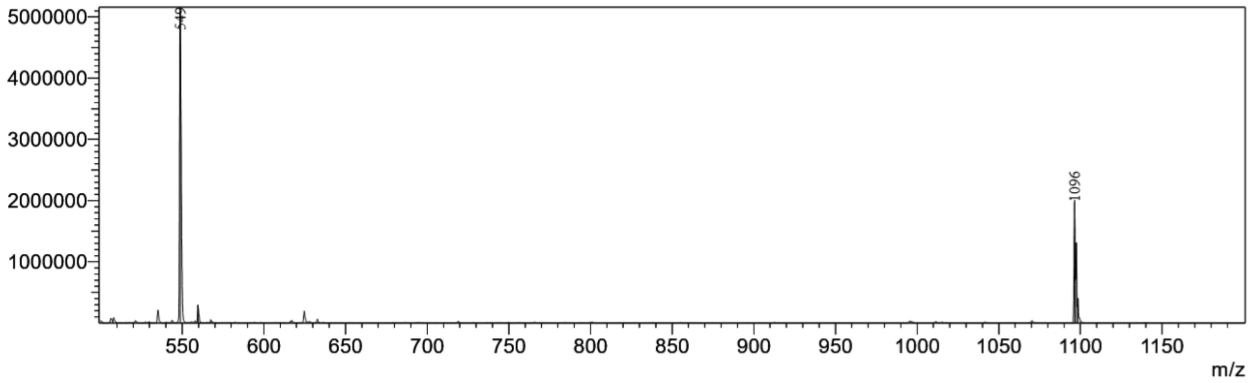

Figure S19. ESI-MS of **4**, expected m/z = 1115, observed m/z = $[M^{+}-H_2O]^{+1}$ 1096, $[M^{+}-H_2O+H^{+}]^{+2}$ 549 m/z.

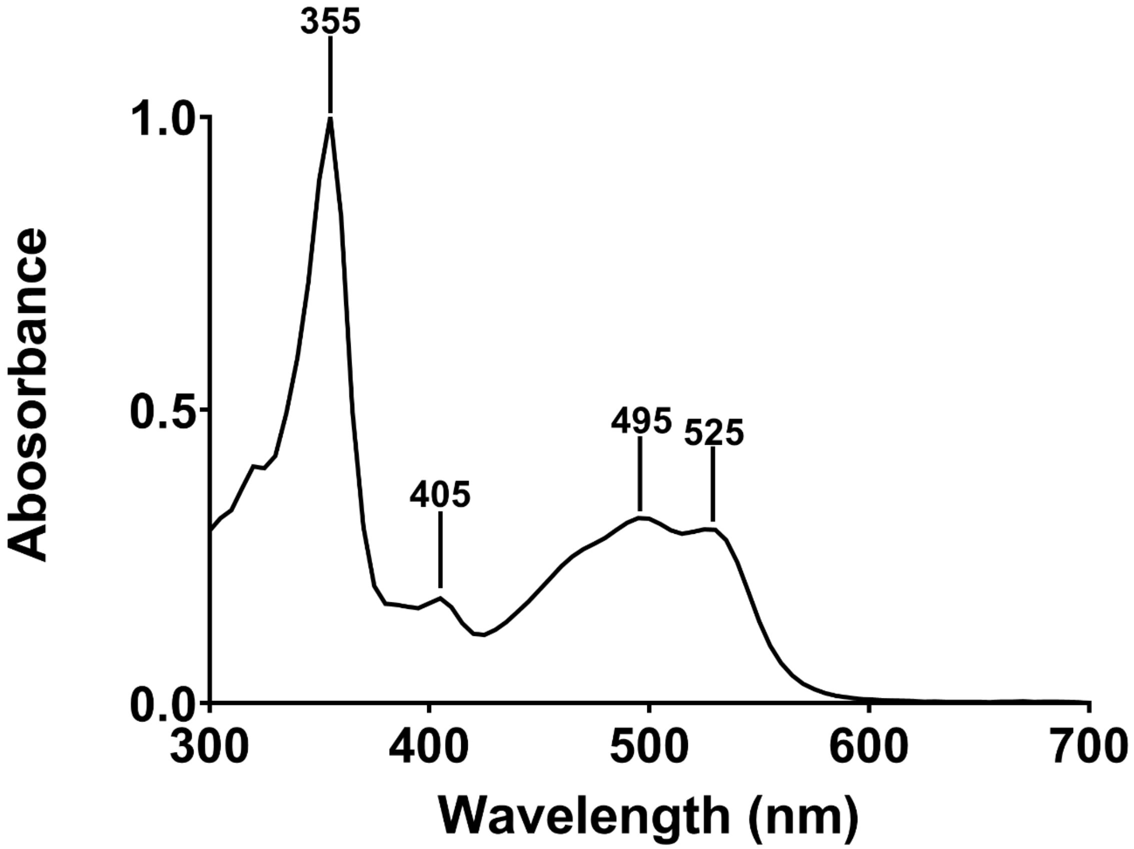

Figure S20. Electronic absorption spectroscopy of **4** in water.

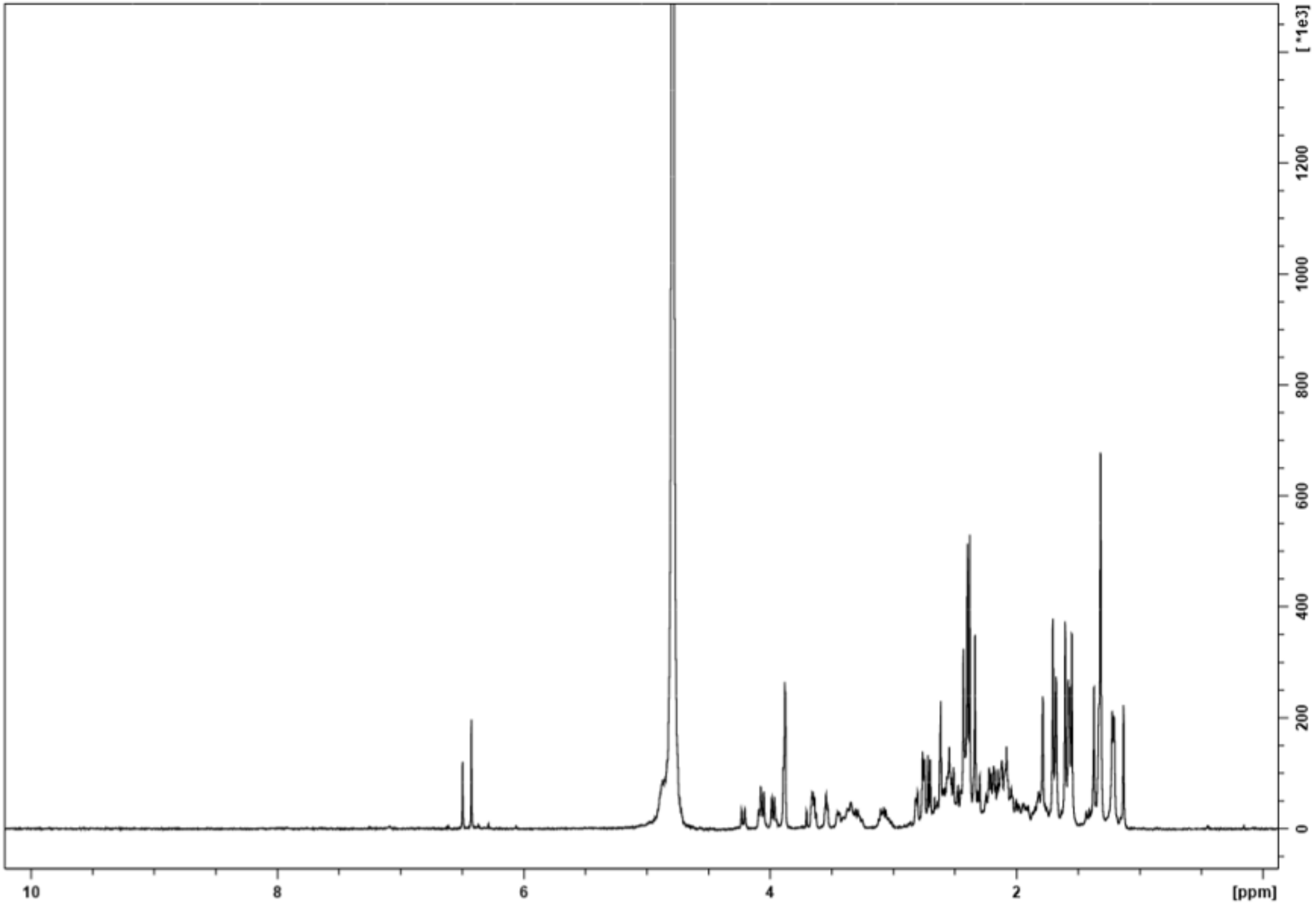

Figure S21. ^{1}H NMR of **4**. (400 MHz, 298K, D_2O).

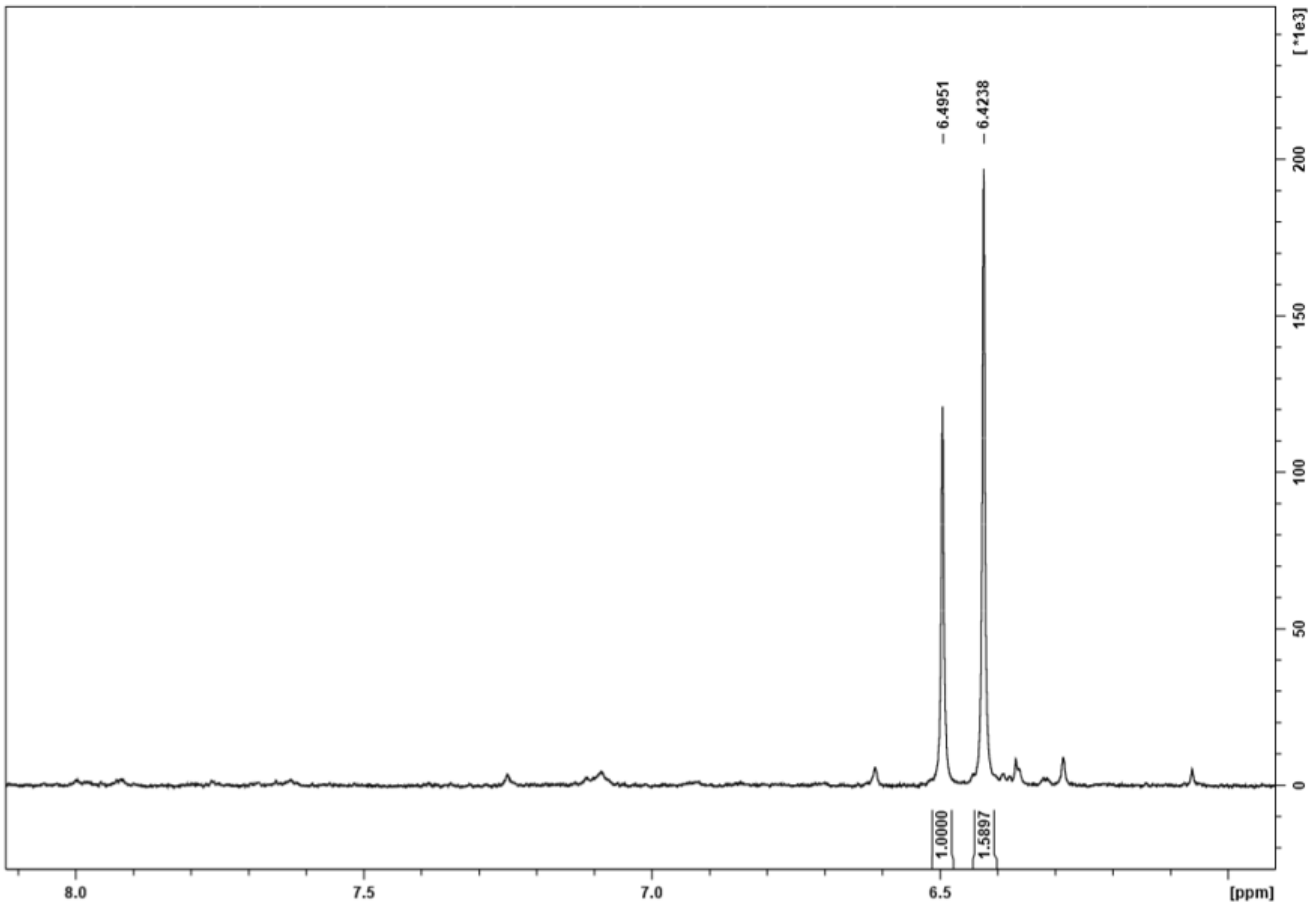

Figure S22. ^{1}H NMR of **4**. (400 MHz, 298K, D_2O) (Aromatic). Characteristic signals (H19) of β- (6.50) and α- (6.42) aquo-isomers of **4** are observed.

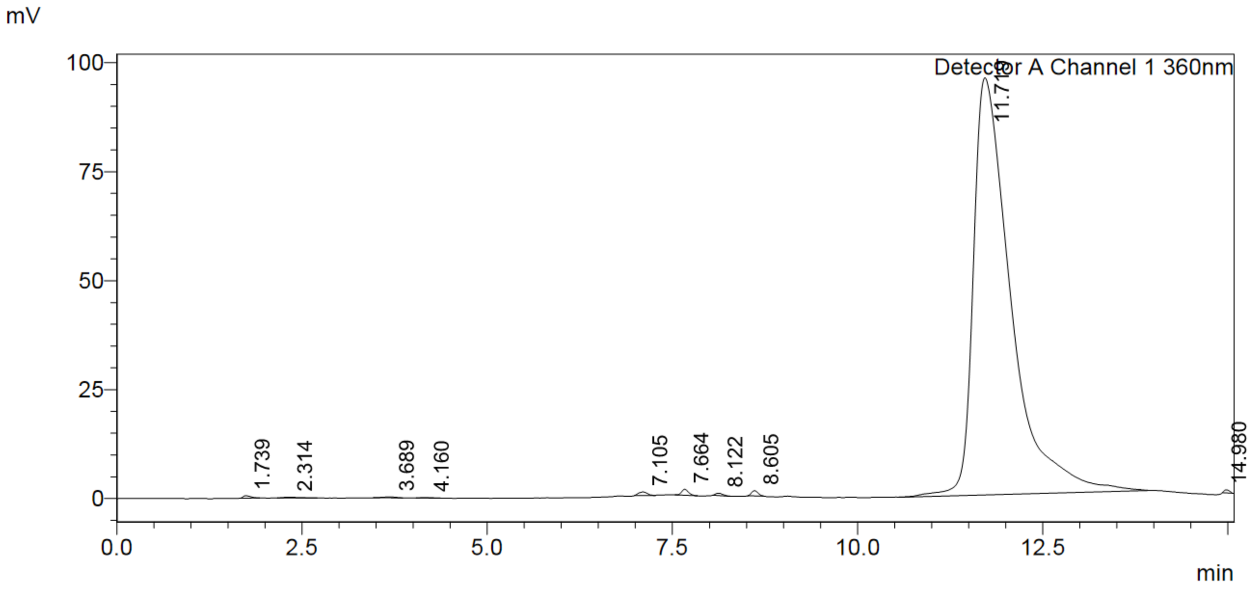

Figure S23. RP-HPLC trace showing product **12** at 11.7 min.

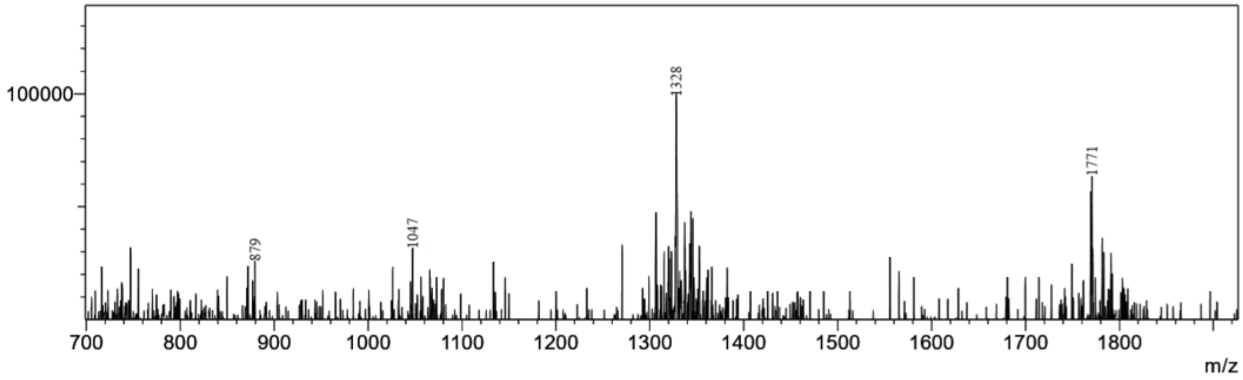

Figure S24. ESI-MS of **12**, expected m/z = 5327, observed m/z = $[M^{+}\text{-}H_2O+2H^{+}]^{+3}$ 1771, $[M^{+}\text{-}H_2O+3H^{+}]^{+4}$: 1328 m/z.

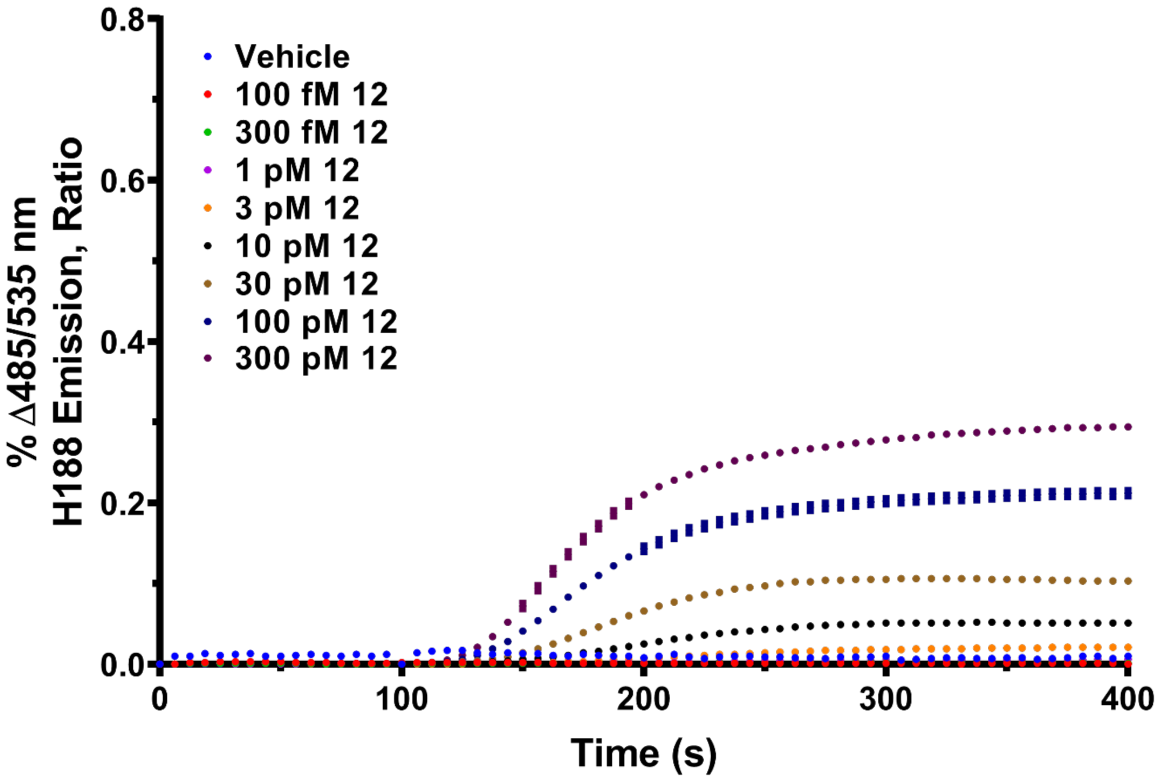

Figure S25. *In vitro* dose escalation study of **12** showing increase in cAMP levels in GLP-1R stably transfected HEK-293-H188 c20 cells.

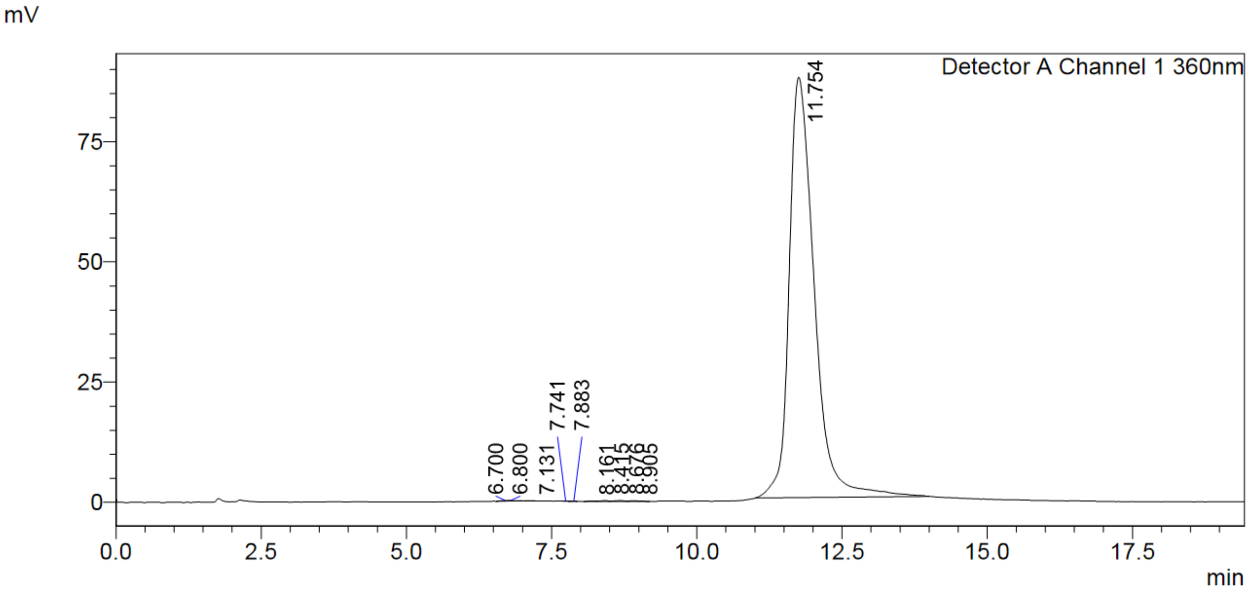

Figure S26. RP-HPLC trace showing product **20** at 11.8 min.

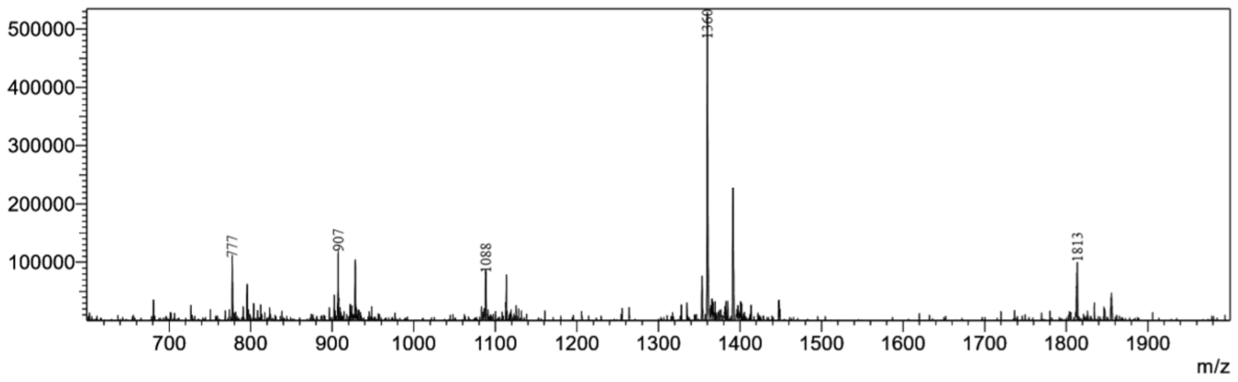

Figure S27. ESI-MS of **20**, expected m/z = 5456, observed m/z = $[M^{+}\text{-}H_2O+2H^{+}]^{+3}$ 1813, $[M^{+}\text{-}H_2O+3H^{+}]^{+4}$ 1360, $[M^{+}\text{-}H_2O+4H^{+}]^{+5}$ 1088, $[M^{+}\text{-}H_2O+5H^{+}]^{+6}$ 907, $[M^{+}\text{-}H_2O+6H^{+}]^{+7}$ 777 m/z.

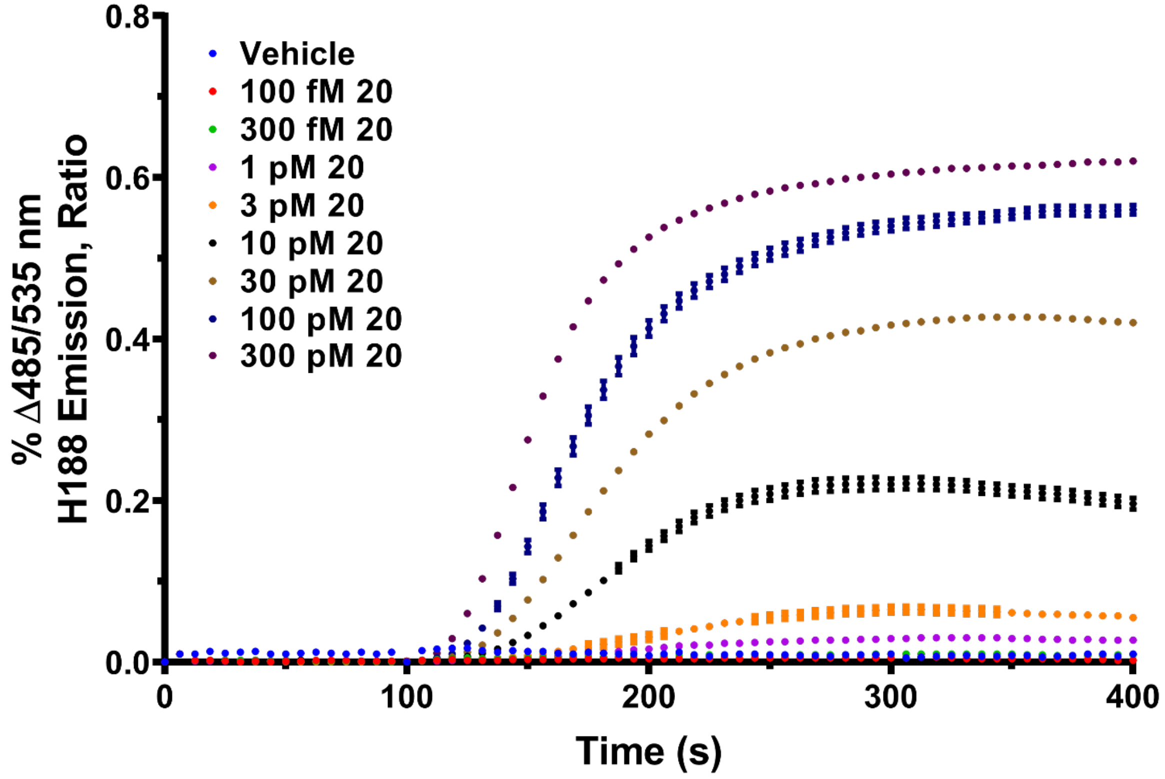

Figure S28. *In vitro* dose escalation study of **20** showing increase in cAMP levels in GLP-1R stably transfected HEK-293-H188 c20 cells.

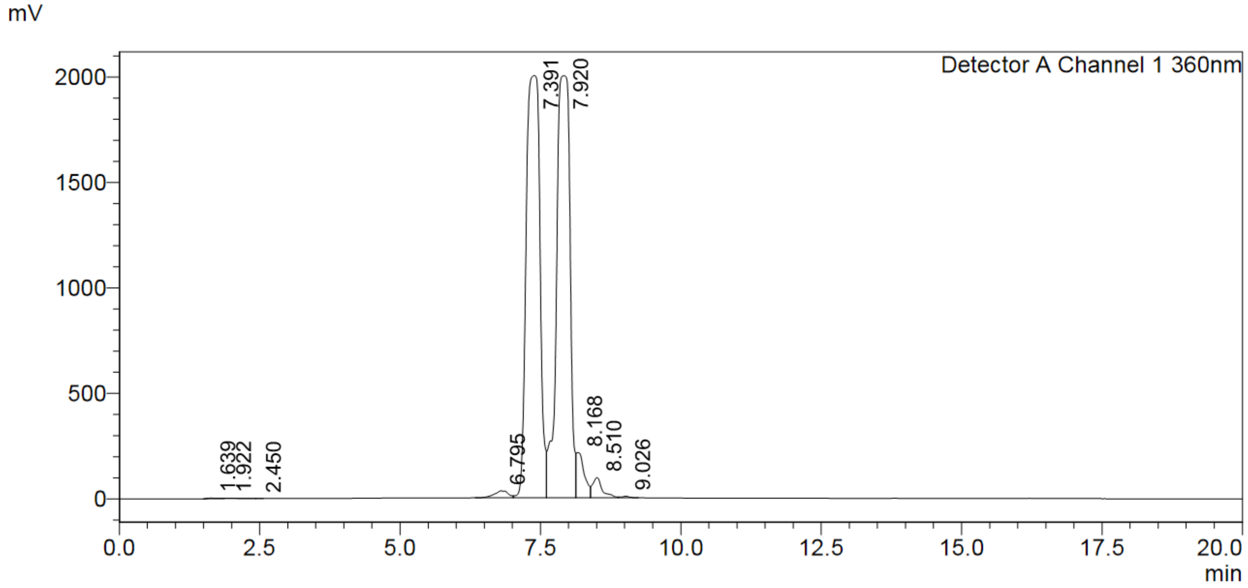

Figure S29. RP-HPLC trace showing the α- and β-isomer products of **5** at 7.4 and 7.9 min.

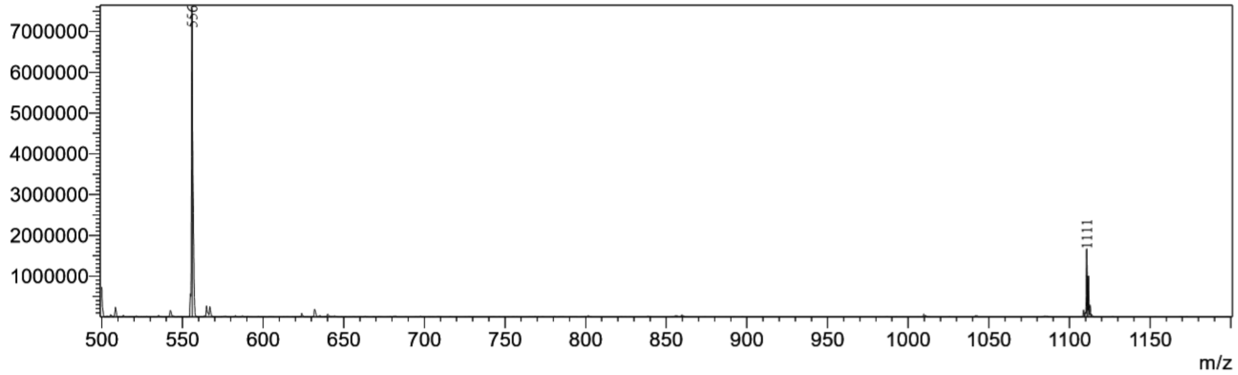

Figure S30. ESI-MS of **5**, expected m/z = 1129, observed m/z = $[M+-H_2O]^{+1}$ 1111, $[M+-H_2O+H^+]^{+2}$ 556 m/z.

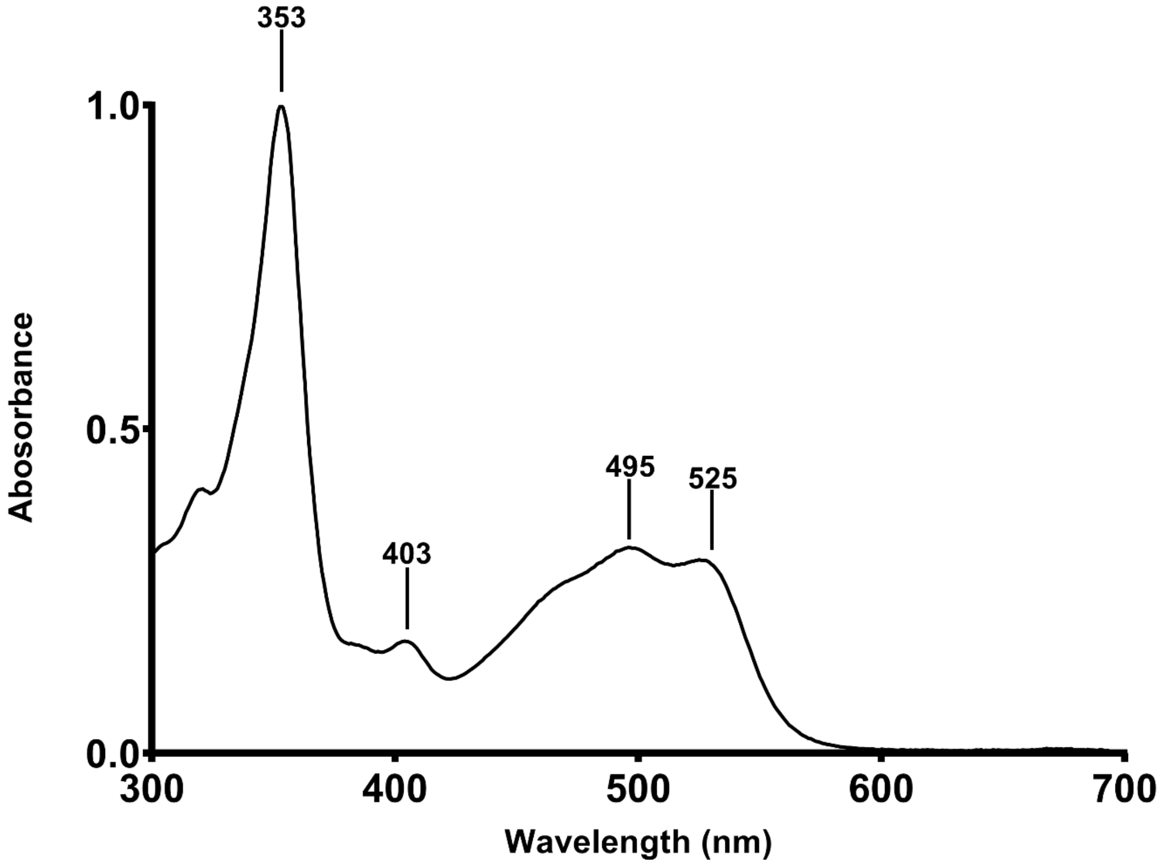

Figure S31. Electronic absorption spectroscopy of **5** in water.

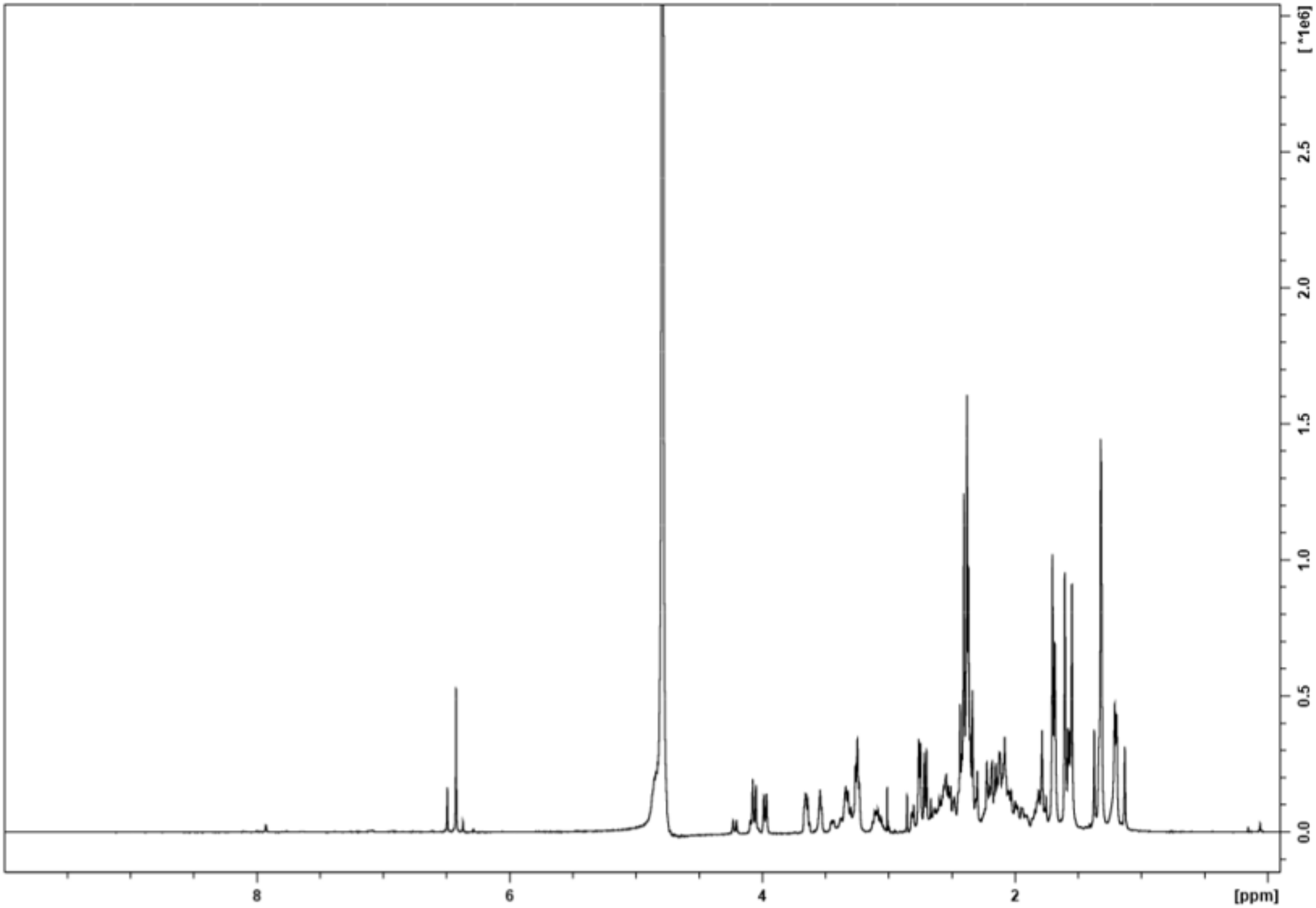

Figure S32. ^{1}H NMR of **5** (400 MHz, 298K, D_2O).

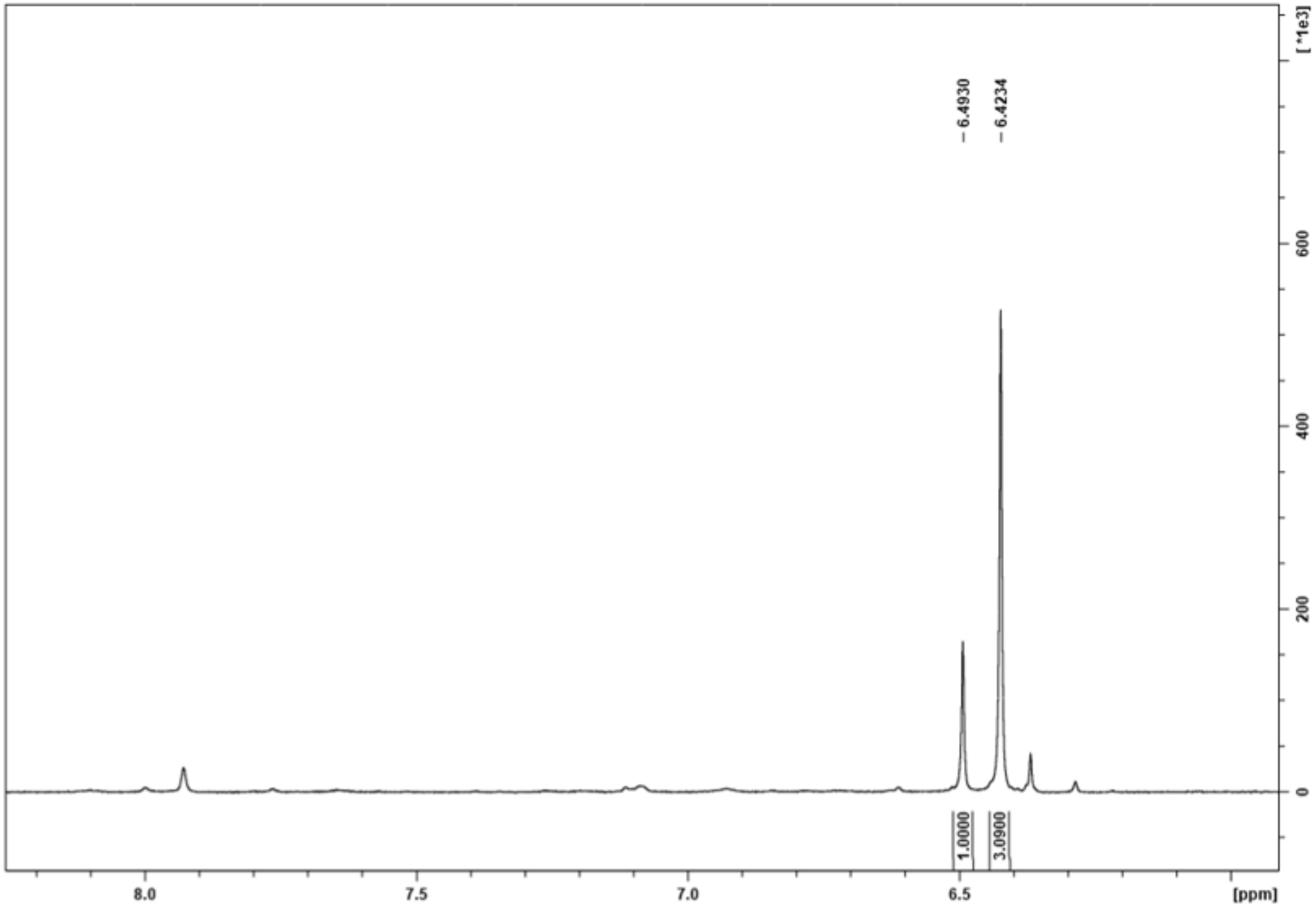

Figure S33. ^{1}H NMR of **5** (400 MHz, 298K, D_2O) (Aromatic). Characteristic signals (H19) of β-(6.49) and α- (6.42) aquo-isomers of **5** are observed.

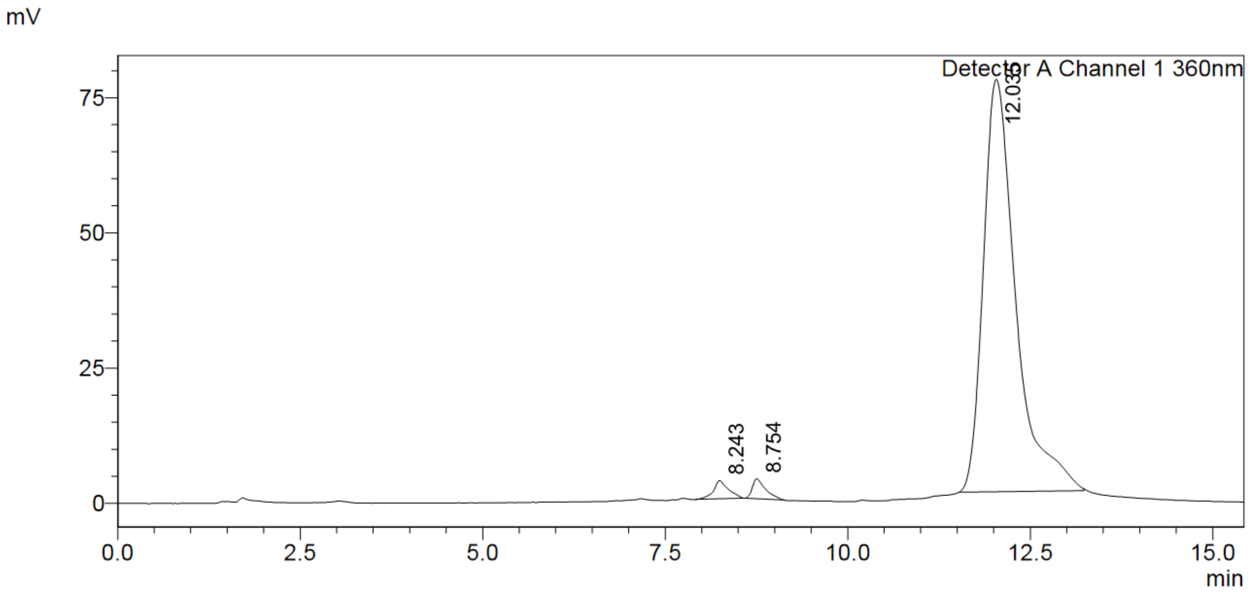

Figure S34. RP-HPLC trace showing product **13** at 12.0 min.

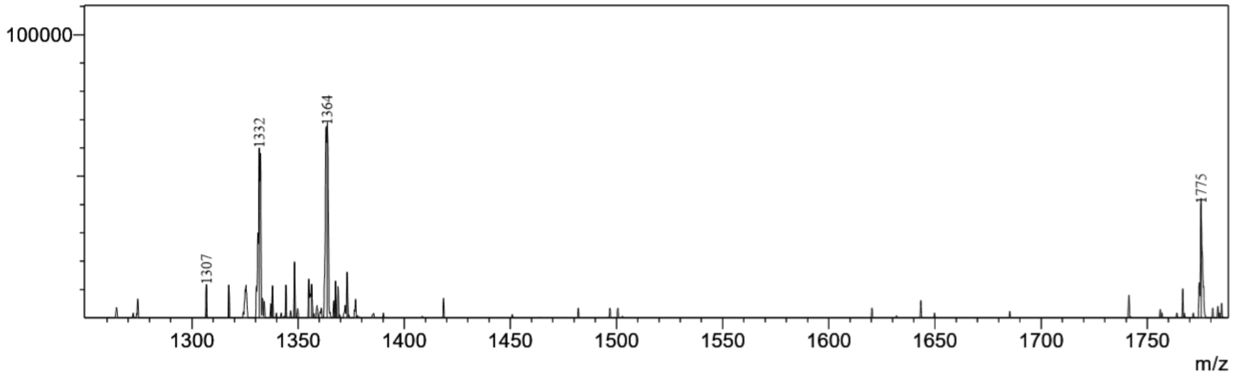

Figure S35. ESI-MS of **13**, expected m/z = 5341, observed m/z = $[M^{+}\text{-}H_2O+2H^{+}]^{+3}$ 1775, $[M^{+}\text{-}H_2O+3H^{+}+CH_3OH]^{+4}$ 1364, $[M^{+}\text{-}H_2O+3H^{+}]^{+4}$ 1332 m/z.

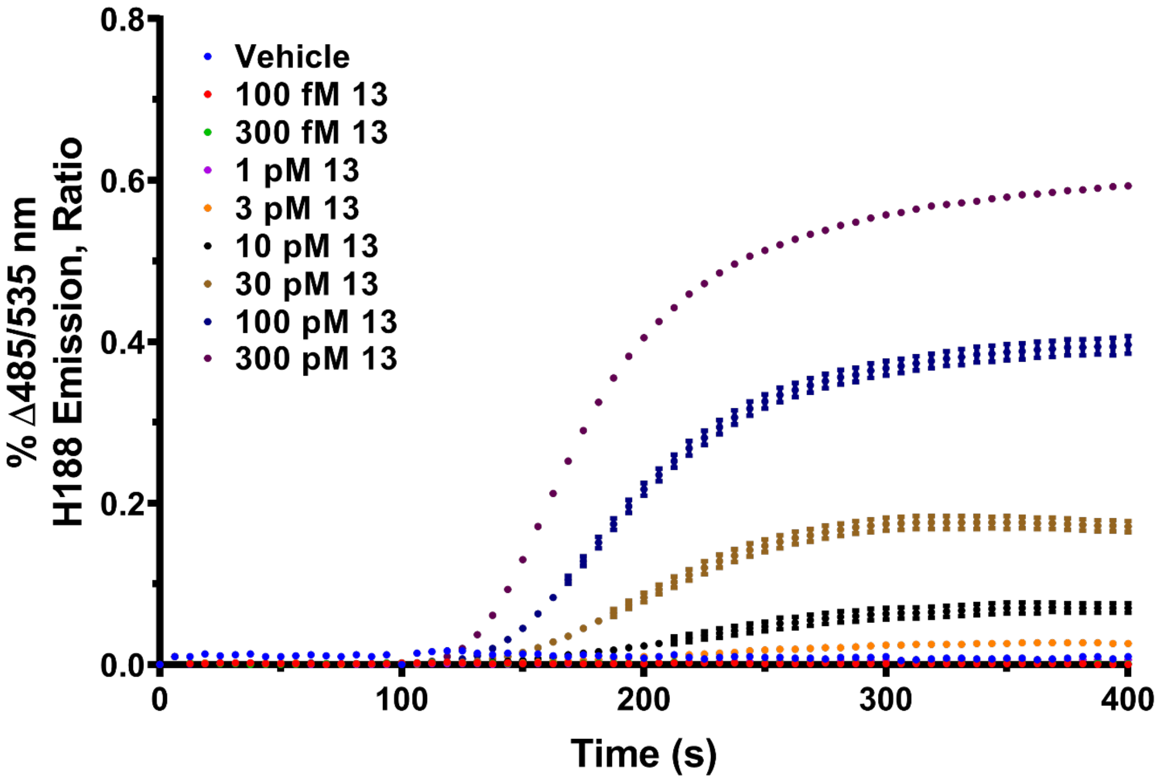

Figure S36. *In vitro* dose escalation study of **13** showing increase in cAMP levels in GLP-1R stably transfected HEK-293-H188 c20 cells.

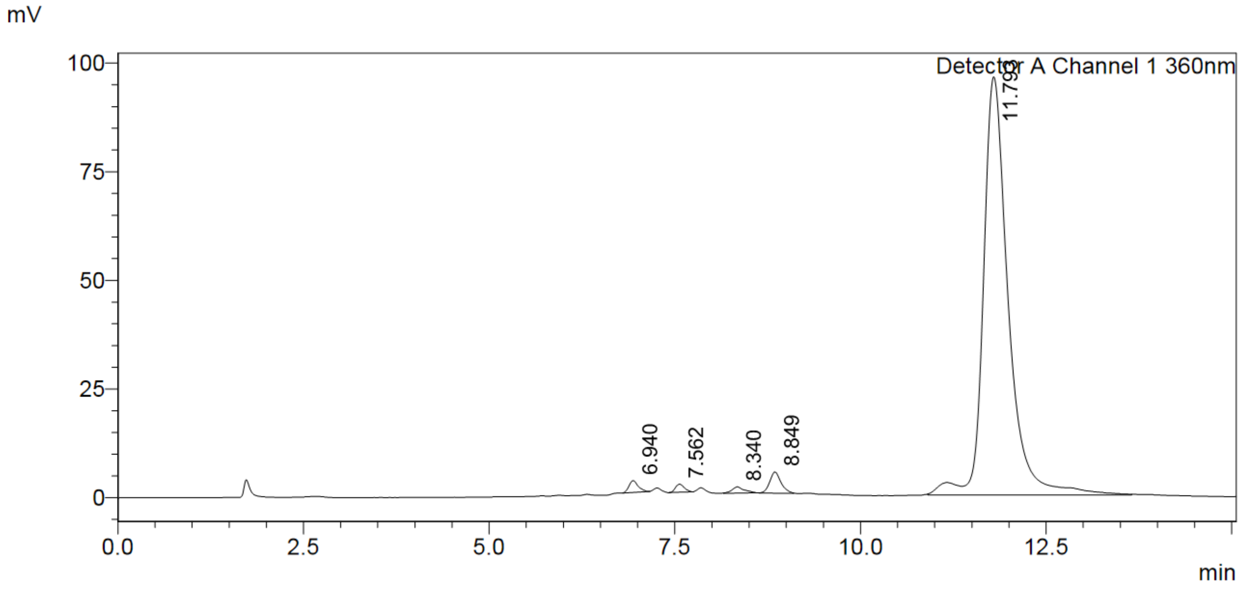

Figure S37. RP-HPLC trace showing product **21** at 11.8 min.

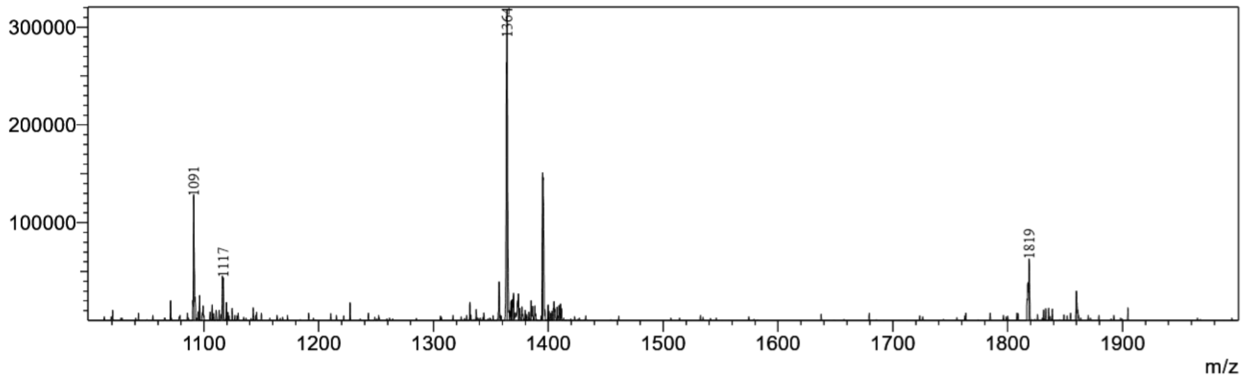

Figure S38. ESI-MS of **21**, expected m/z = 5469, observed m/z = $[M^+\text{-}H_2O+2H^++CH_3CN]^{+3}$ 1859, $[M^+\text{-}H_2O+2H^+]^{+3}$ 1819, $[M^+\text{-}H_2O+3H^++CH_3OH]^{+4}$ 1395, $[M^+\text{-}H_2O+3H^+]^{+4}$ 1364 $[M^+\text{-}H_2O+4H^+]^{+5}$ 1091 m/z.

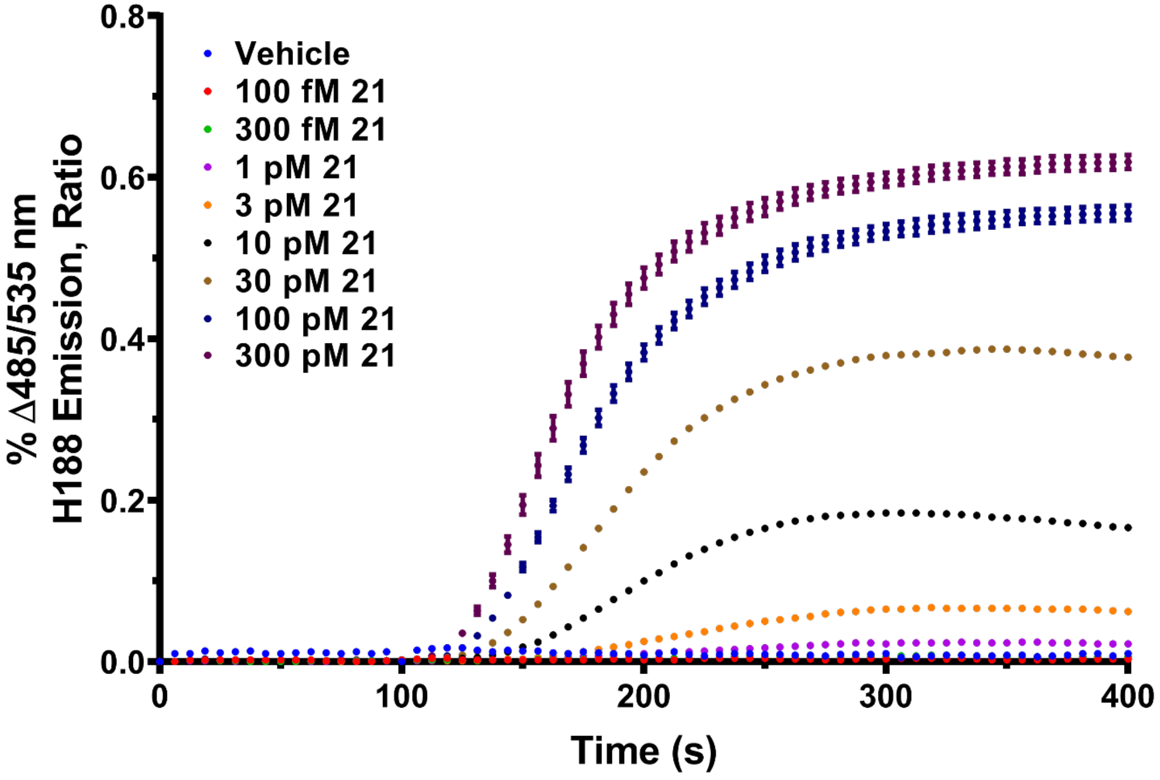

Figure S39. *In vitro* dose escalation study of **21** showing increase in cAMP levels in GLP-1R stably transfected HEK-293-H188 c20 cells.

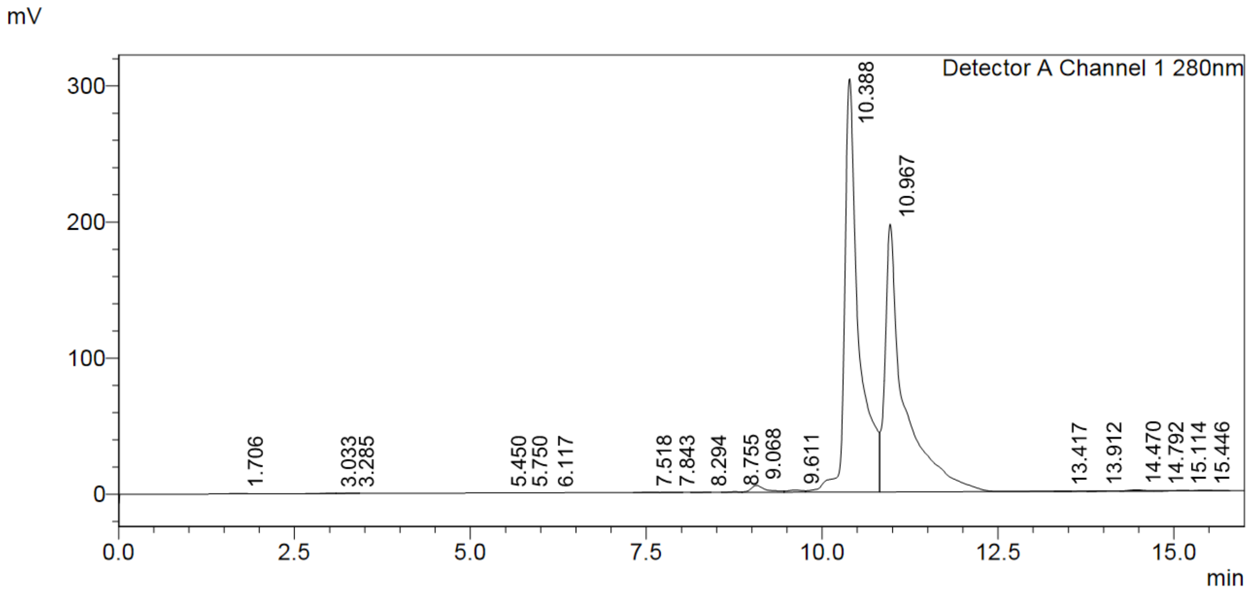

Figure S40. RP-HPLC trace showing the α- and β-isomer products of **6** at 10.4 and 11.0 min.

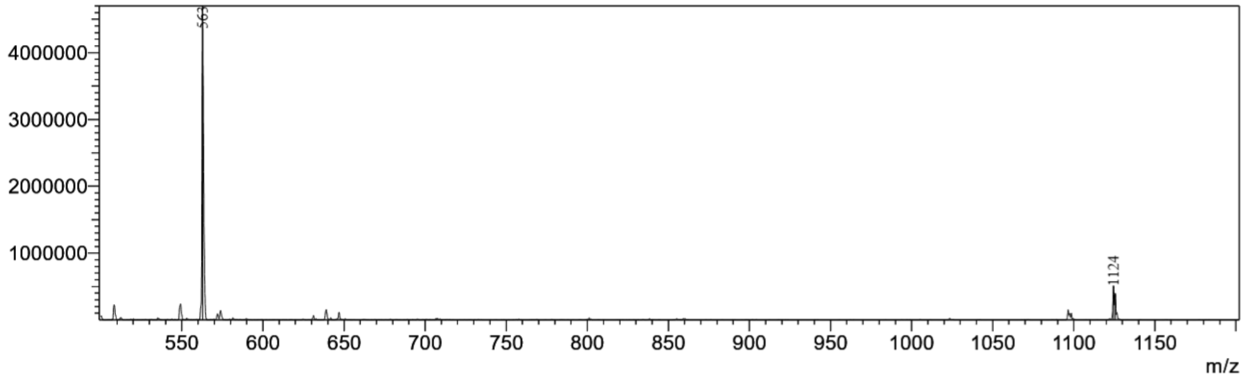

Figure S41. ESI-MS of **6**, expected m/z = 1143, observed m/z = $[M^{+}\text{-}H_2O]^{+1}$ 1124, $[M^{+}\text{-}H_2O+H^{+}]^{+2}$ 563 m/z.

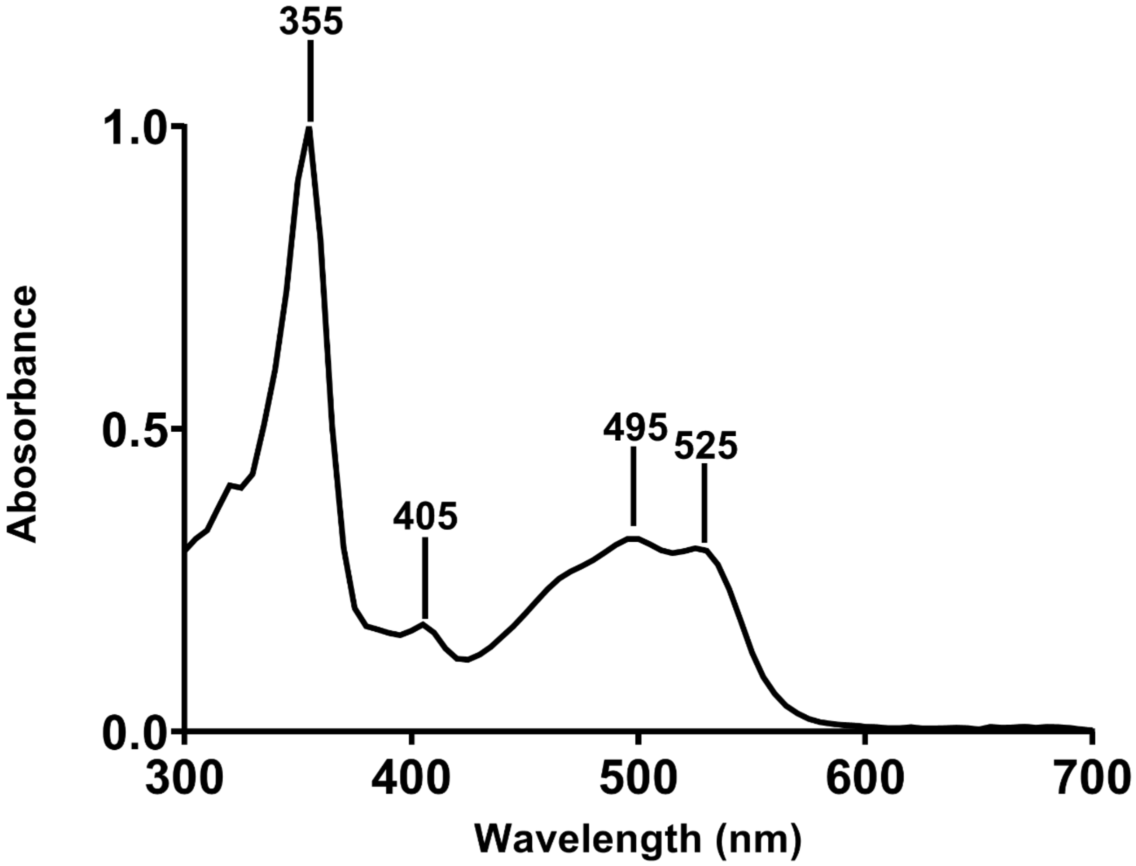

Figure S42. Electronic absorption spectra of **6** in water.

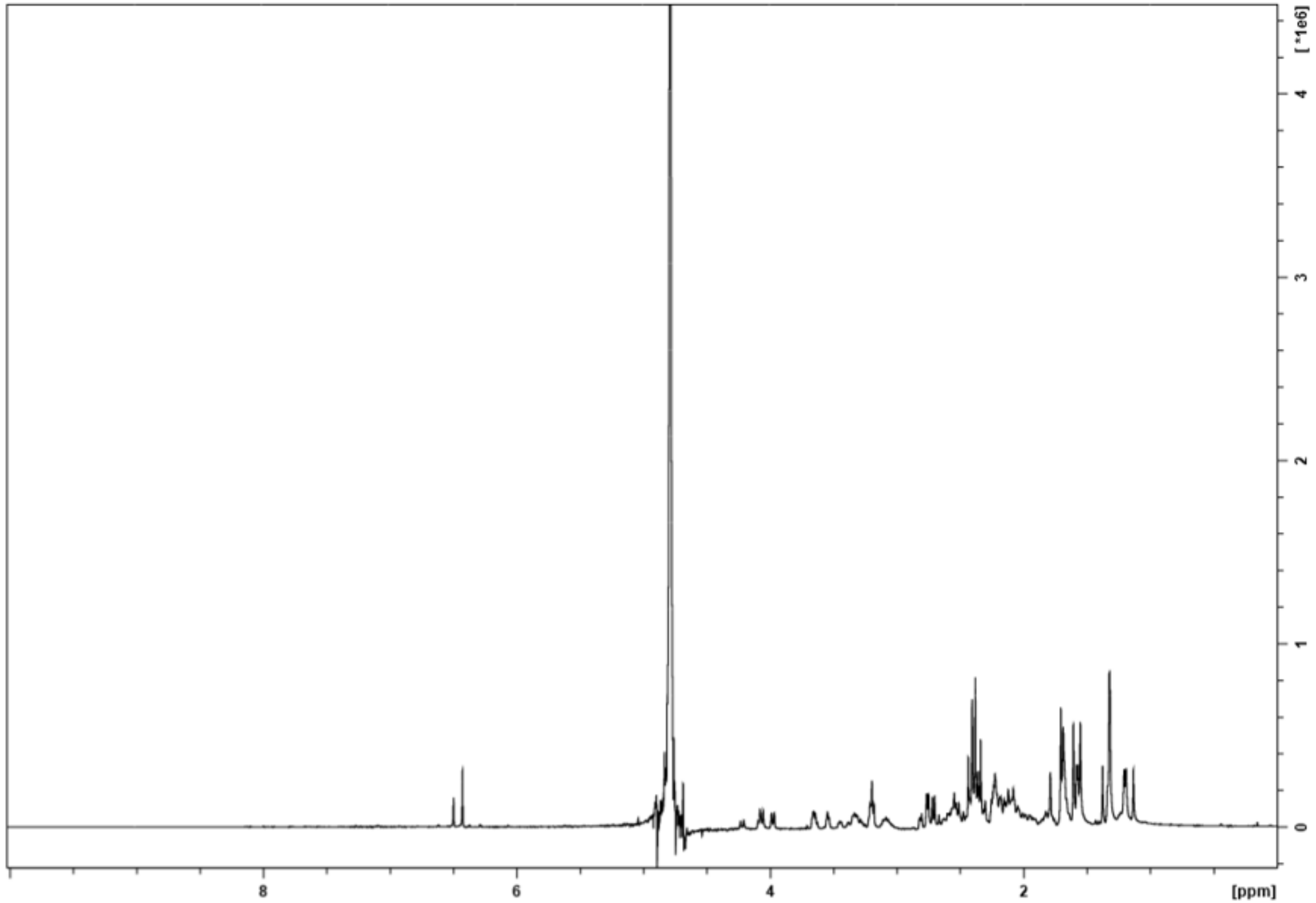

Figure S43. ^{1}H NMR of **6** (400 MHz, 298K, D_2O).

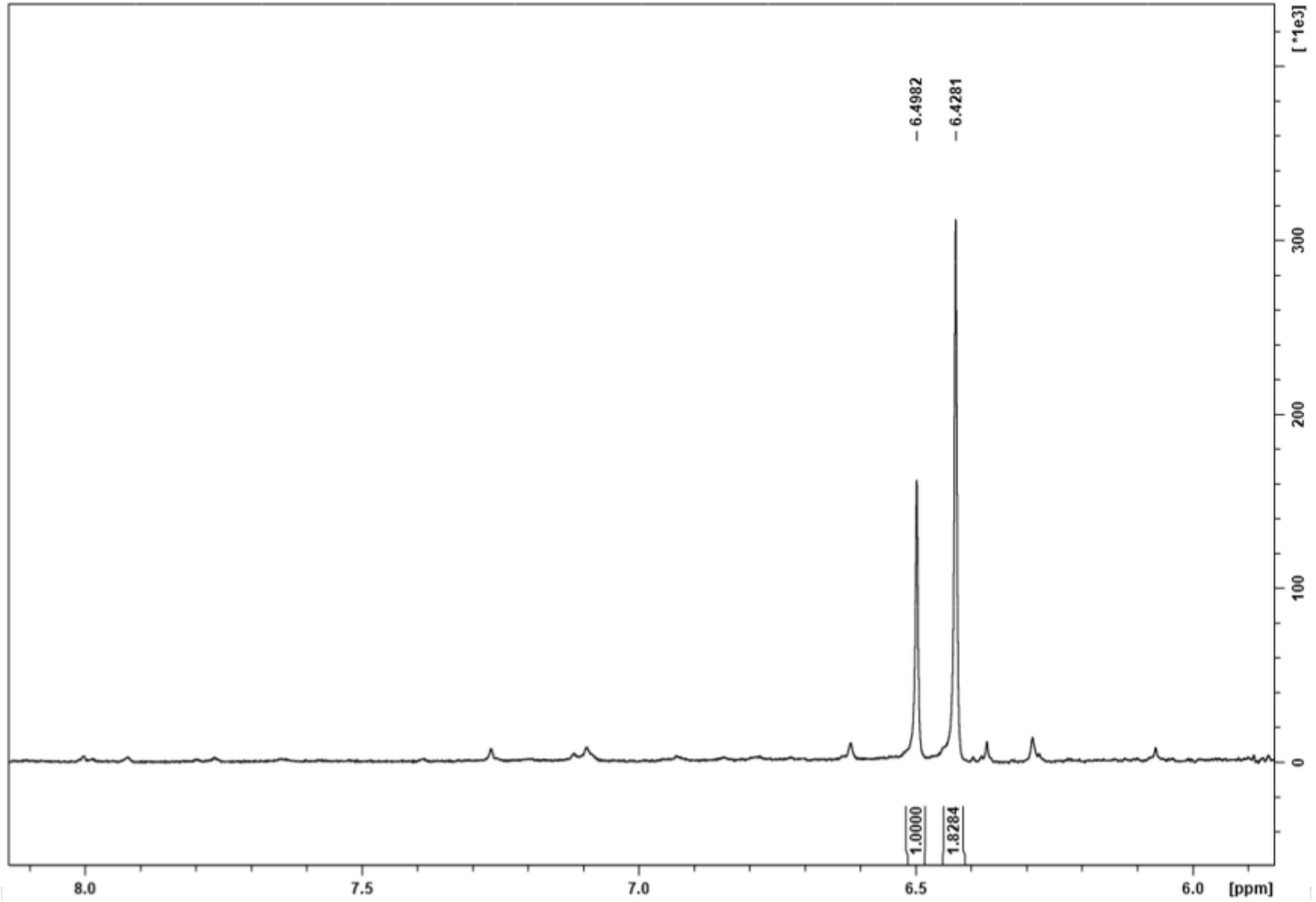

Figure S44. ^{1}H NMR of **6** (400 MHz, 298K, D_2O) (Aromatic). Characteristic signals (H19) of β- (6.50) and α- (6.43) aquo-isomers of **6** are observed.

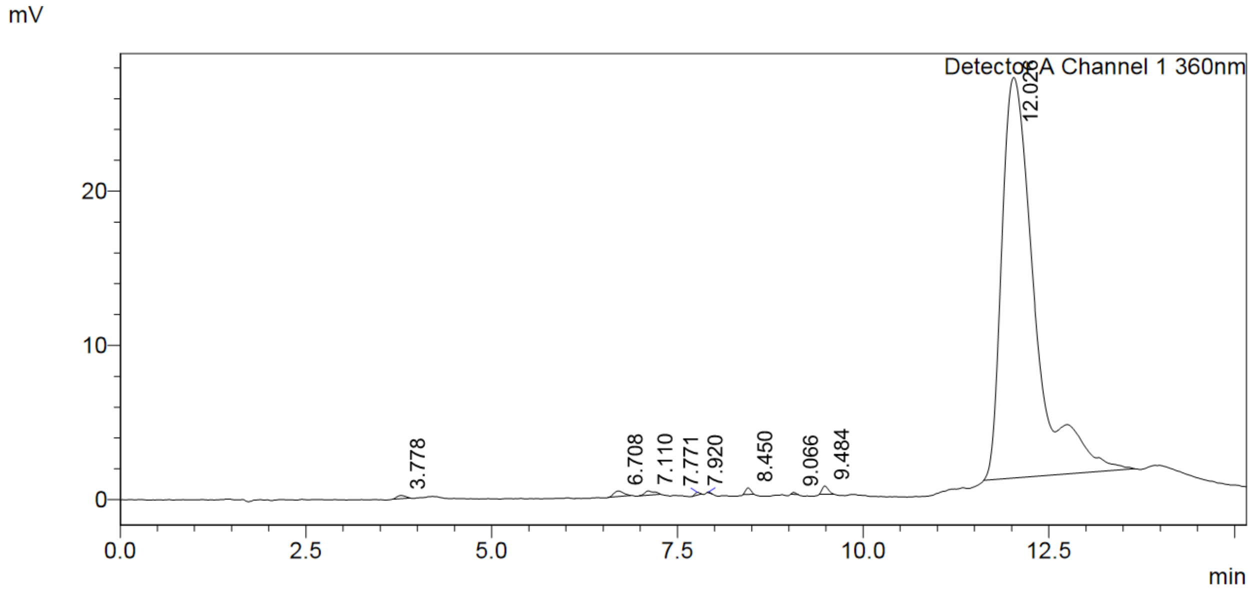

Figure S45. RP-HPLC trace showing product **14** at 12.0 min.

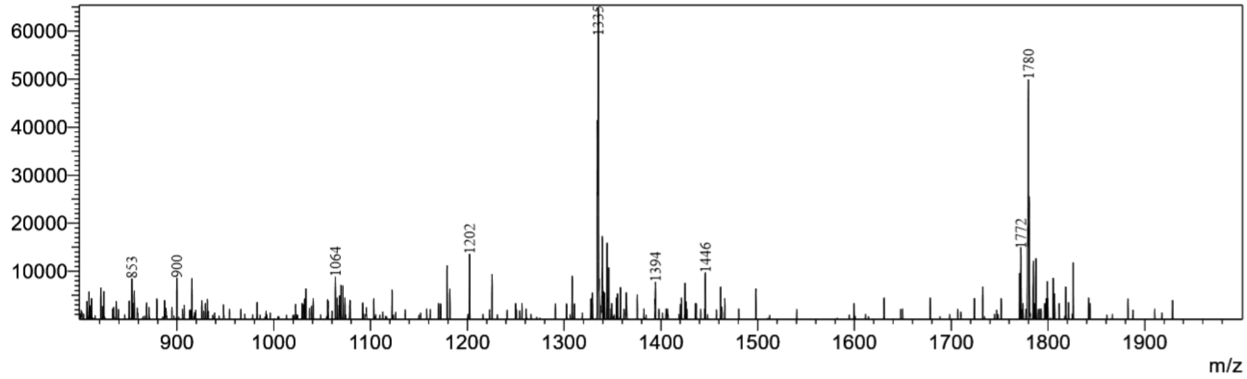

Figure S46. ESI-MS of **14**, expected m/z = 5355, observed m/z = $[M^{+}-H_2O+2H^{+}]^{+3}$ 1780, $[M^{+}-H_2O+3H^{+}]^{+4}$ 1335 m/z.

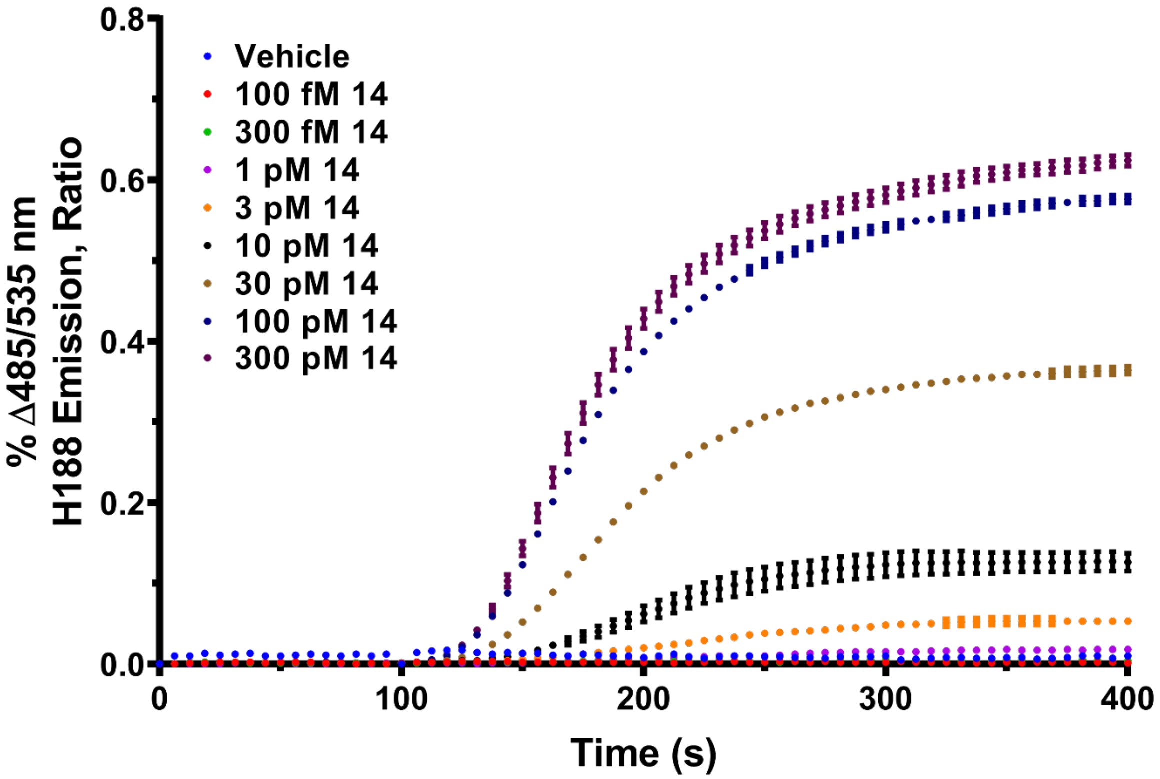

Figure S47. *In vitro* dose escalation study of **14** showing increase in cAMP levels in GLP-1R stably transfected HEK-293-H188 c20 cells.

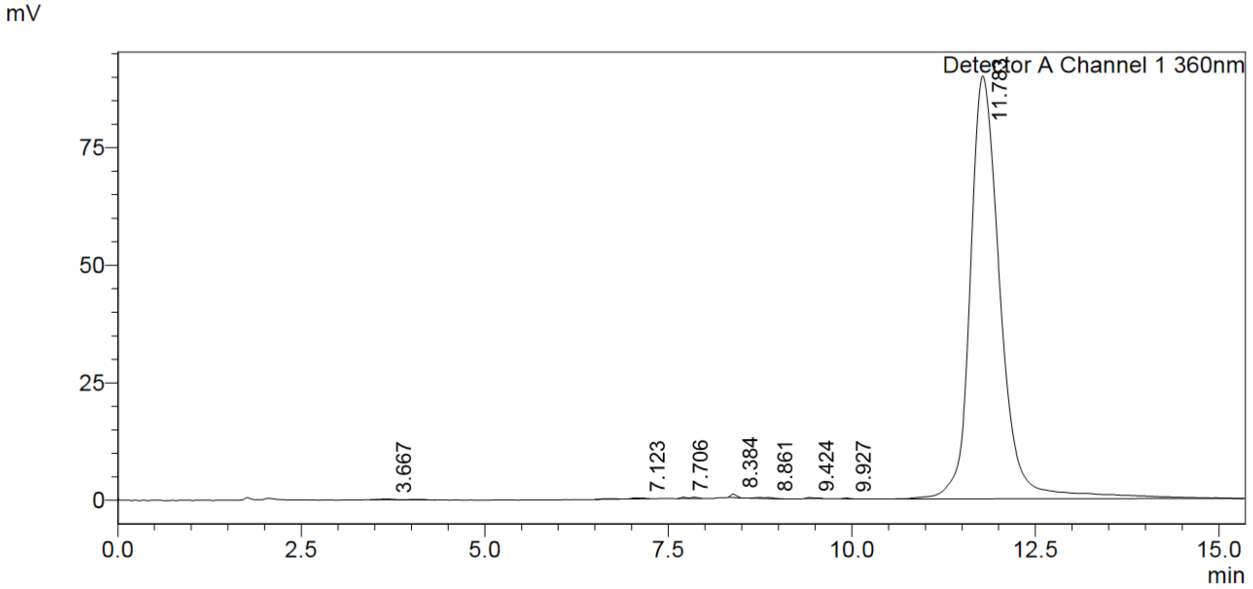

Figure S48. RP-HPLC trace showing product **22** at 11.8 min.

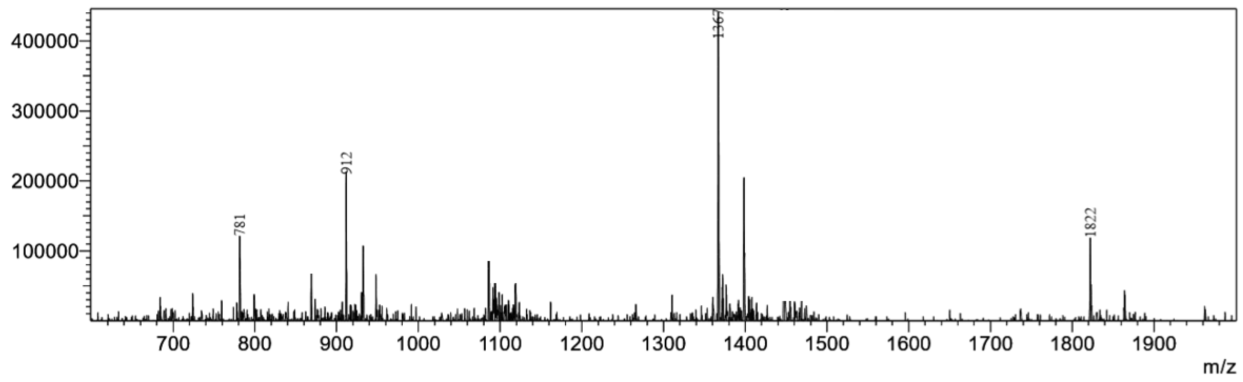

Figure S49. ESI-MS of **22**, expected m/z = 5483, observed m/z = $[M^{+}\text{-}H_2O+2H^{+}]^{+3}$ 1822, $[M^{+}\text{-}H_2O+3H^{+}]^{+4}$ 1367, $[M^{+}\text{-}H_2O+5H^{+}]^{+6}$ 912, $[M^{+}\text{-}H_2O+6H^{+}]^{+7}$ 781 m/z.

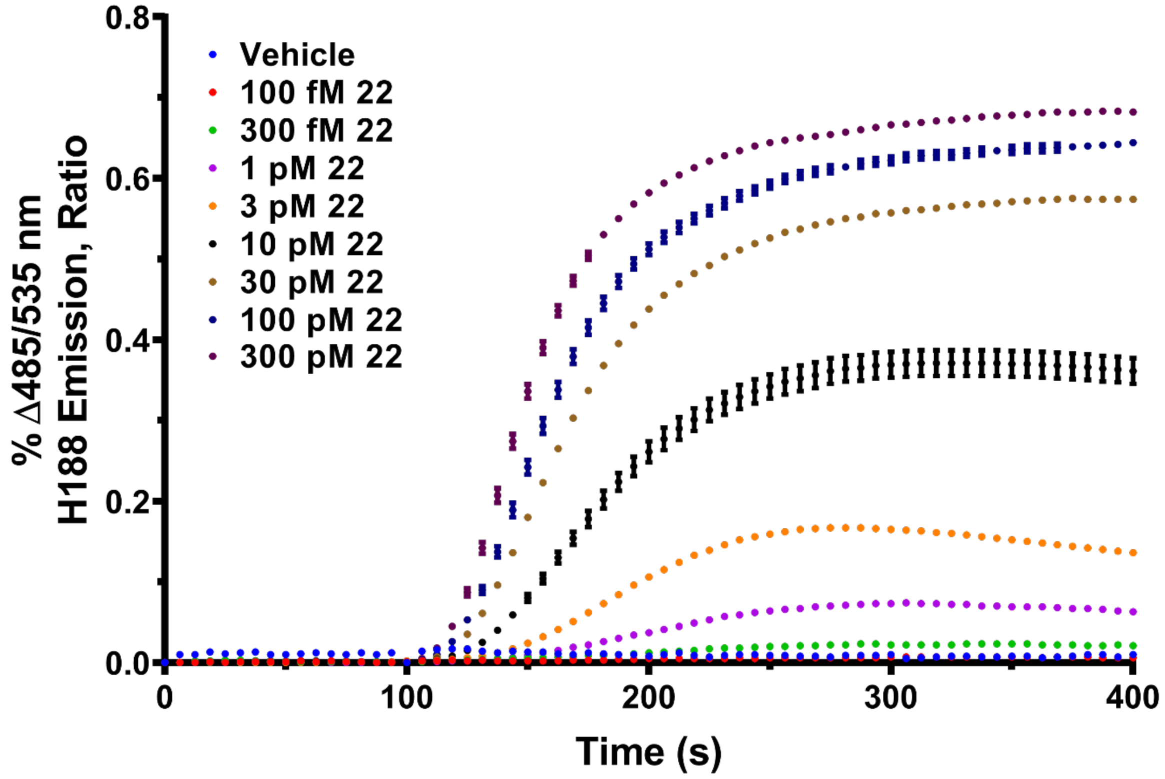

Figure S50. *In vitro* dose escalation study of **22** showing increase in cAMP levels in GLP-1R stably transfected HEK-293-H188 c20 cells.

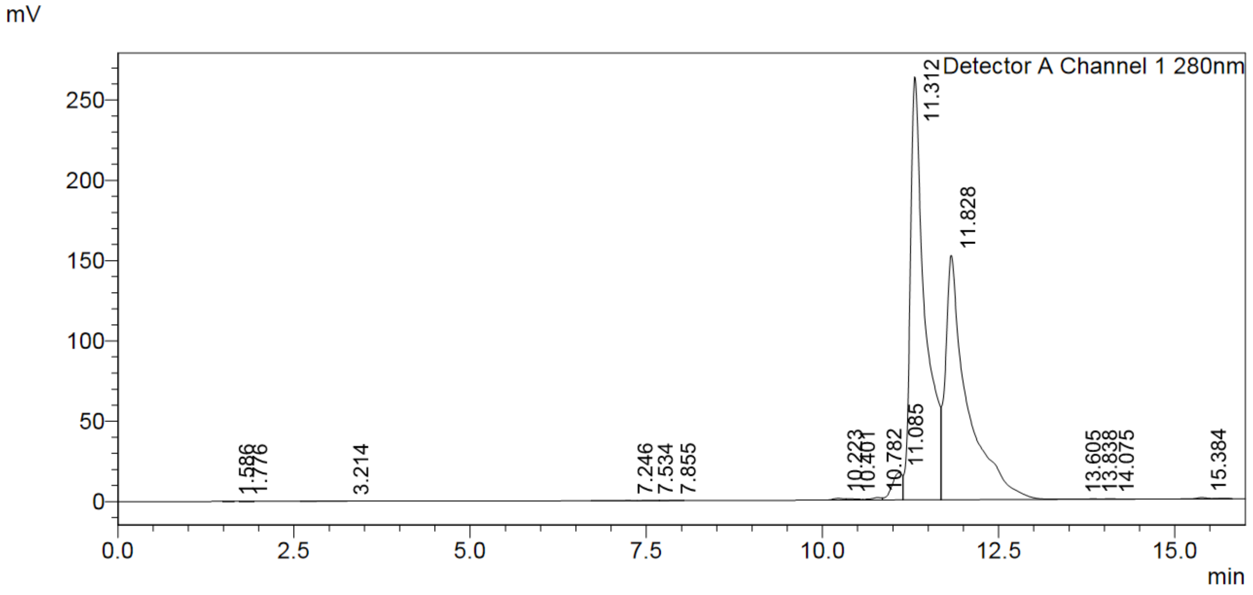

Figure S51. RP-HPLC trace showing the α- and β-isomer products of **7** 11.3 and 11.8 min.

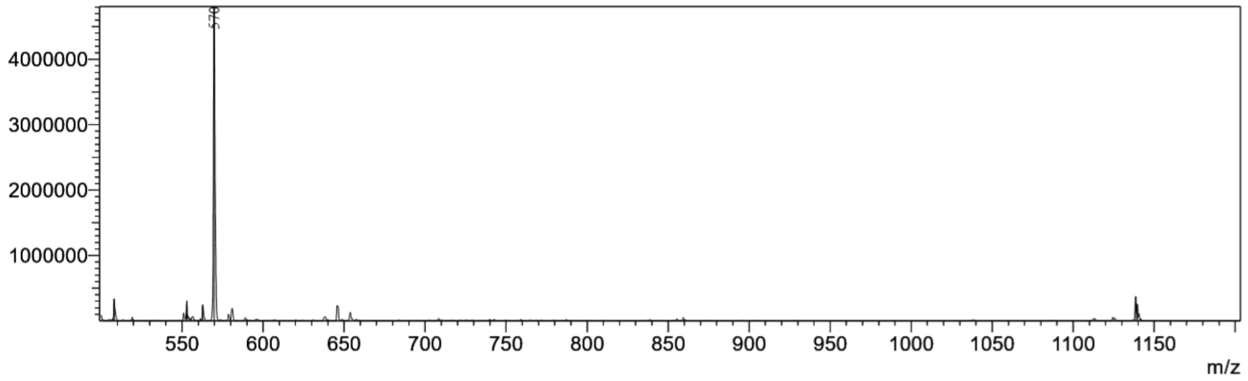

Figure S52. ESI-MS of **7**, expected m/z = 1157, observed m/z = $[M^{+}\text{-}H_2O]^{+1}$ 1139, $[M^{+}\text{-}H_2O+H^{+}]^{+2}$ 570 m/z.

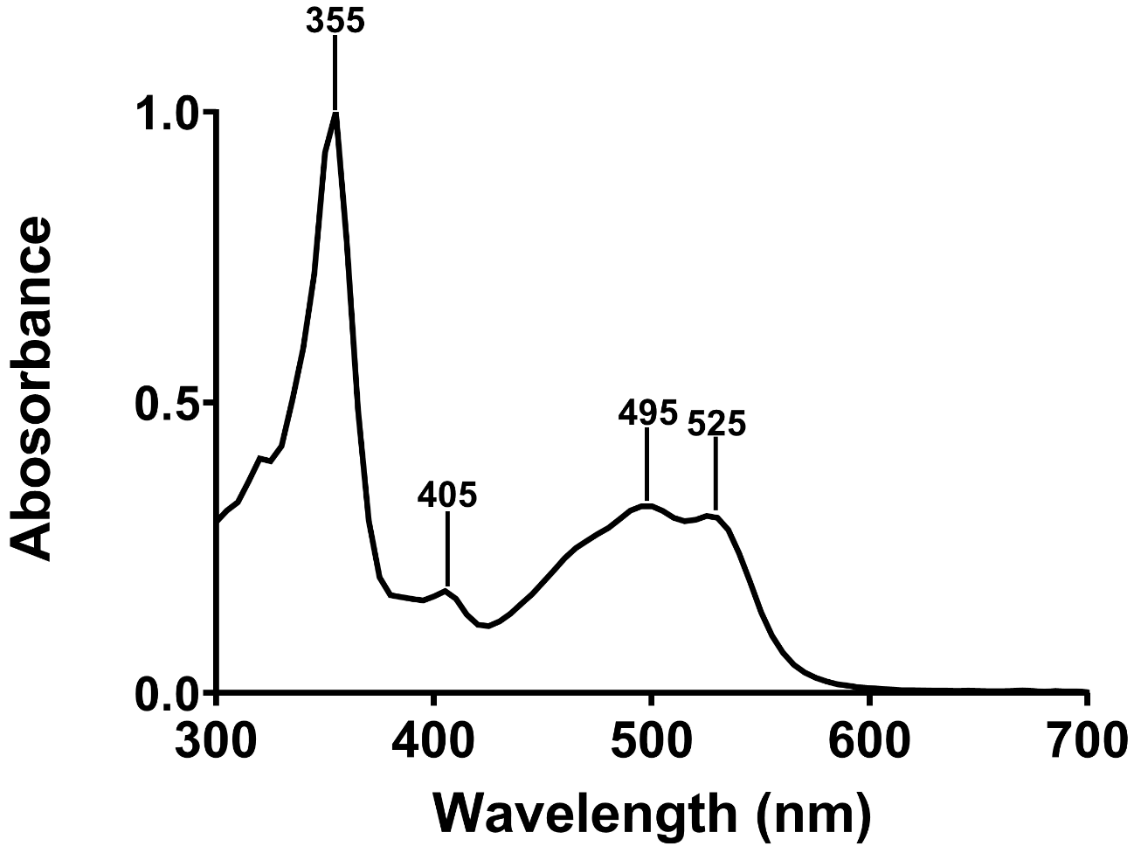

Figure S53. Electronic absorption spectra of **7** in water.

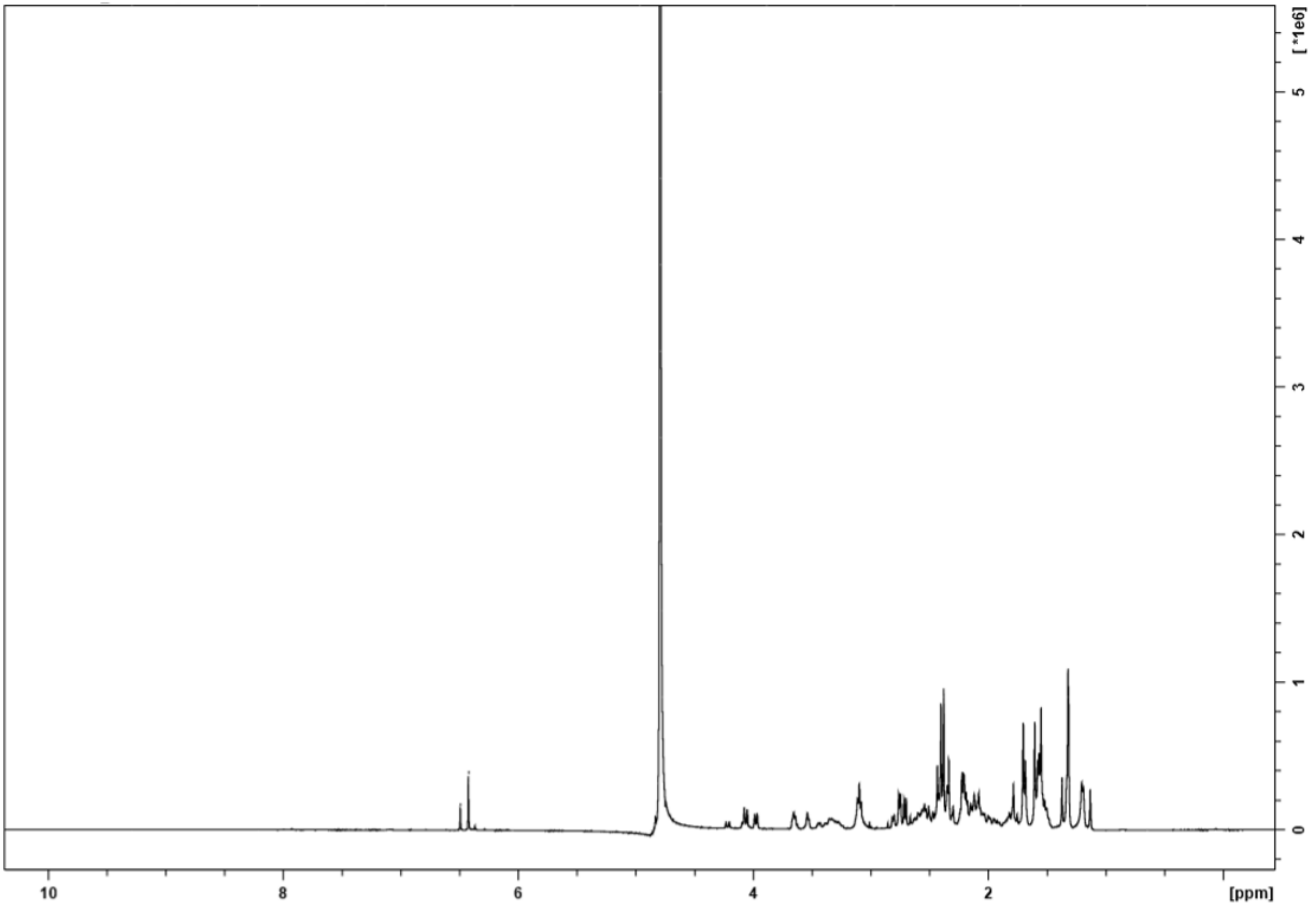

Figure S54: ^{1}H NMR of **7** (400 MHz, 298K, D_2O).

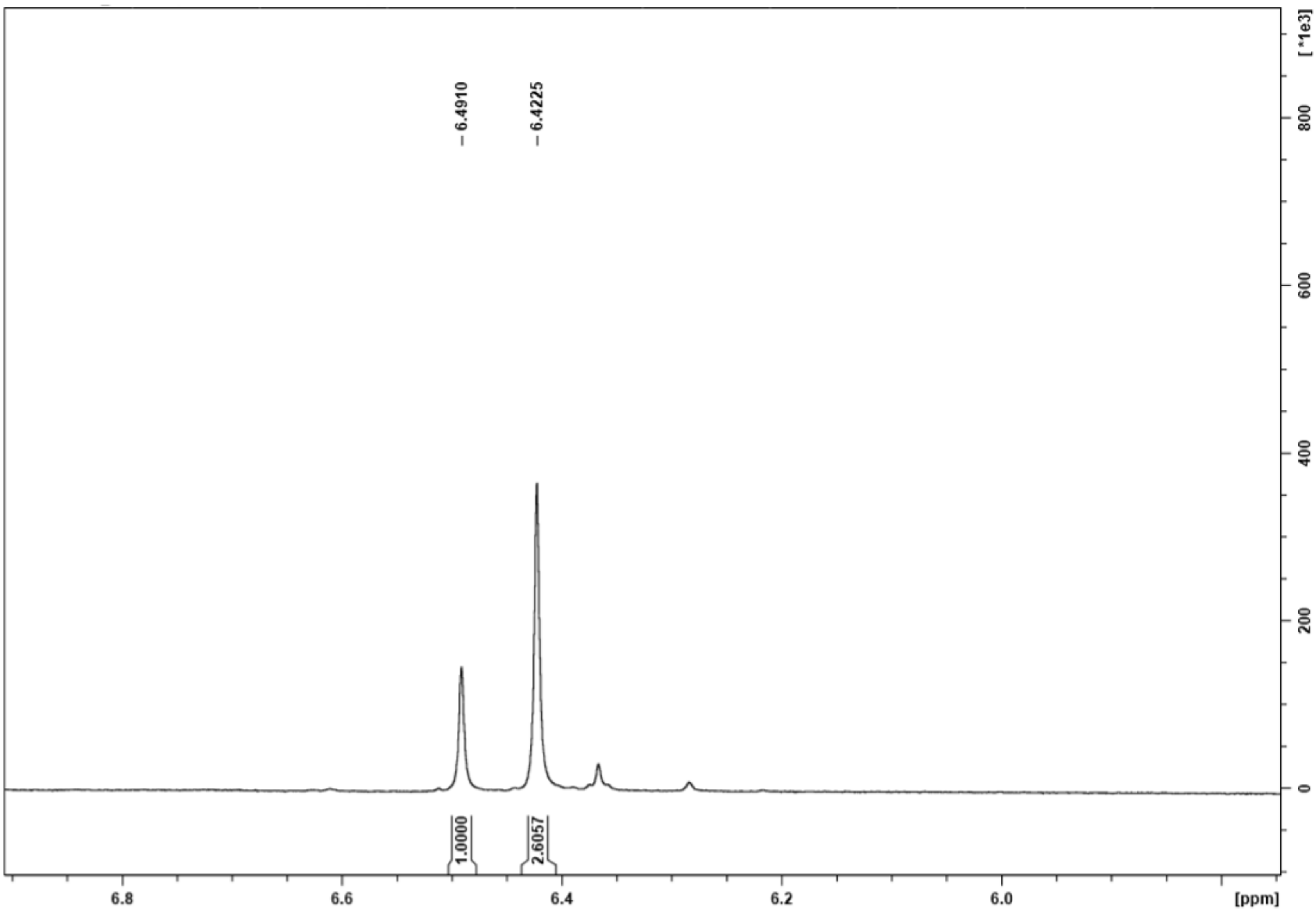

Figure S55. ^{1}H NMR of **7** (400 MHz, 298K, D_2O) (Aromatic). Characteristic signals (H19) of β- (6.49) and α- (6.42) aquo-isomers of **7** are observed.

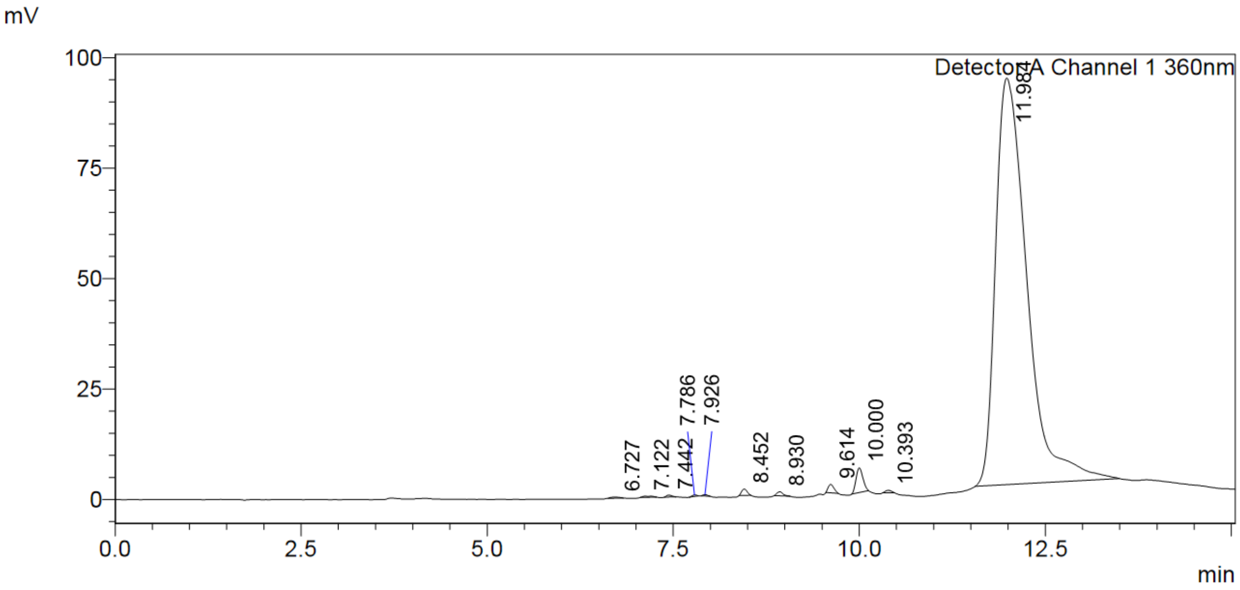

Figure S56. RP-HPLC trace showing product **15** at 12.0 min.

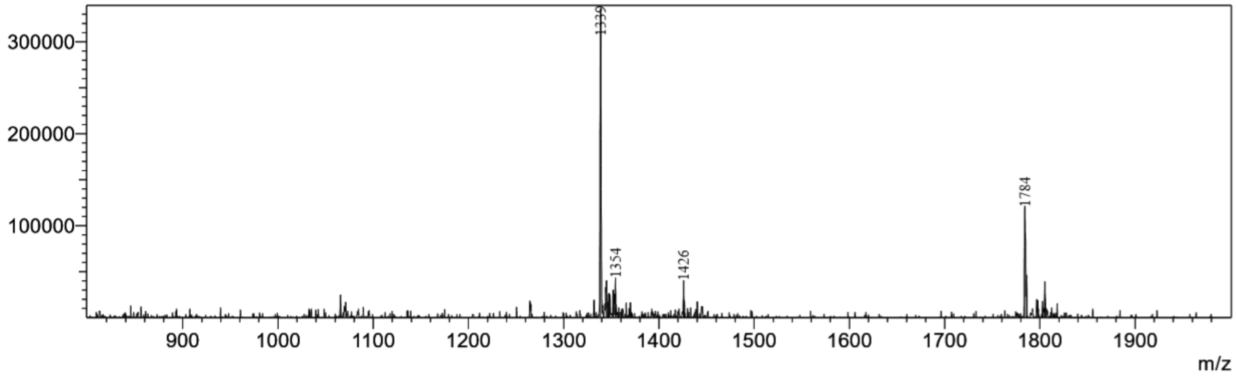

Figure S57. ESI-MS of **15**, expected m/z = 5369, observed m/z = $[M^{+}\text{-}H_2O+2H^{+}]^{+3}$ 1784, $[M^{+}\text{-}H_2O+3H^{+}]^{+4}$ 1339 m/z.

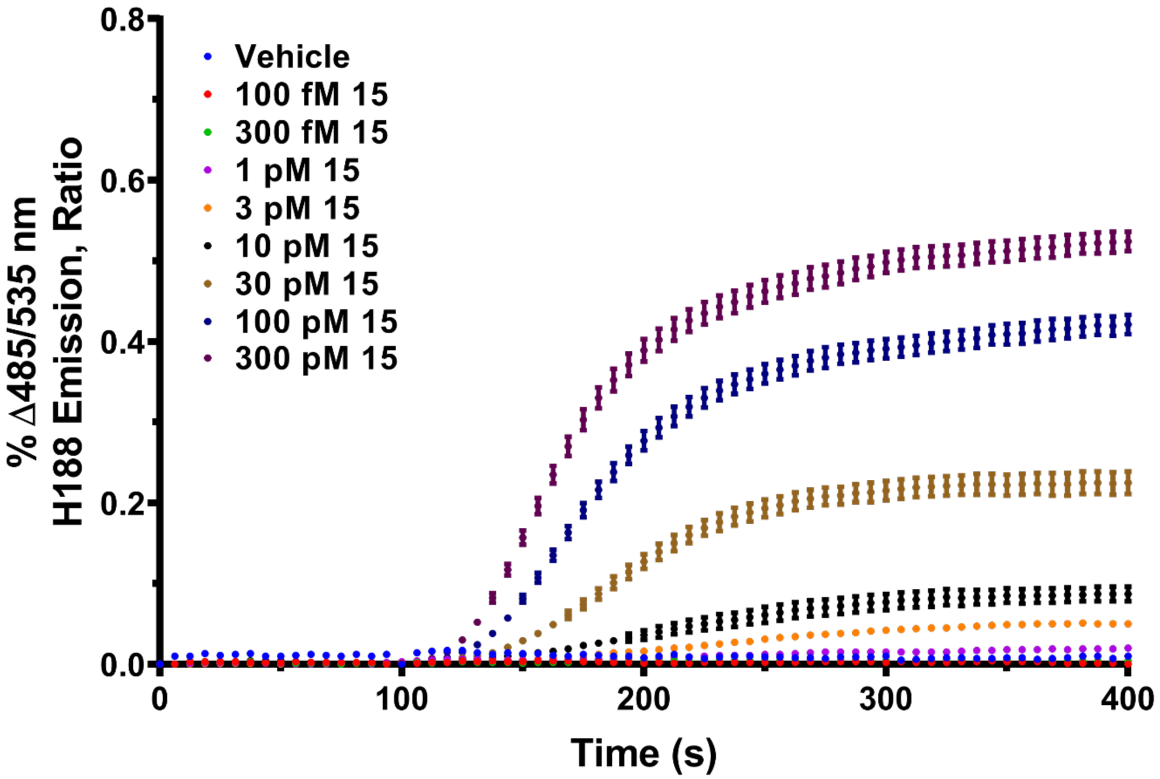

Figure S58. *In vitro* dose escalation study of **15** showing increase in cAMP levels in GLP-1R stably transfected HEK-293-H188 c20 cells.

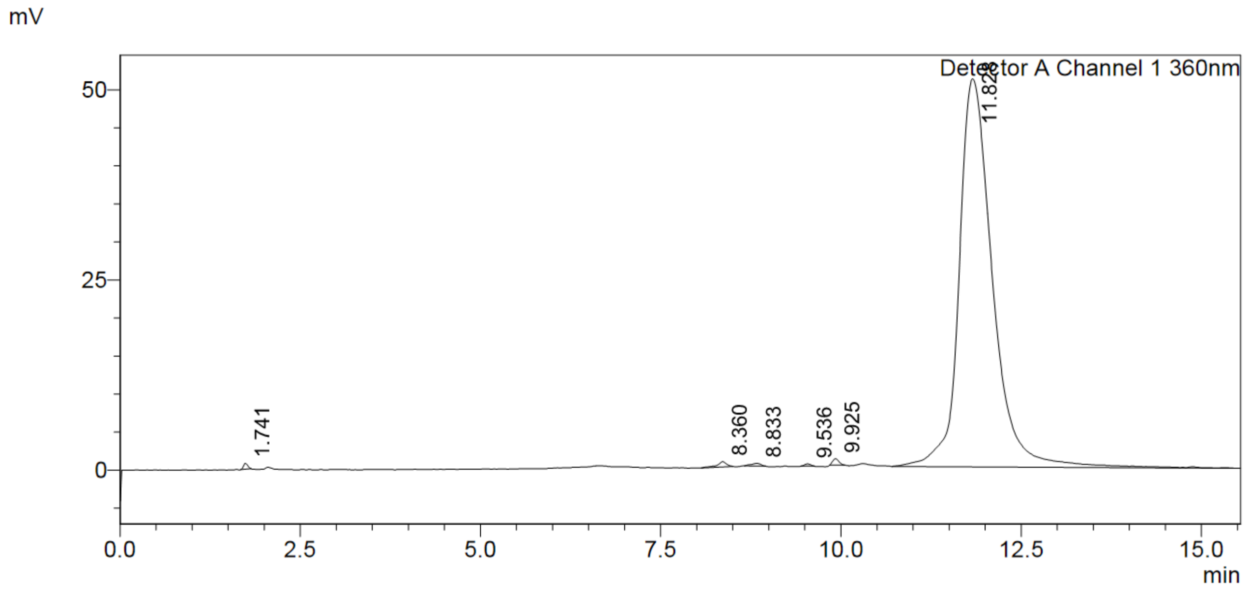

Figure S59. RP-HPLC trace showing product **23** at 11.8 min.

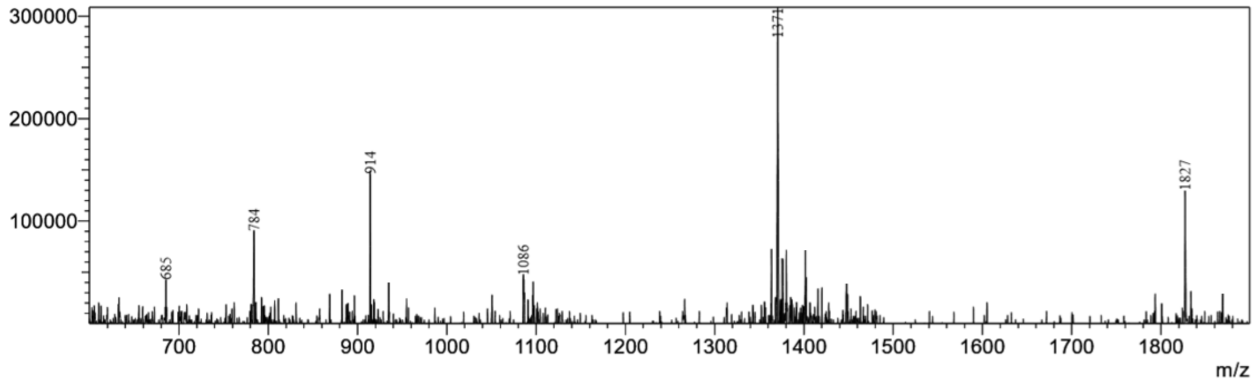

Figure S60. ESI-MS of **23**, expected m/z = 5497, observed m/z = $[M^{+}\text{-}H_2O+2H^{+}]^{+3}$ 1827, $[M^{+}\text{-}H_2O+3H^{+}]^{+4}$ 1371, $[M^{+}\text{-}H_2O+5H^{+}]^{+6}$ 914, $[M^{+}\text{-}H_2O+6H^{+}]^{+7}$ 784, $[M^{+}\text{-}H_2O+7H^{+}]^{+8}$ 685 m/z.

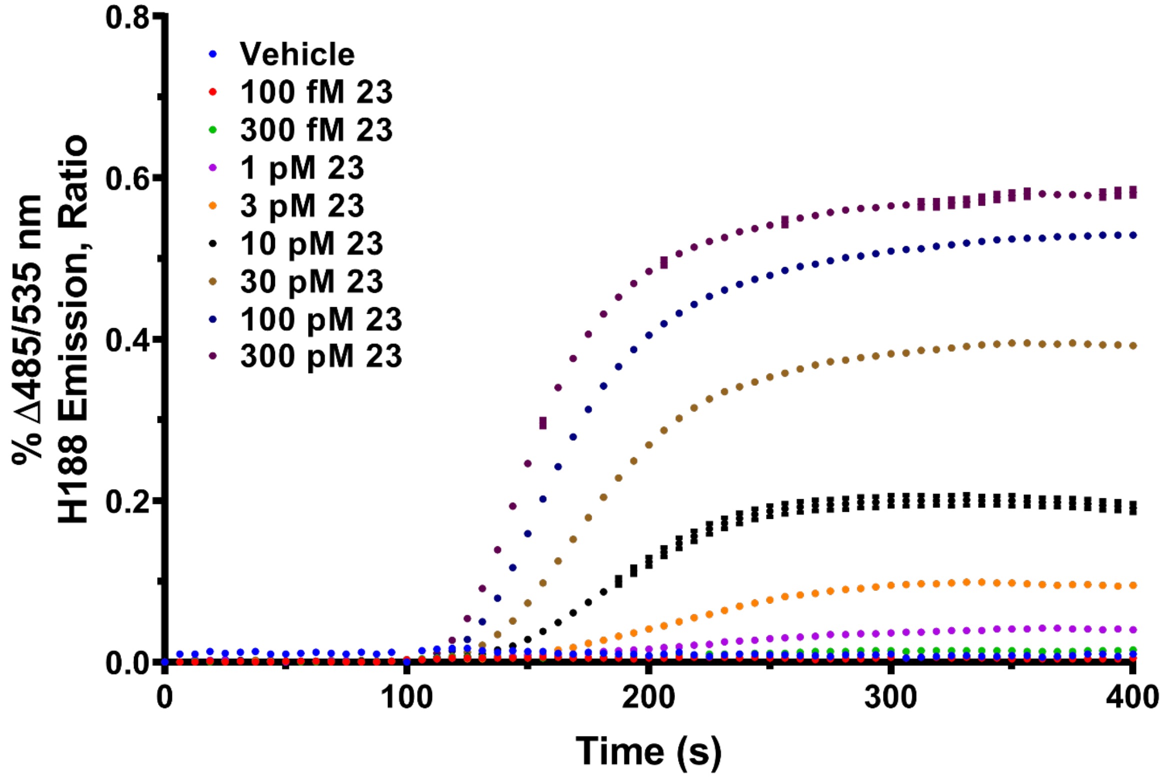

Figure S61. *In vitro* dose escalation study of **23** showing increase in cAMP levels in GLP-1R stably transfected HEK-293-H188 c20 cells.

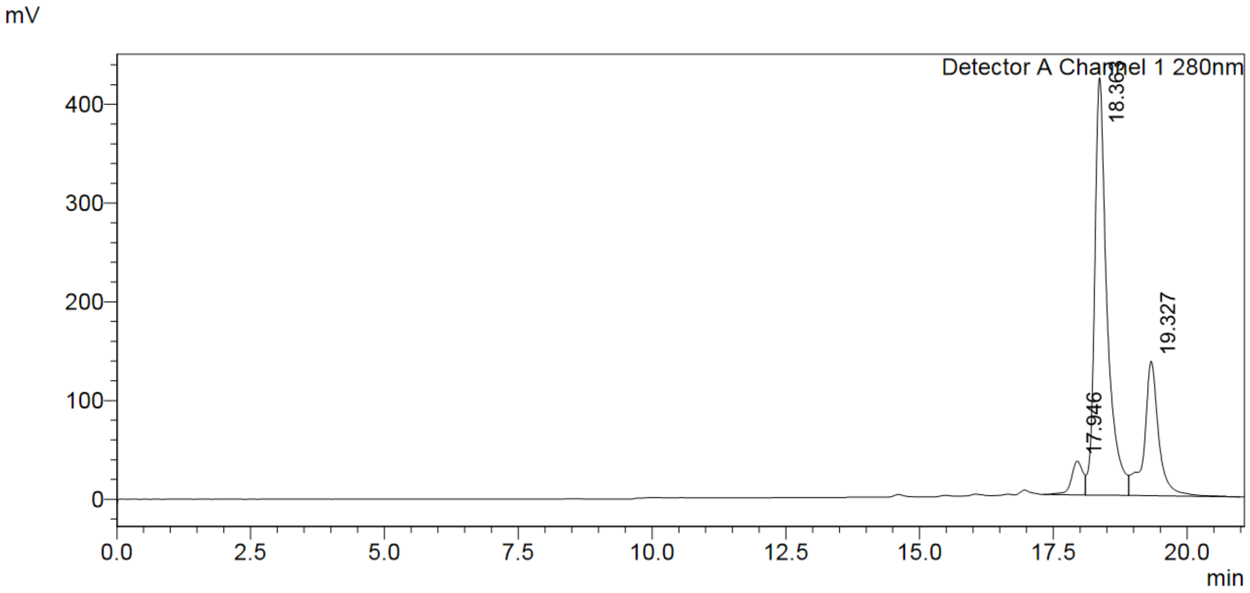

Figure S62. RP-HPLC trace showing the α- and β-isomer products of **8** at 18.4 and 19.3 min.

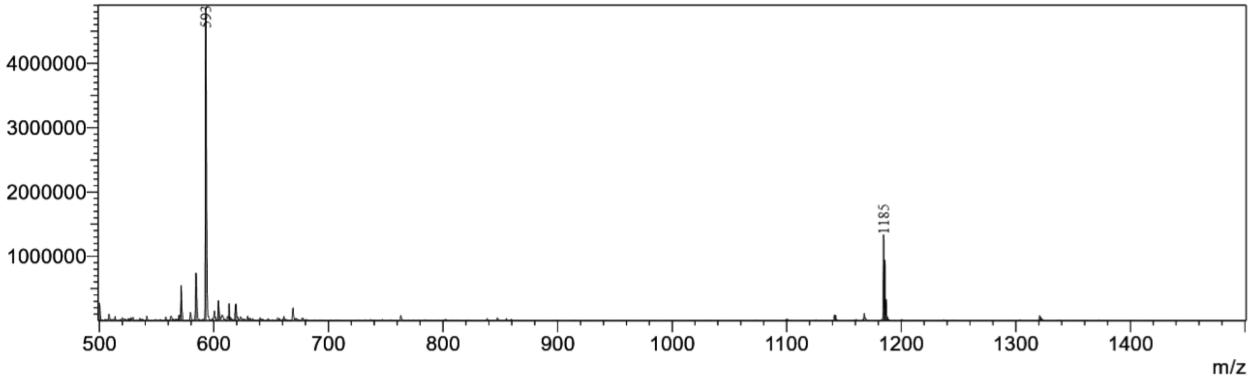

Figure S63. ESI-MS of **8**, expected m/z = 1204, observed m/z = $[M^{+}\text{-}H_2O]^{+1}$ 1185, $[M^{+}\text{-}H_2O+H^{+}]^{+2}$ 593 m/z.

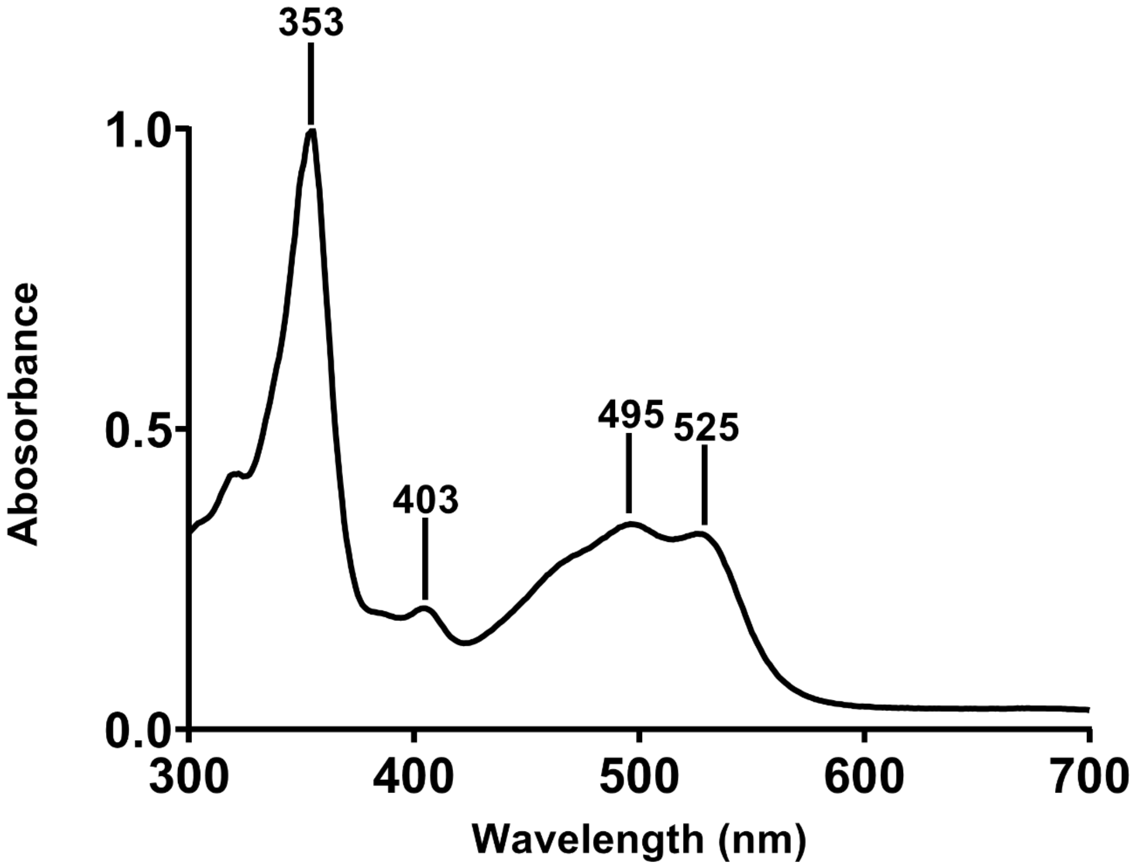

Figure S64. Electronic absorption spectra of **8** in water.

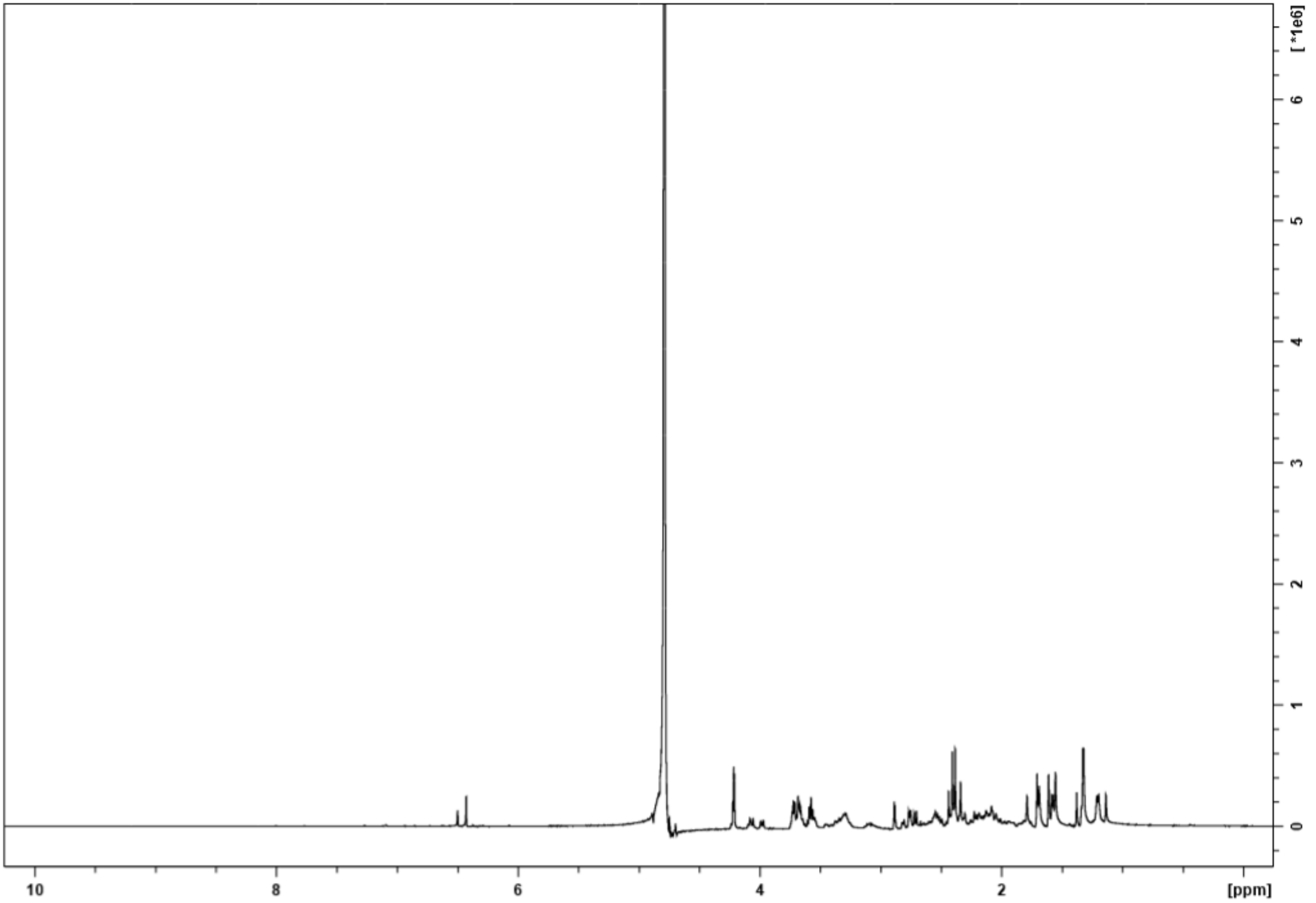

Figure S65. ^{1}H NMR of **8** (400 MHz, 298K, D_2O).

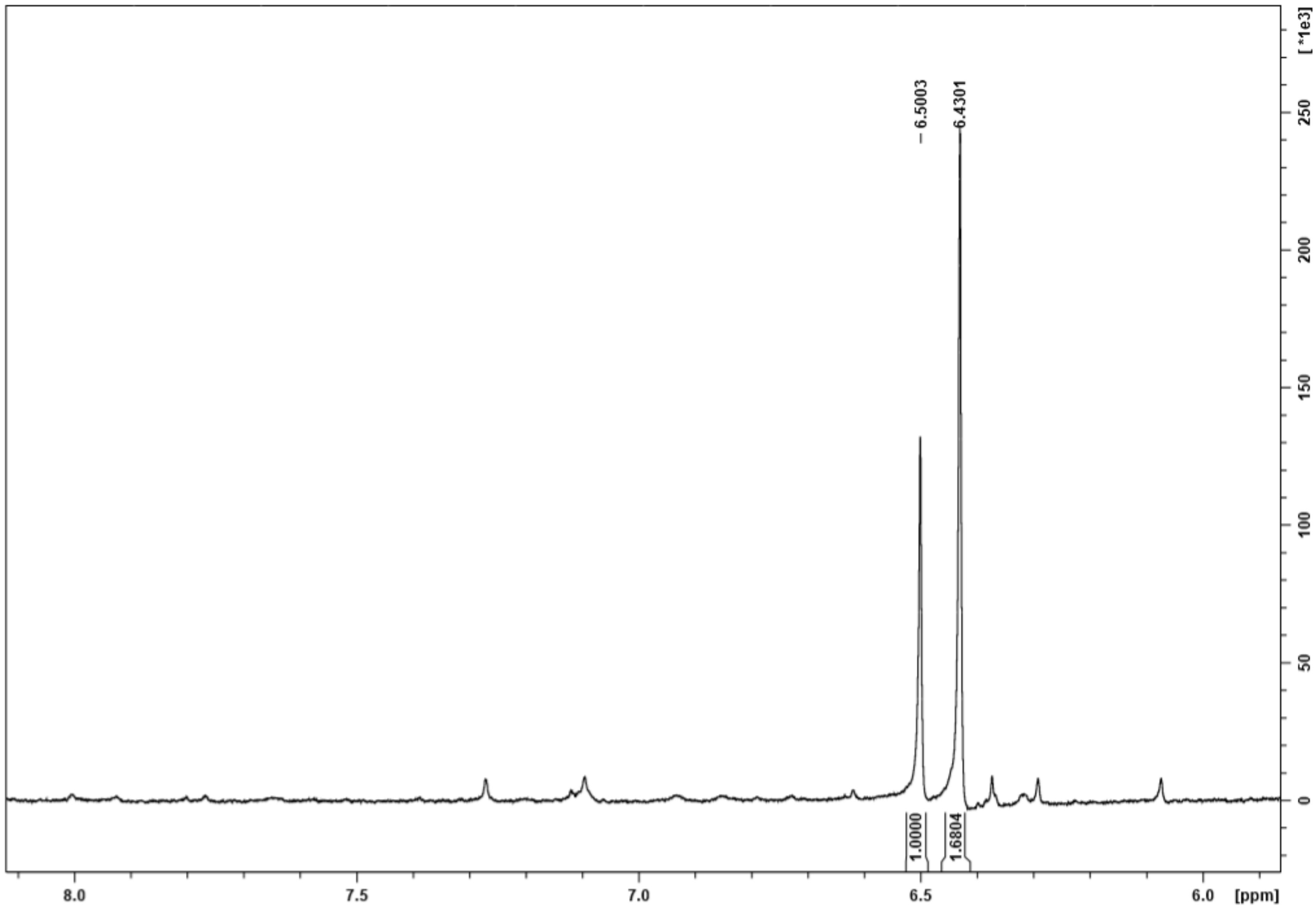

Figure S66. ^{1}H NMR of **8** (400 MHz, 298K, D_2O) (Aromatic). Characteristic signals (H19) of β- (6.50) and α- (6.43) aquo-isomers of **8** are observed.

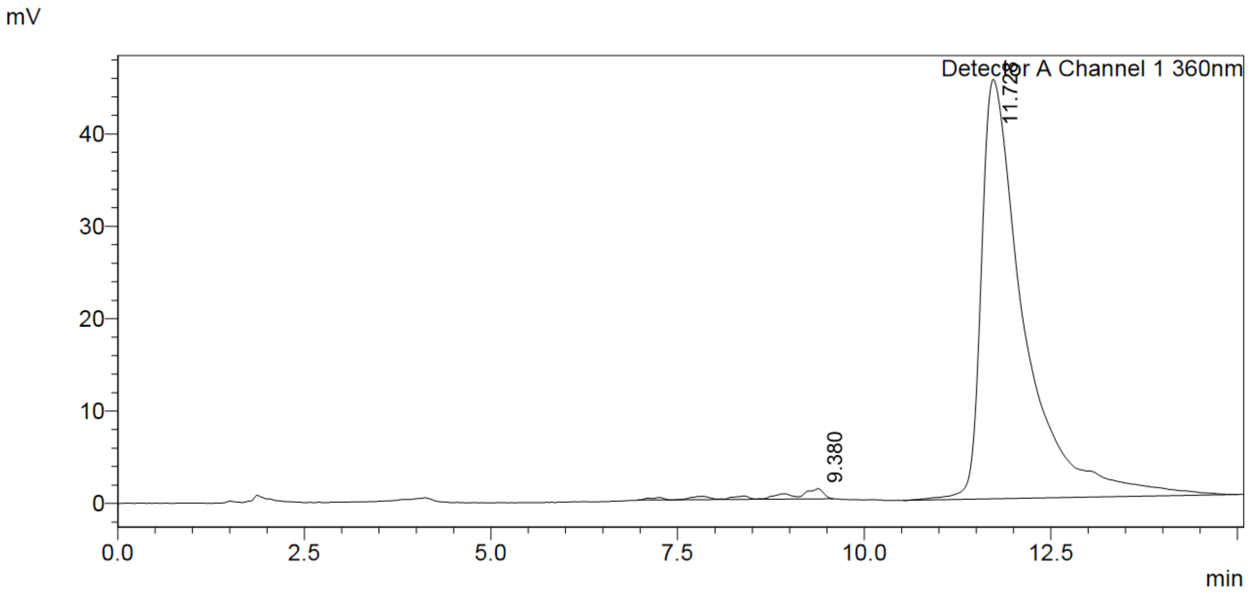

Figure S67. RP-HPLC trace showing product **16** at 11.7 min.

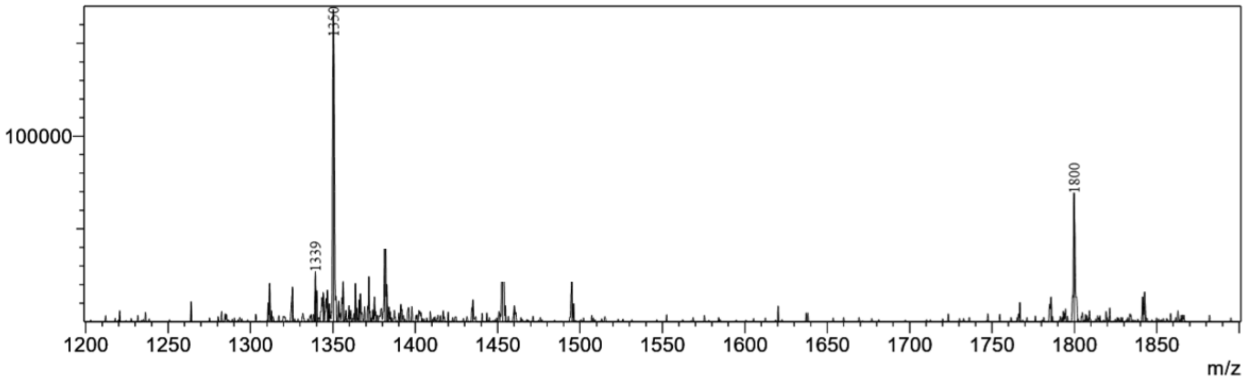

Figure S68. ESI-MS of **16**, expected m/z = 5416, observed m/z = $[M^{+}\text{-}H_2O+2H^{+}]^{+3}$ 1800, $[M^{+}\text{-}H_2O+3H^{+}]^{+4}$ 1350 m/z.

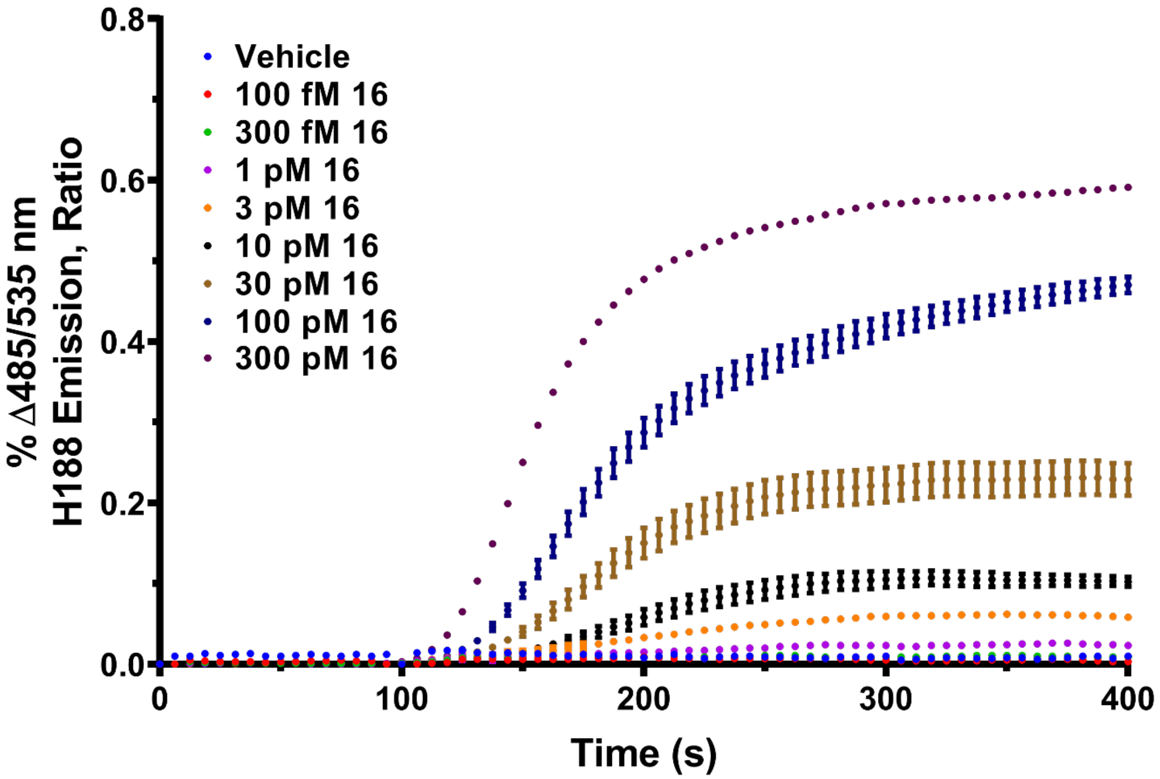

Figure S69. *In vitro* dose escalation study of **16** showing increase in cAMP levels in GLP-1R stably transfected HEK-293-H188 c20 cells.

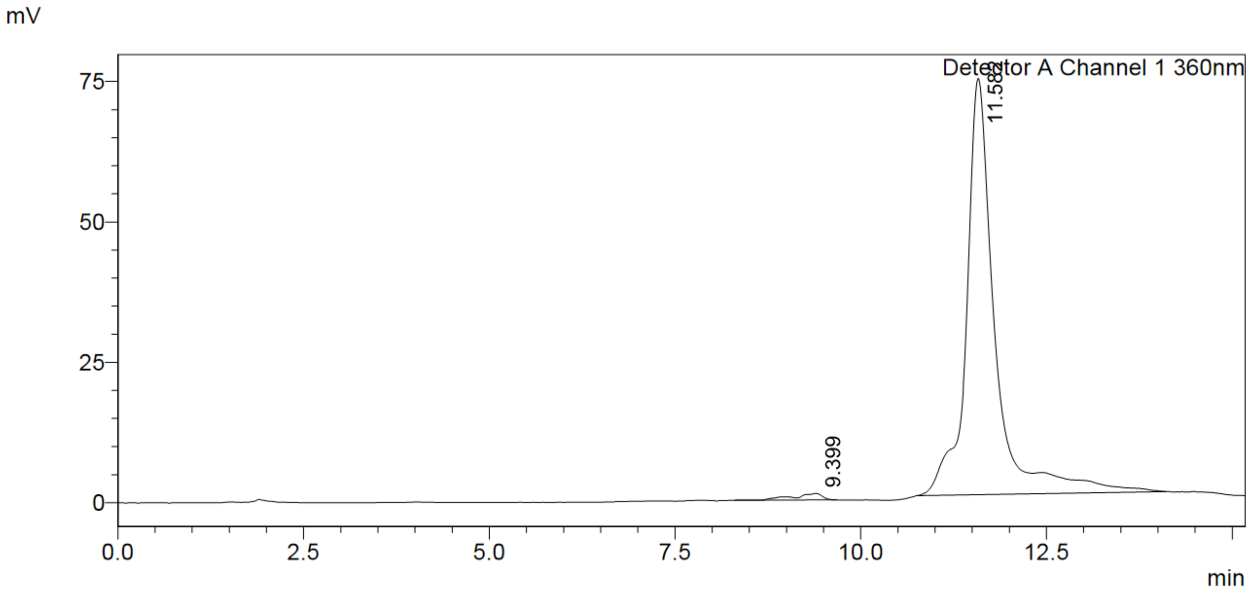

Figure S70. RP-HPLC trace showing product **24** at 11.6 min.

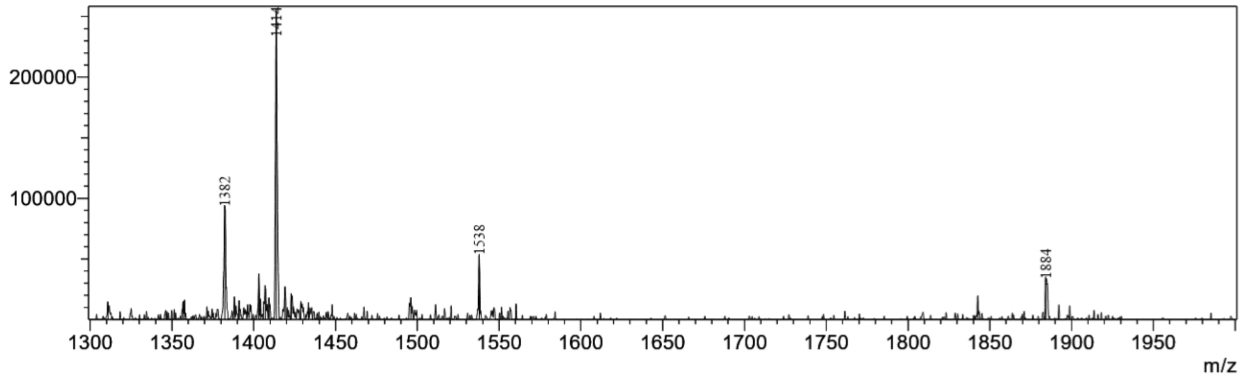

Figure S71. ESI-MS of **24**, expected m/z = 5544, observed m/z = $[M^{+}\text{-}H_2O+2H^{+}+CH_3CN]^{+3}$ 1884, $[M^{+}\text{-}H_2O+3H^{+}+CH_3OH]^{+4}$ 1414, $[M^{+}\text{-}H_2O+3H^{+}]^{+4}$ 1382 m/z.

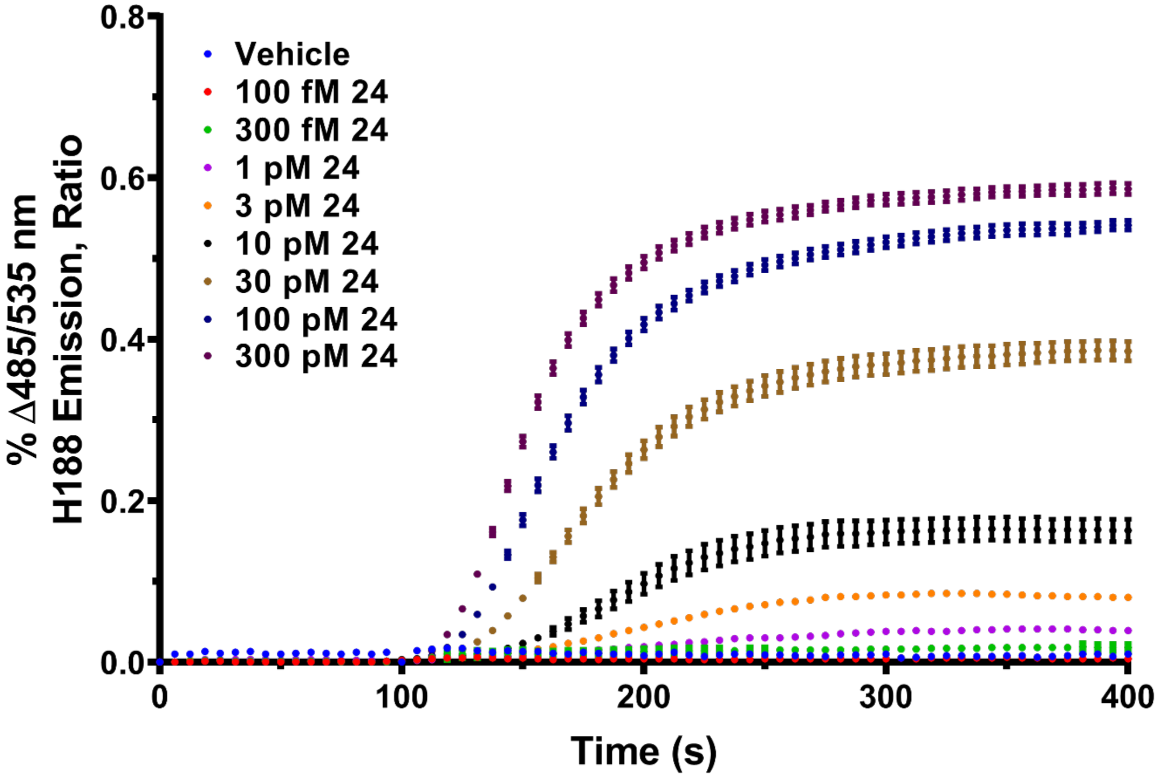

Figure S72. *In vitro* dose escalation study of **24** showing increase in cAMP levels in GLP-1R stably transfected HEK-293-H188 c20 cells.

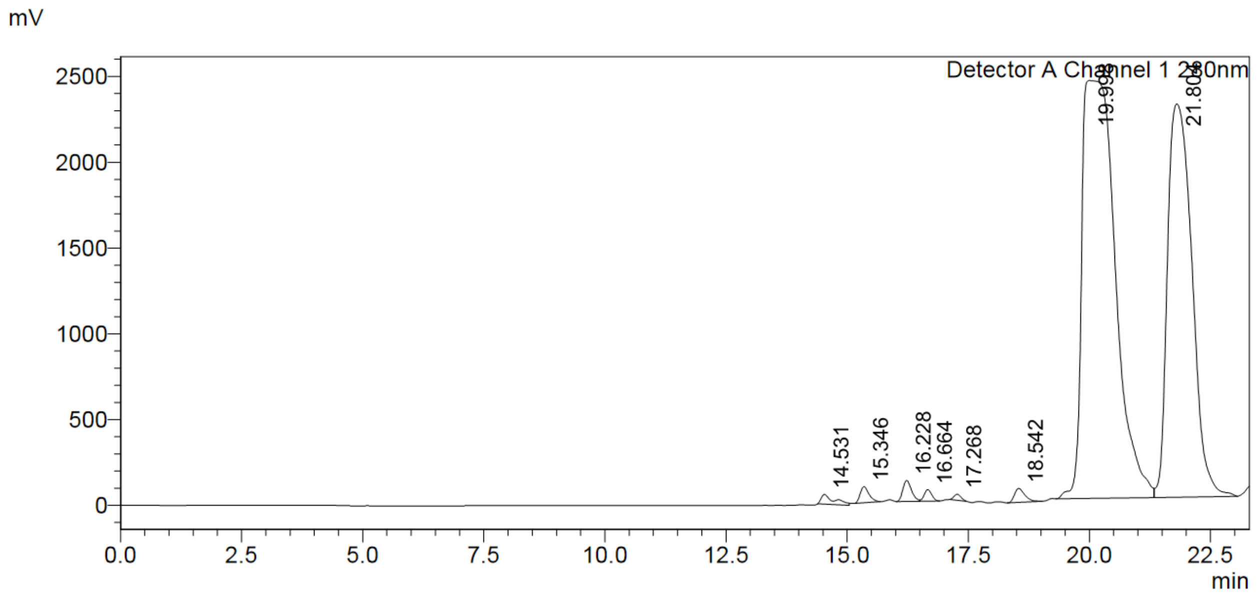

Figure S73. RP-HPLC trace showing the α- and β-isomer products of **9** at 20.0 and 21.8 min.

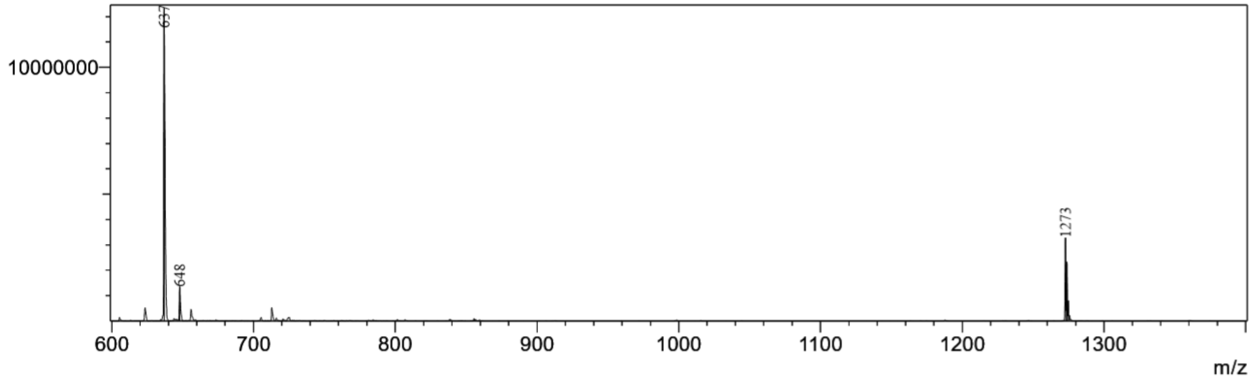

Figure S74. ESI-MS of **9**, expected m/z = 1292, observed m/z = $[M^{+}\text{-}H_2O]^{+1}$ 1273, $[M^{+}\text{-}H_2O\text{+}H^{+}]^{+2}$ 637 m/z.

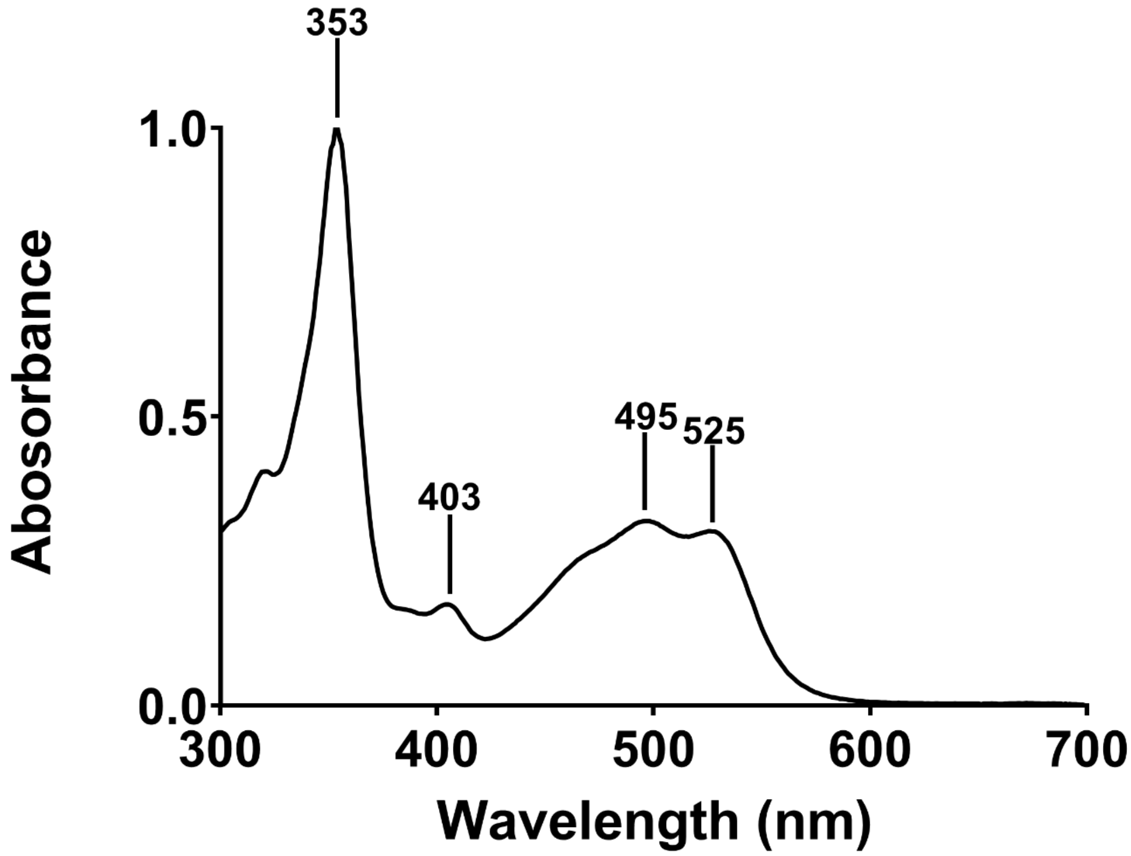

Figure S75. Electronic absorption spectra of **9** in water.

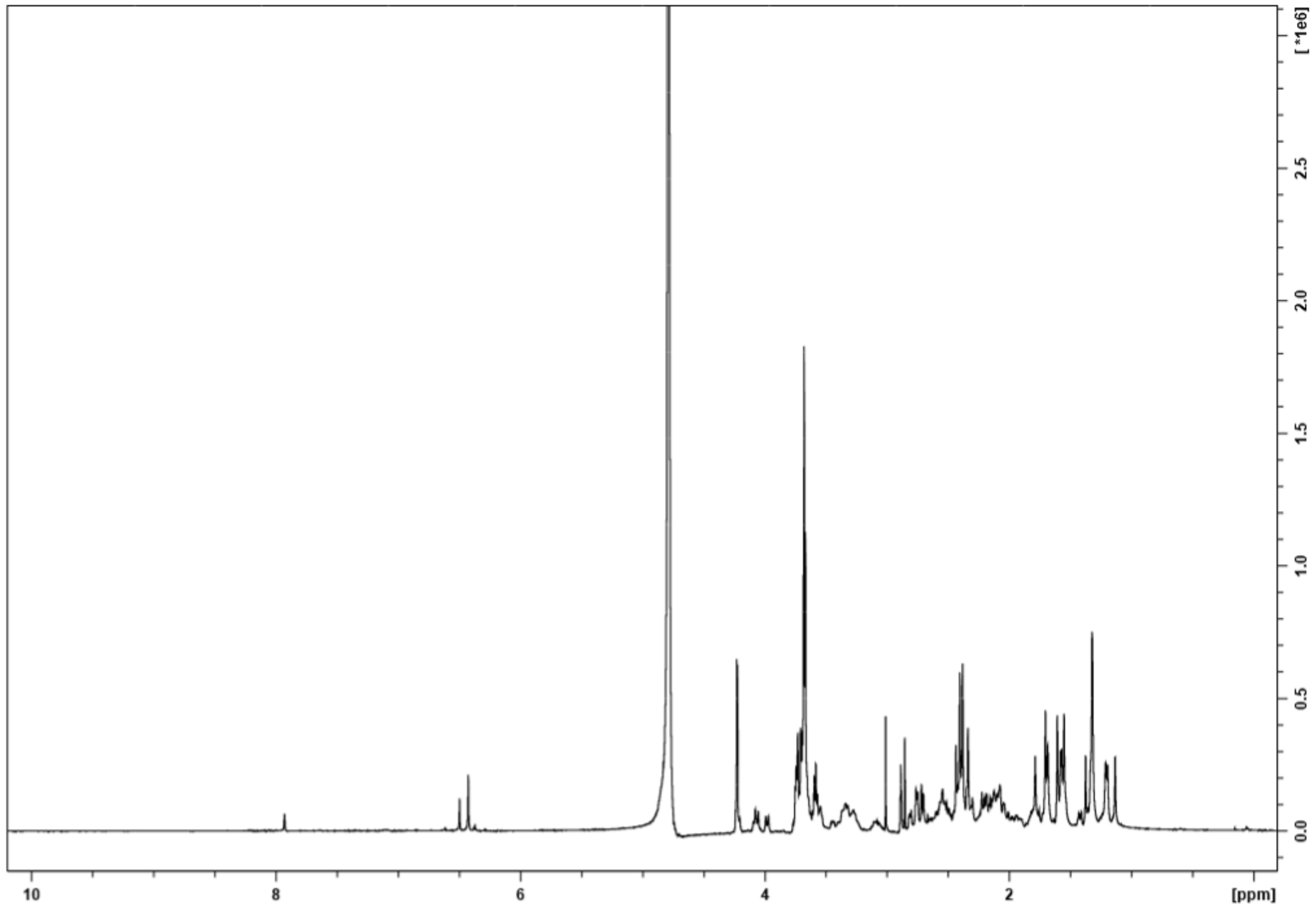

Figure S76. ^{1}H NMR of **9** (400 MHz, 298K, D_2O).

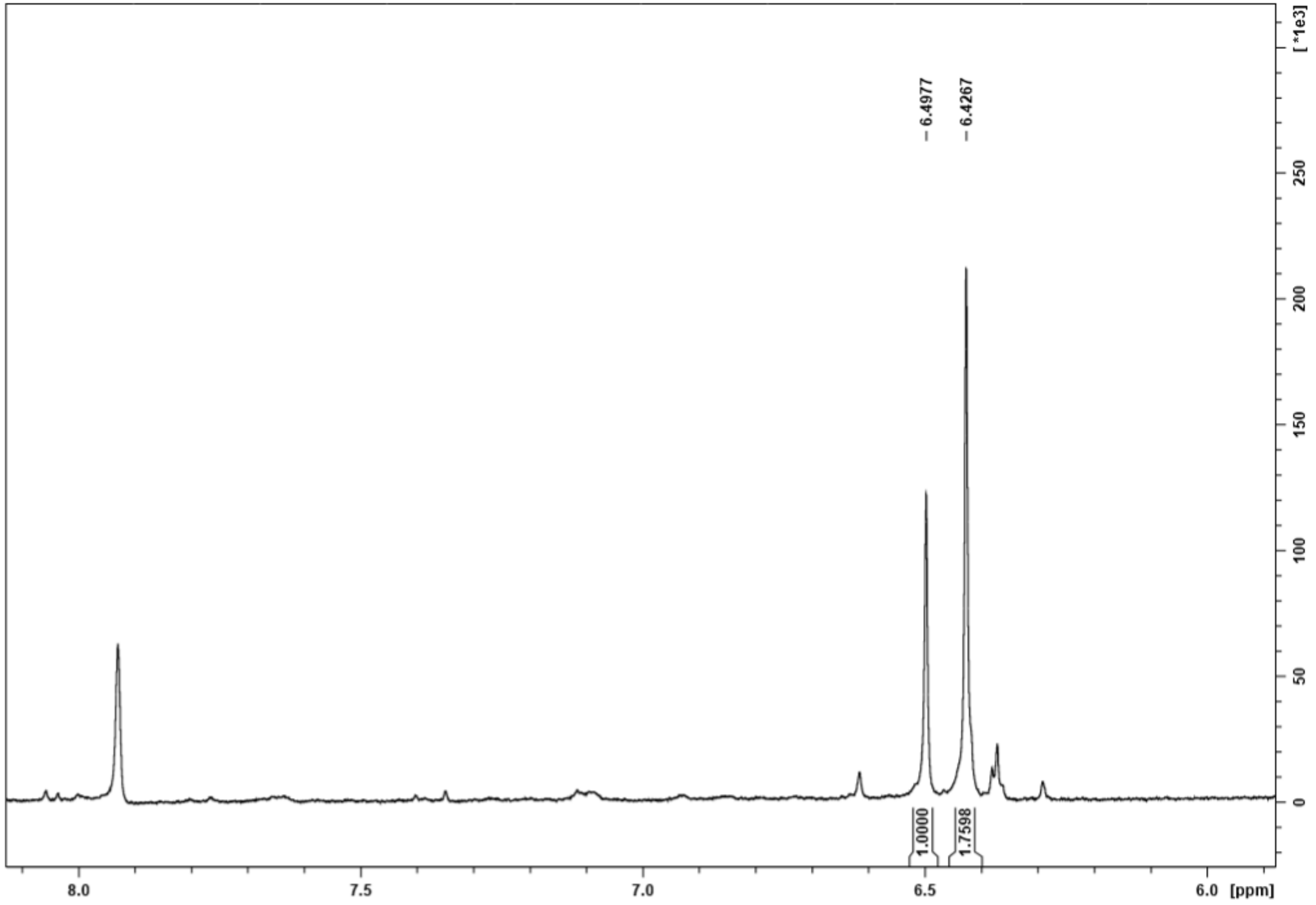

Figure S77. ^{1}H NMR of **9** (400 MHz, 298K, D_2O) (Aromatic). Characteristic signals (H19) of β- (6.50) and α- (6.43) aquo-isomers of **9** are observed.

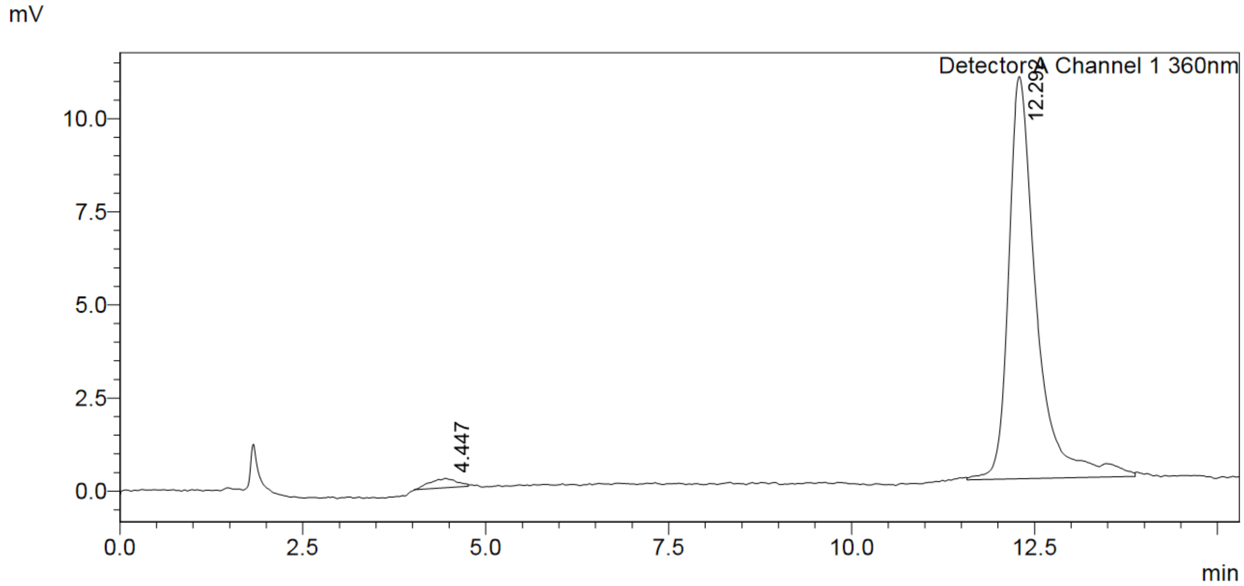

Figure S78. RP-HPLC trace showing product **17** at 12.3 min.

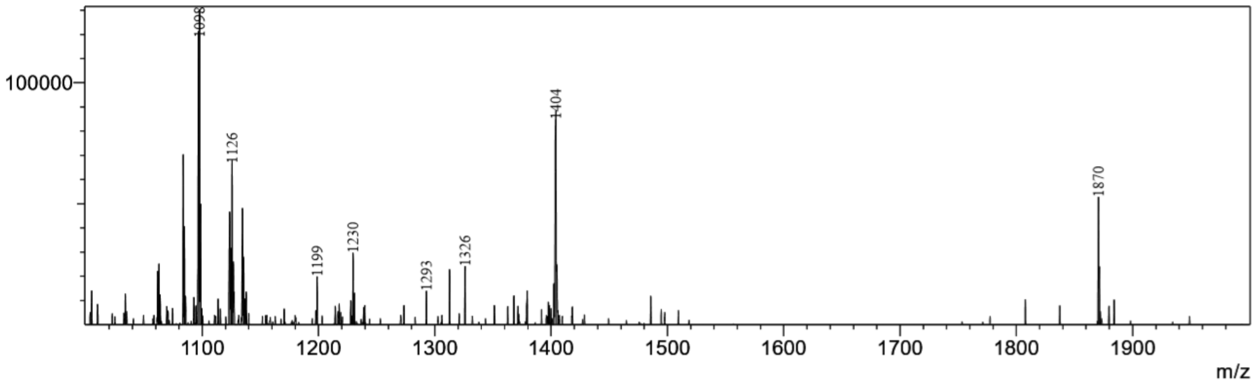

Figure S79. ESI-MS of **17**, expected m/z = 5504, observed m/z = $[M^{+}\text{-}H_2O+2H^{+}+CH_3CN]^{+3}$ 1870, $[M^{+}\text{-}H_2O+3H^{+}+CH_3OH]^{+4}$ 1404, $[M^{+}\text{-}H_2O+4H^{+}]^{+5}$ 1098 m/z.

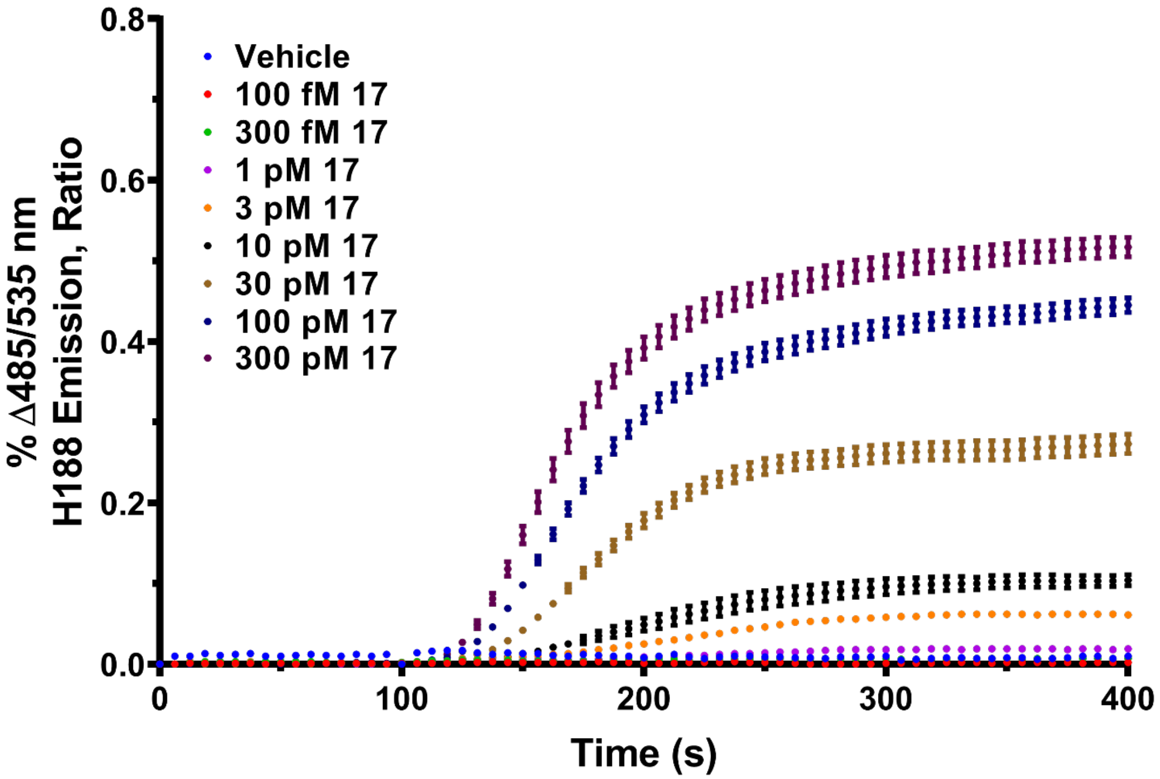

Figure S80. *In vitro* dose escalation study of **17** showing increase in cAMP levels in GLP-1R stably transfected HEK-293-H188 c20 cells.

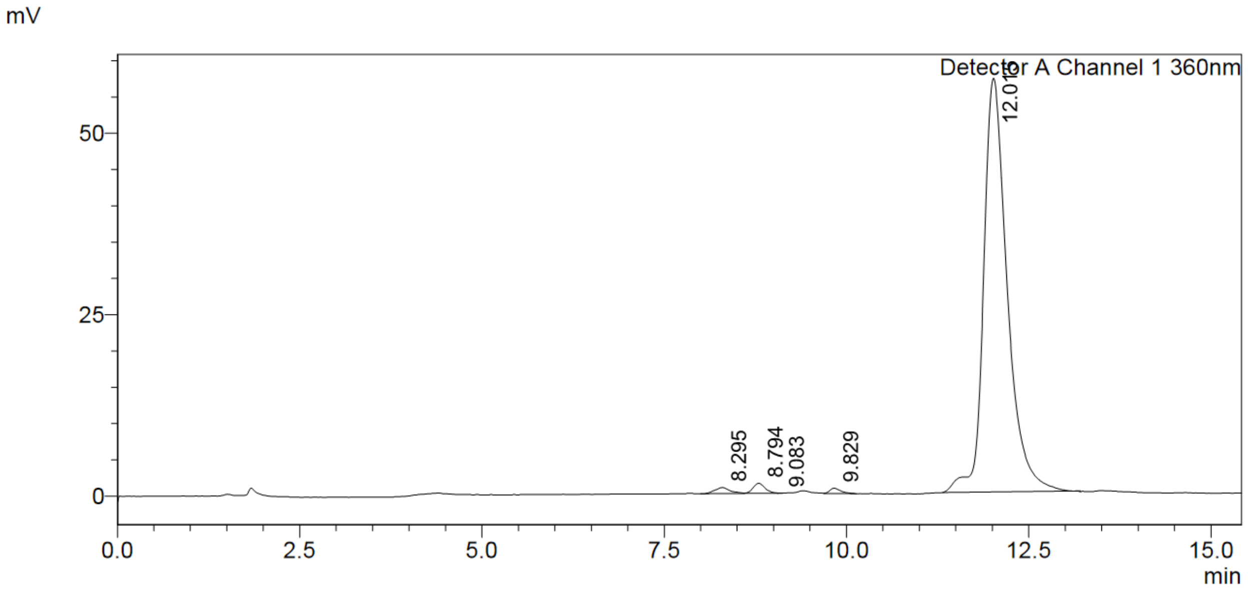

Figure S81. RP-HPLC trace showing product **25** at 12.0 min.

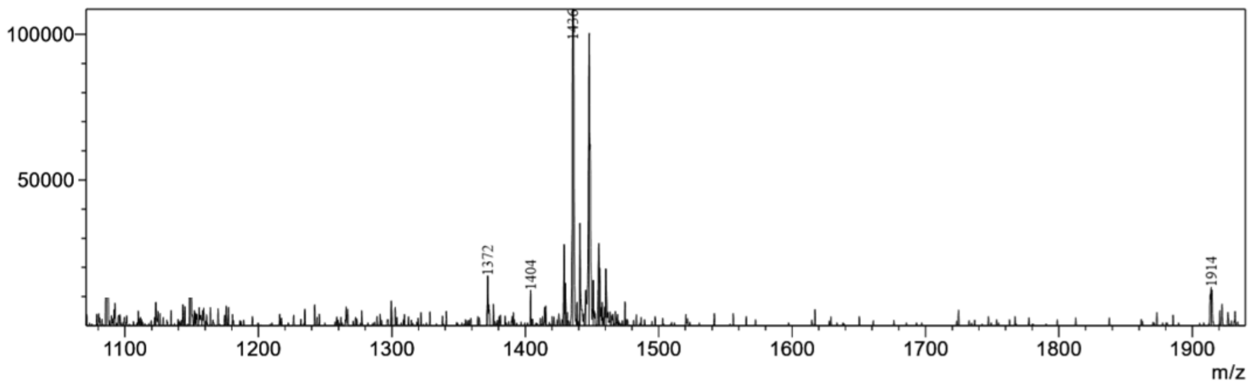

Figure S82. ESI-MS of **25**, expected m/z = 5632, observed m/z = $[M^{+}\text{-}H_2O+2H^{+}+CH_3CN]^{+3}$ 1914, $[M^{+}\text{-}H_2O+3H^{+}+CH_3OH]^{+4}$ 1436 m/z.

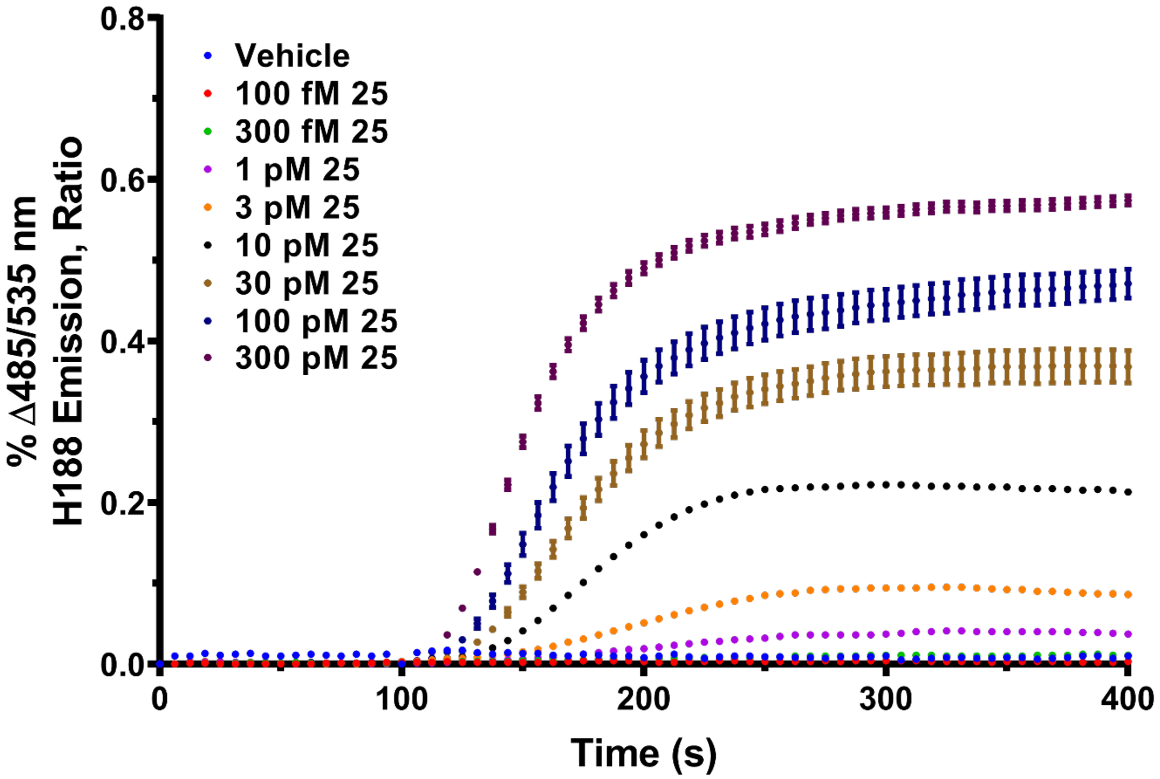

Figure S83. *In vitro* dose escalation study of **25** showing increase in cAMP levels in GLP-1R stably transfected HEK-293-H188 c20 cells.

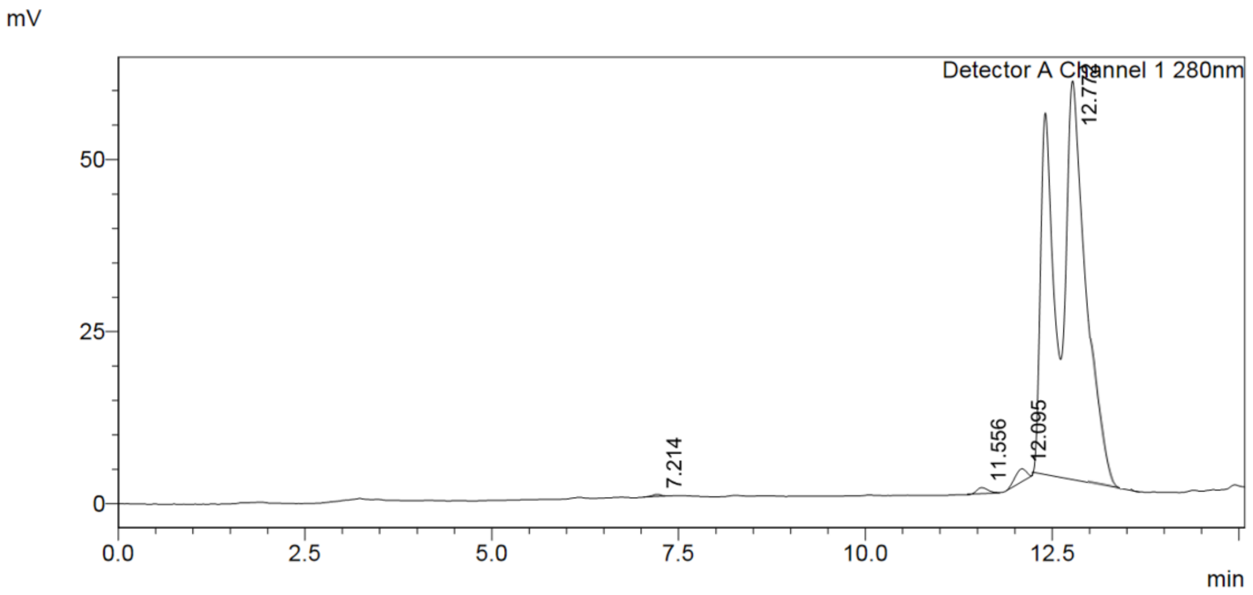

Figure S84. RP-HPLC trace showing the α- and β- isomer products of **10** at 12.4 and 12.8 min.

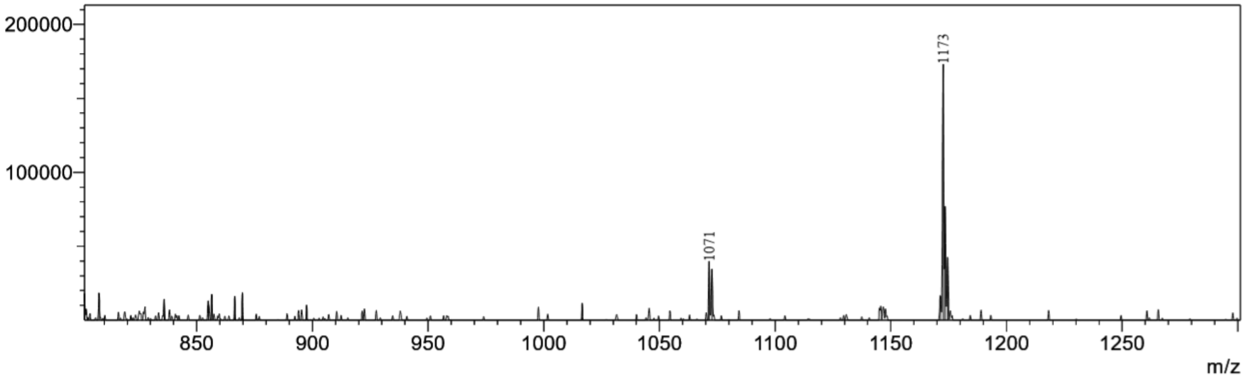

Figure S85. ESI-MS of **10**, expected m/z = 1191, observed m/z = $[M^{+}-H_2O]^{+1}$ 1173.

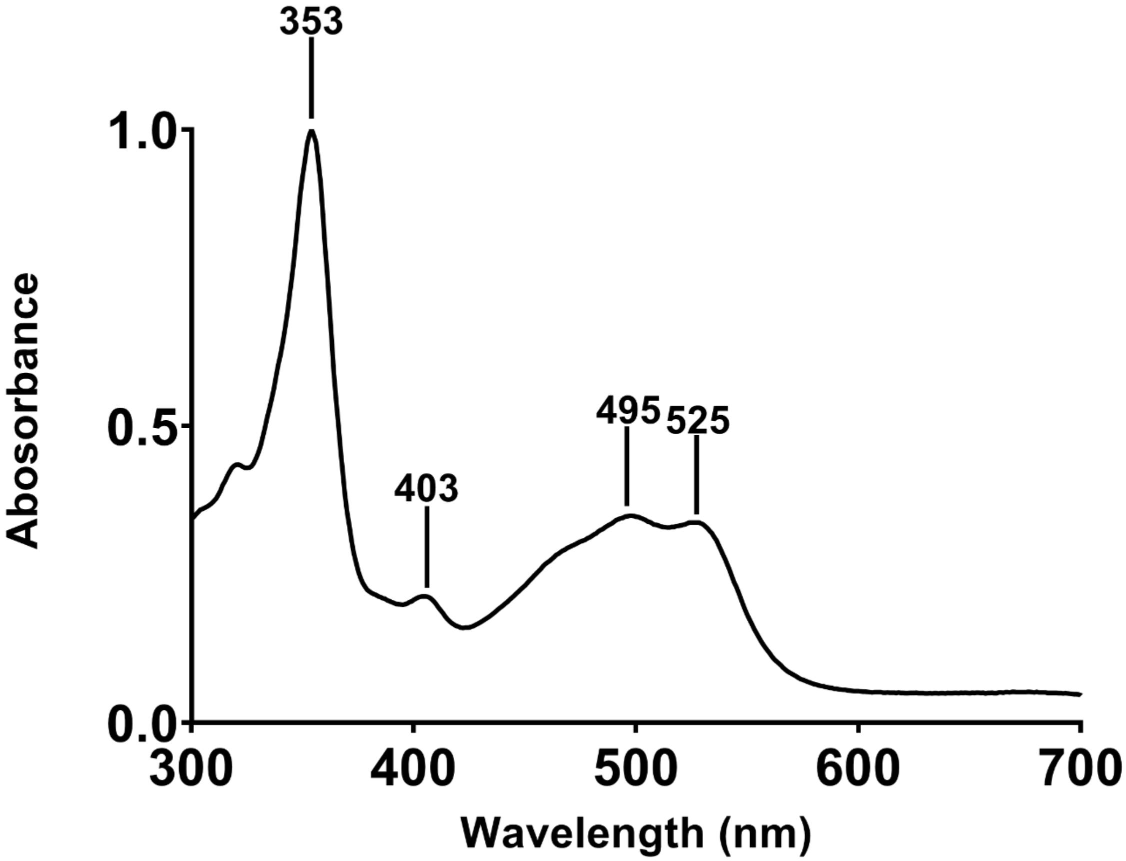

Figure S86. Electronic absorption spectra of **10** in water.

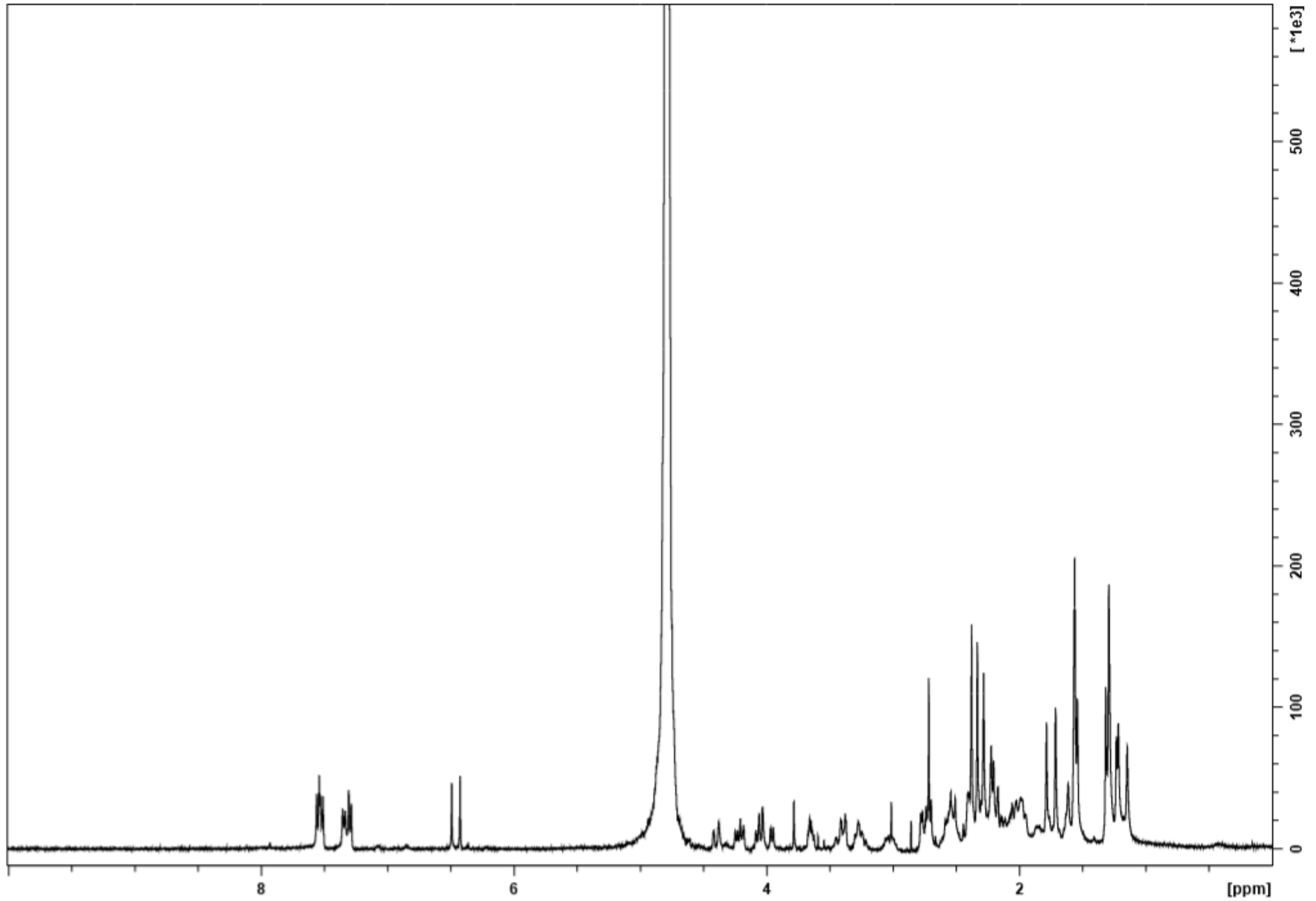

Figure S87. ^1H NMR of **10** (400 MHz, 298K, D2O).

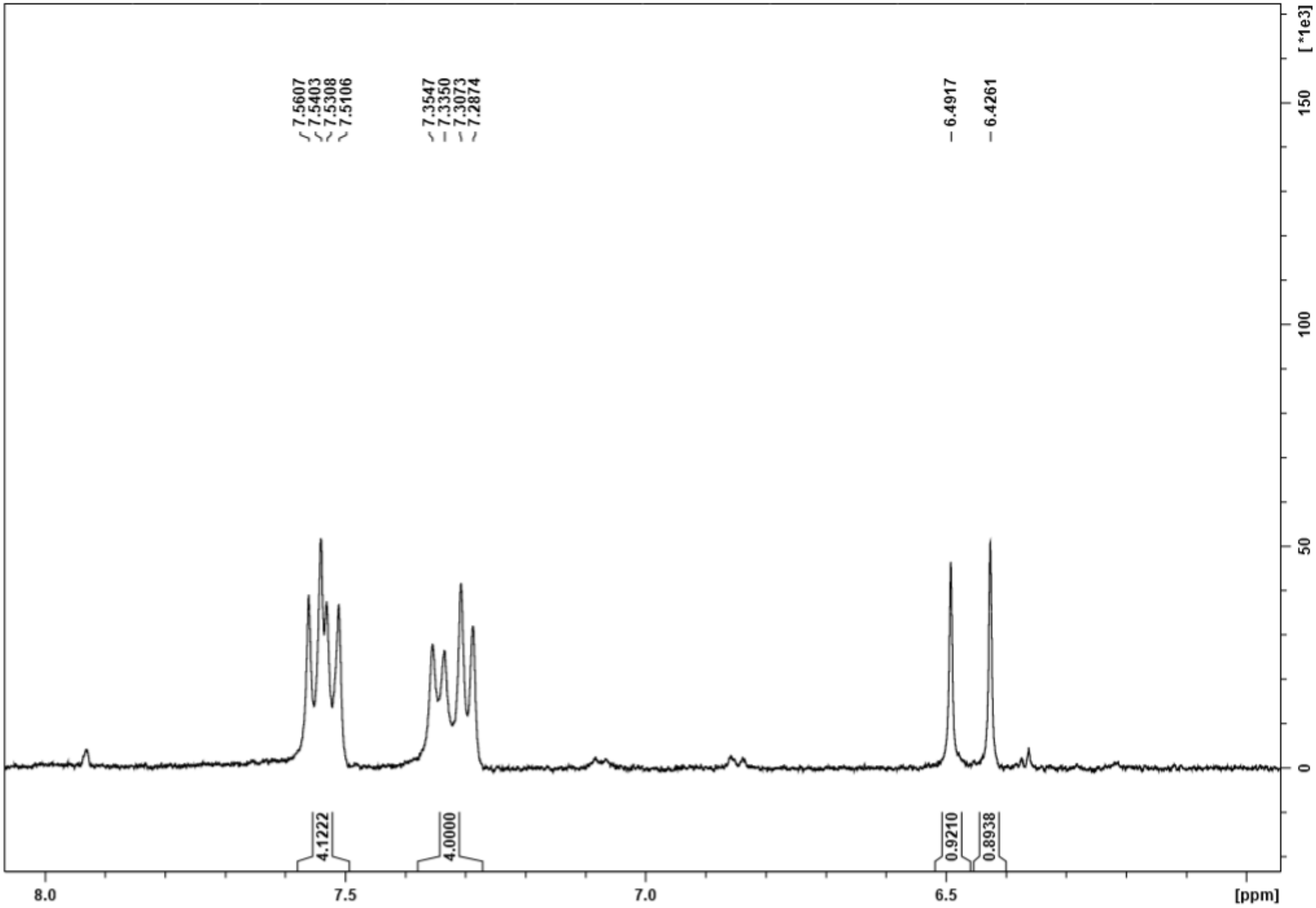

Figure S88. ^{1}H NMR of **10** (400 MHz, 298K, D2O) (Aromatic). Characteristic Signals (H19) of and β- (6.49) and α- (6.43) aquo-isomers of **10** are Identified. Additional peak groupings between 7.56-7.51 and 7.35-7.28 are indicative of the phenyl ring linker.

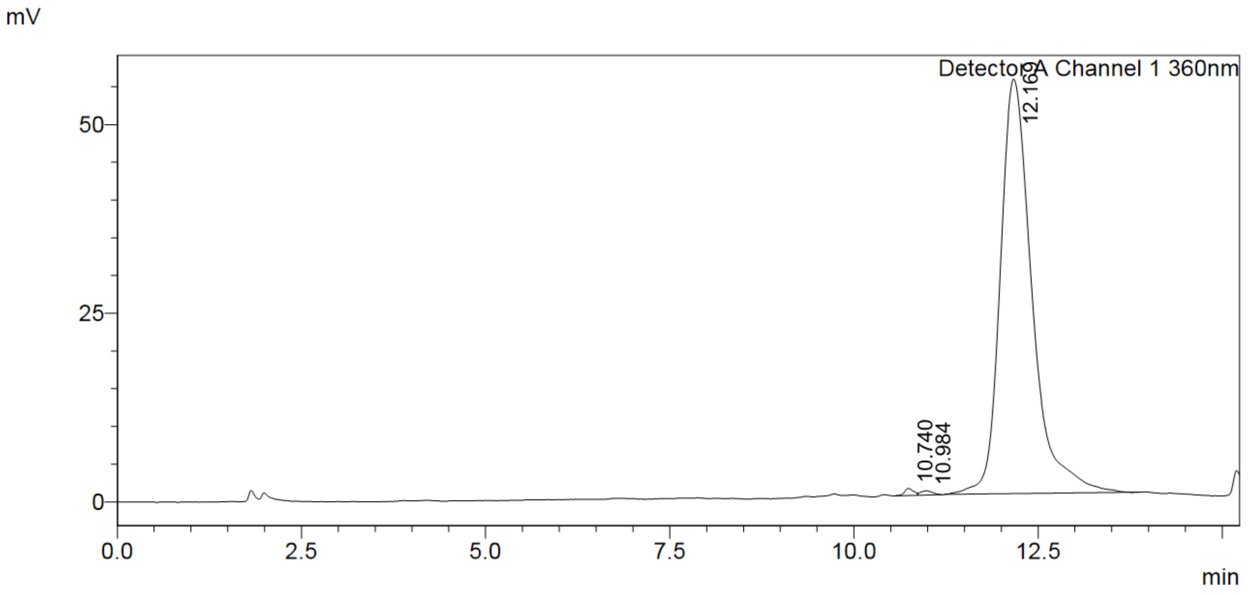

Figure S89. RP-HPLC trace showing product **18** at 12.2 min.

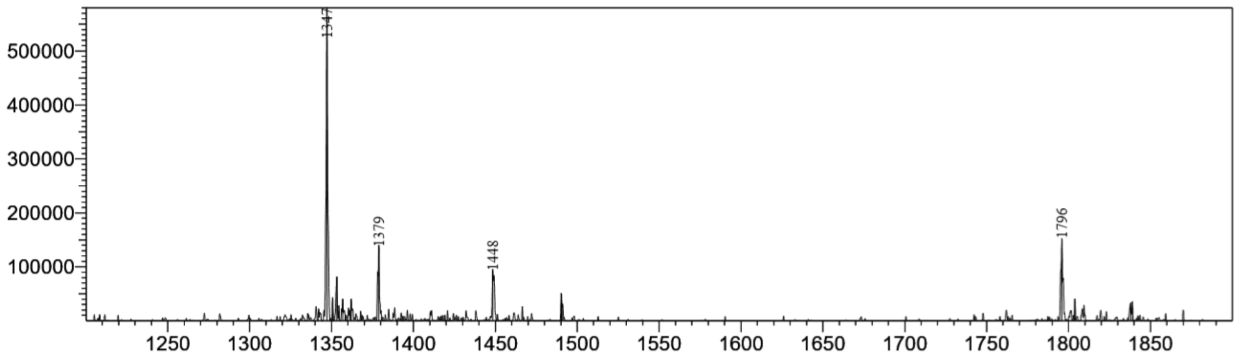

Figure S90. ESI-MS of **18**, expected m/z = 5403, observed m/z = $[M^{+}\text{-}H_2O+2H^{+}]^{+3}$ 1796, $[M^{+}\text{-}H_2O+CH_3OH+3H^{+}]^{+4}$ 1379, $[M^{+}\text{-}H_2O+3H^{+}]^{+4}$ 1347 m/z.

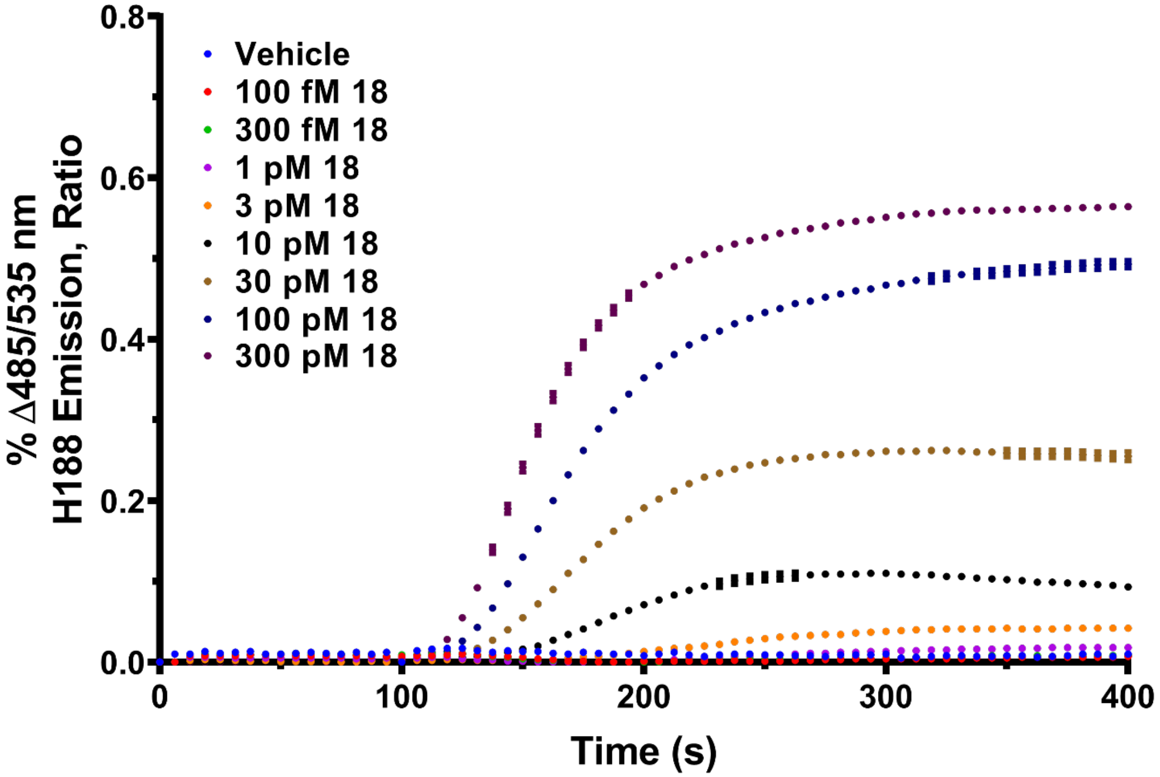

Figure S91. *In vitro* dose escalation study of **18** showing increase in cAMP levels in GLP-1R stably transfected HEK-293-H188 c20 cells.

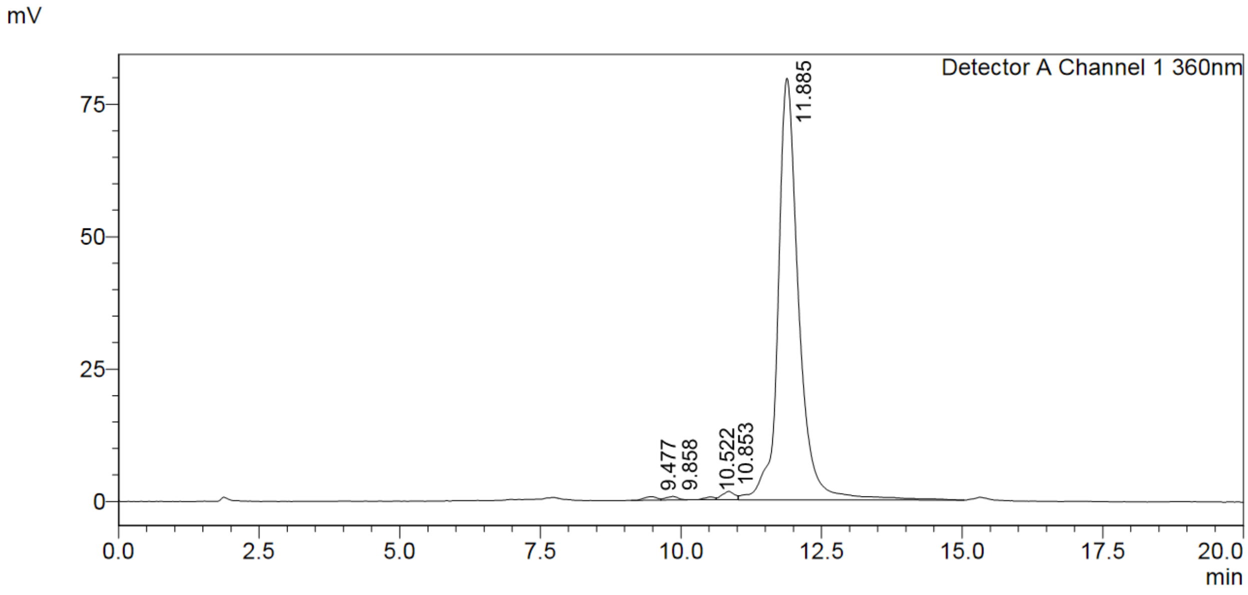

Figure S92. RP-HPLC trace showing product **26** at 11.9 min.

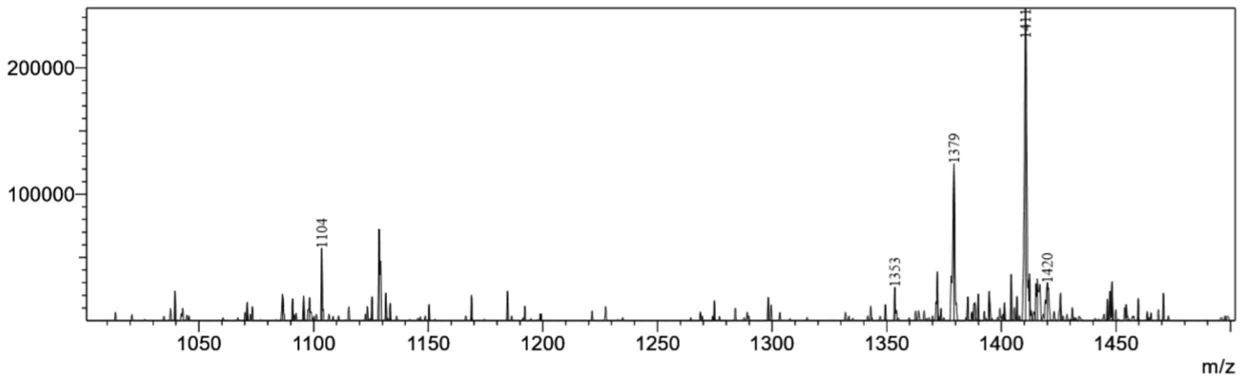

Figure S93. ESI-MS of **26**, expected m/z = 5531, observed m/z = $[M^+-H_2O+CH_3OH+3H^+]^{+4}$ 1411, $[M^+-H_2O+3H^+]^{+4}$ 1379, , $[M-H_2O+4H^+-CN]^{+4}$ 1353, $[M^+-H_2O+4H^+]^{+5}$ 1104 m/z.

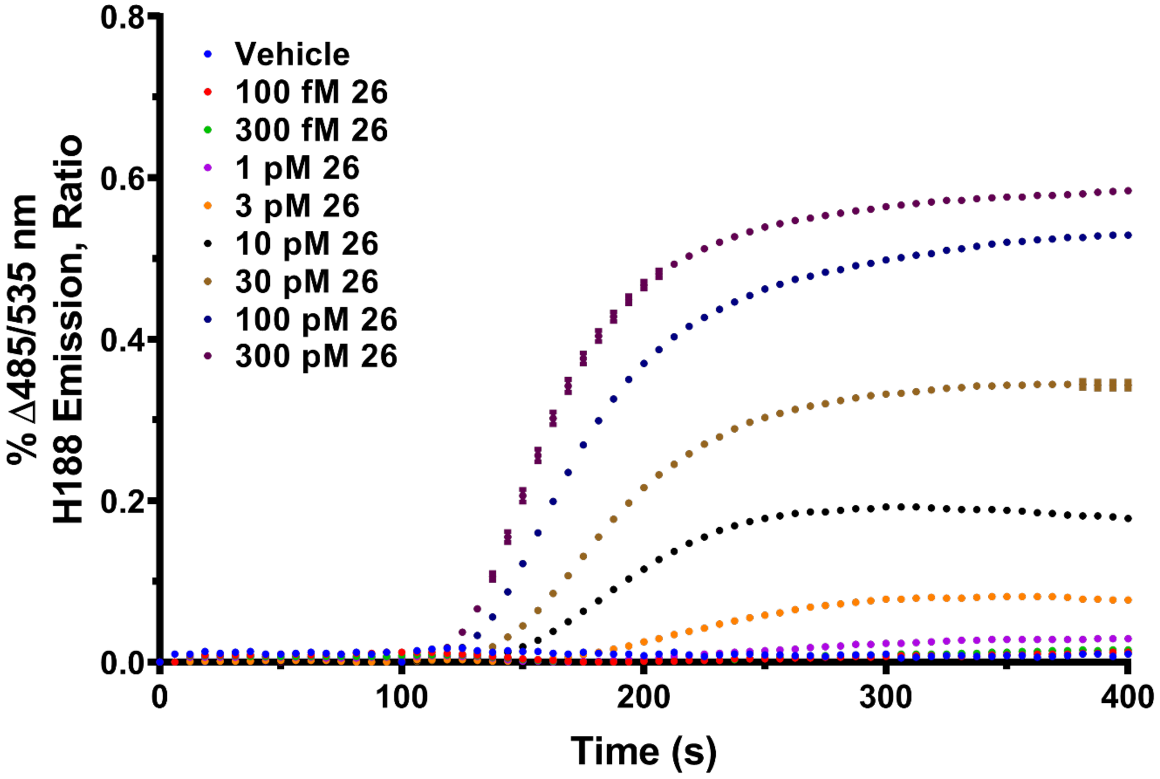

Figure S94. *In vitro* dose escalation study of **26** showing increase in cAMP levels in GLP-1R stably transfected HEK-293-H188 c20 cells.

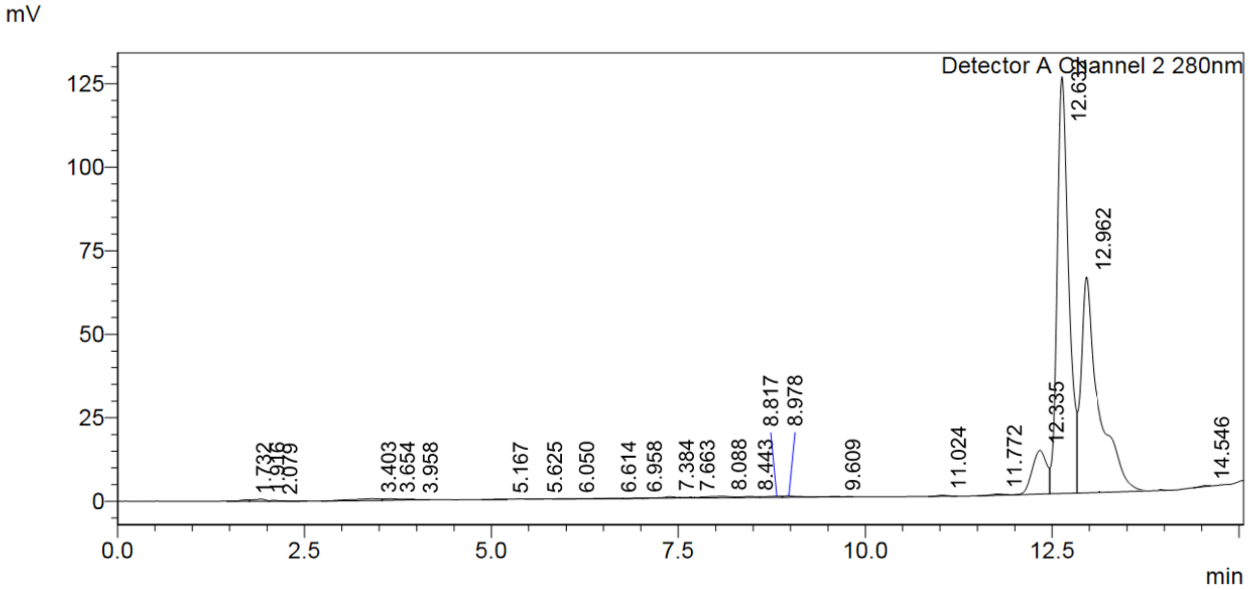

Figure S95. RP-HPLC trace showing the α- and β- isomer products of **11** at 12.6 and 13.0 min.

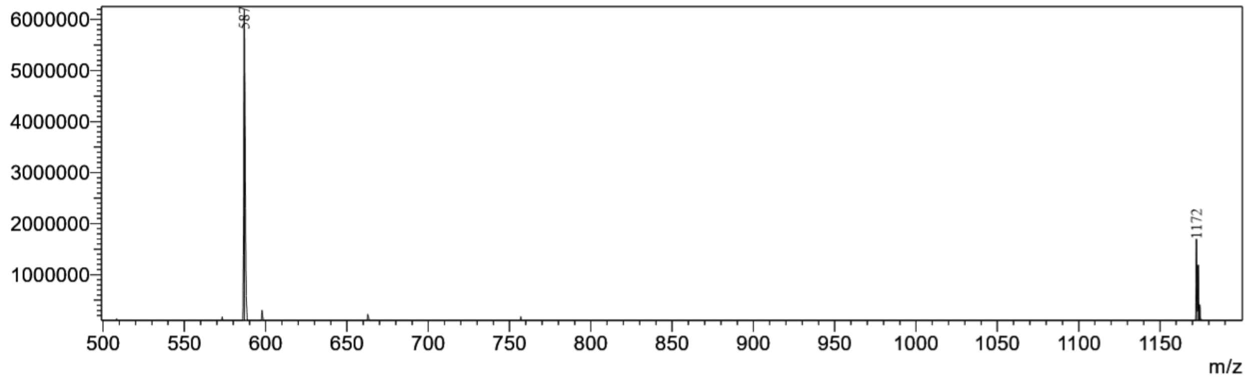

Figure S96. ESI-MS of **11**, expected m/z = 1191, observed m/z = $[M^{+}-H_2O]^{+1}$ 1172, $[M^{+}-H_2O+H^{+}]^{+2}$ 587 m/z.

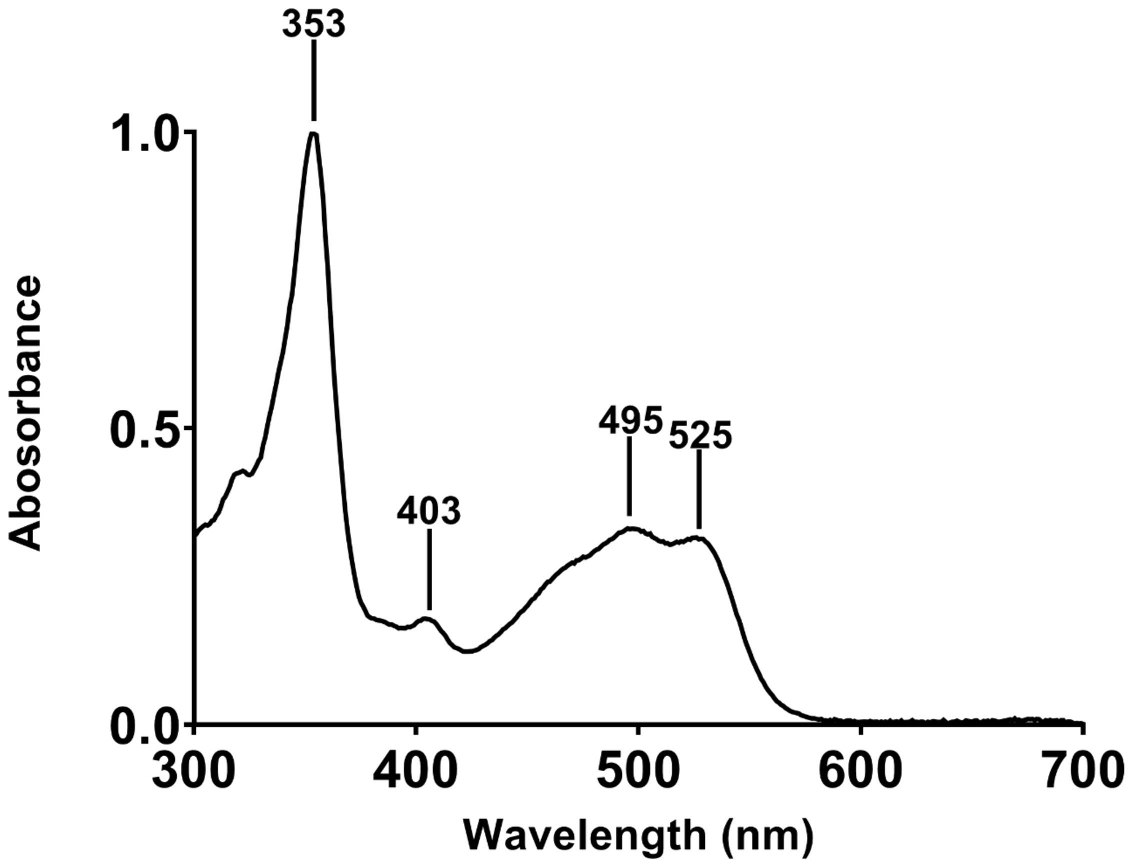

Figure S97. Electronic absorption spectra of **11** in water.

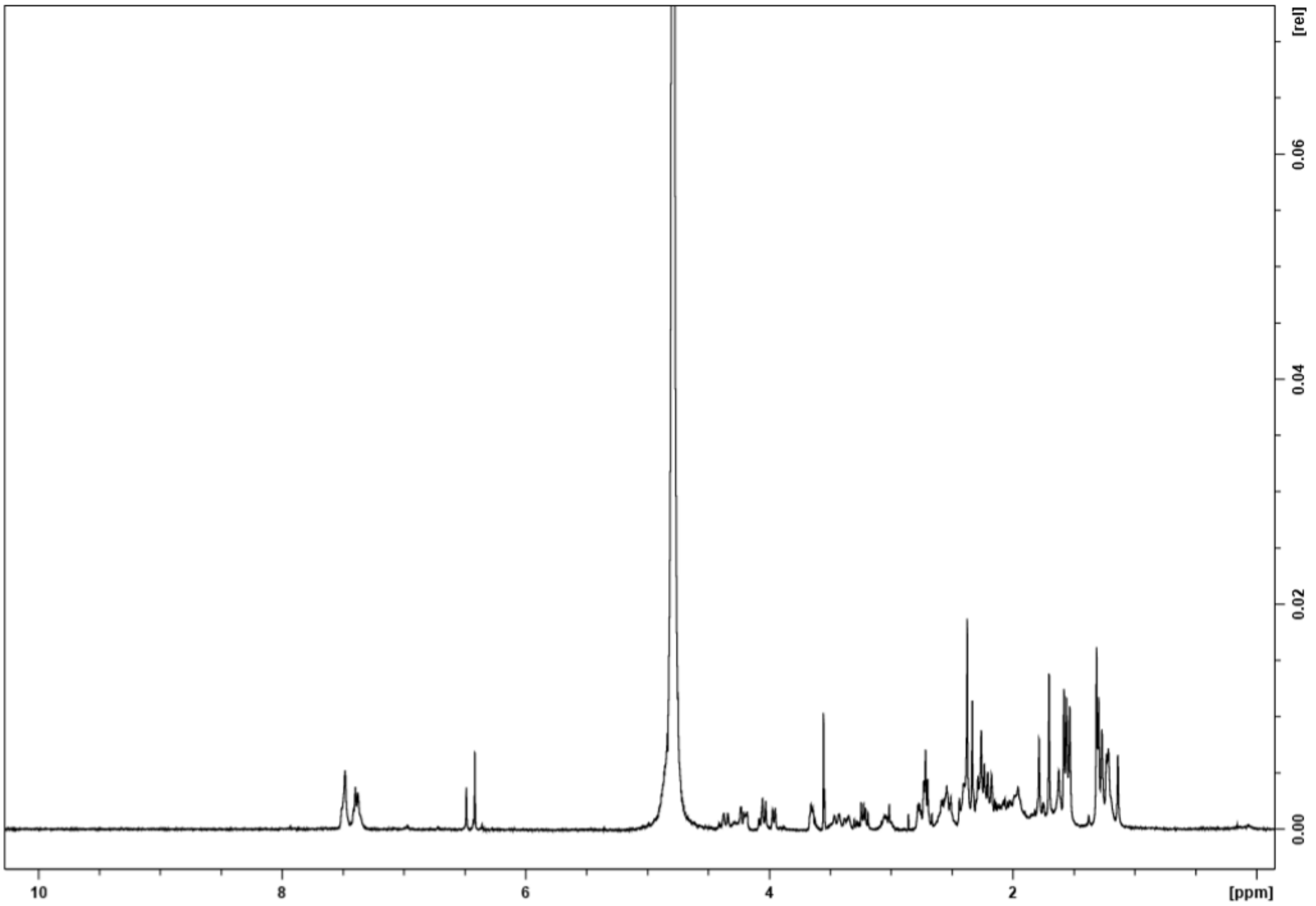

Figure S98. ^{1}H NMR of **11**. (400 MHz, 298K, D_2O).

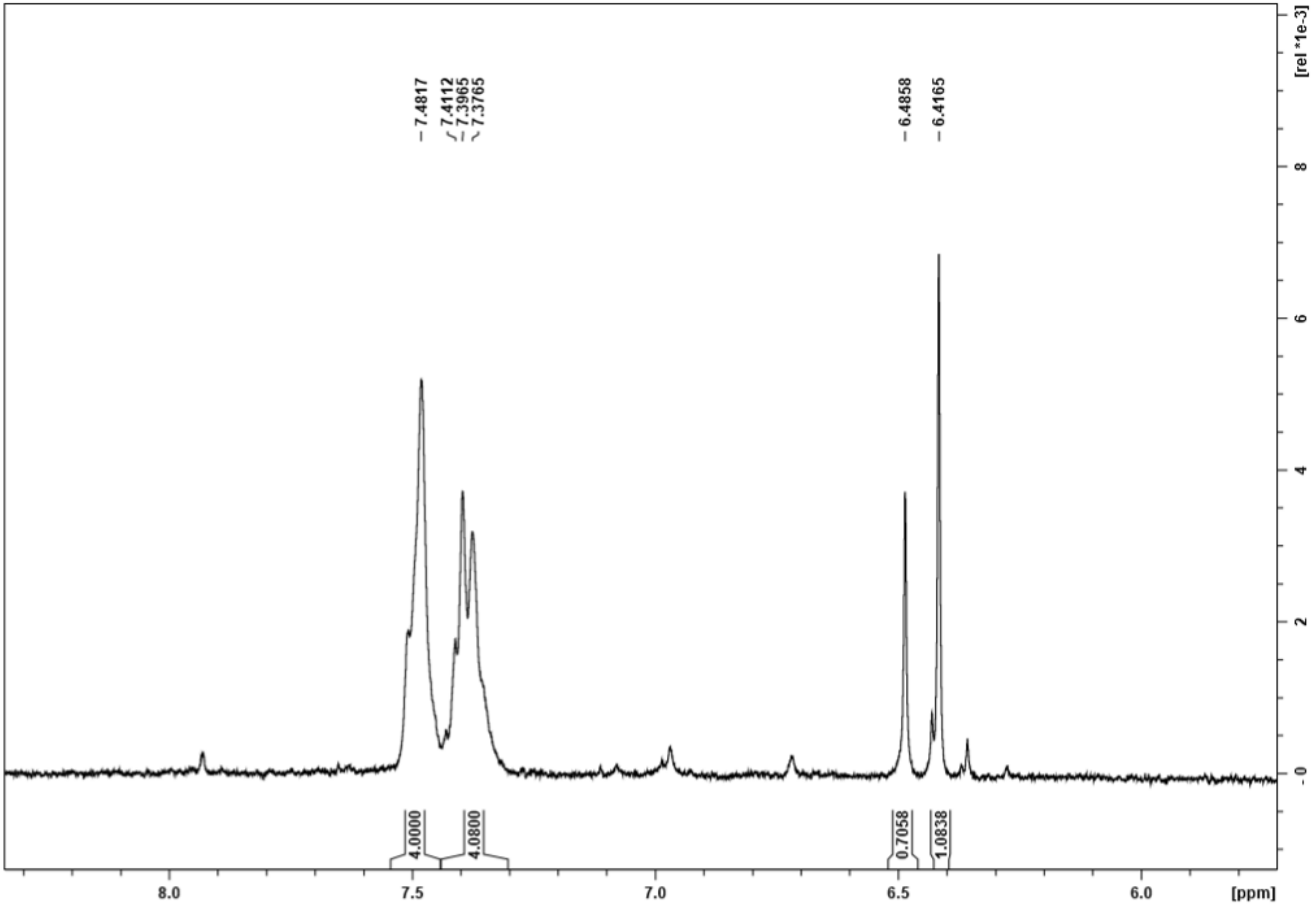

Figure S99. ^{1}H NMR of **11**. (400 MHz, 298K, D_2O) (Aromatic). Characteristic signals (H19) of β- (6.49) and α- (6.42) aquo-isomers of **11** are observed. Additional peak groupings between 7.48-7.37 are indicative of the phenyl ring linker.

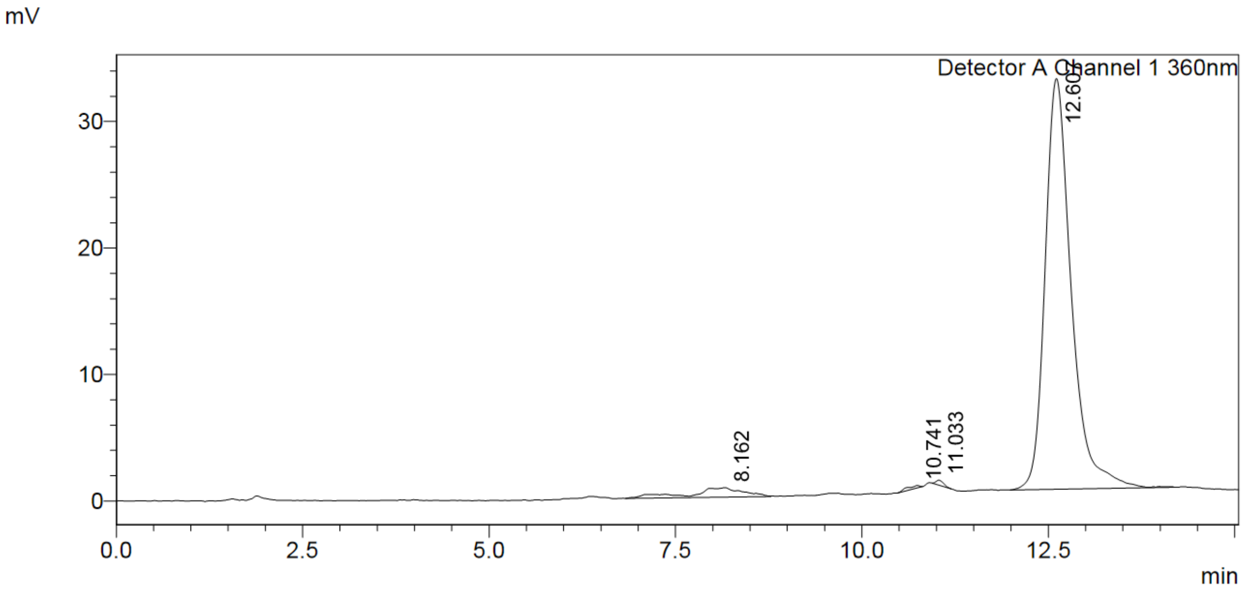

Figure S100. RP-HPLC trace showing product **19** at 12.6 min.

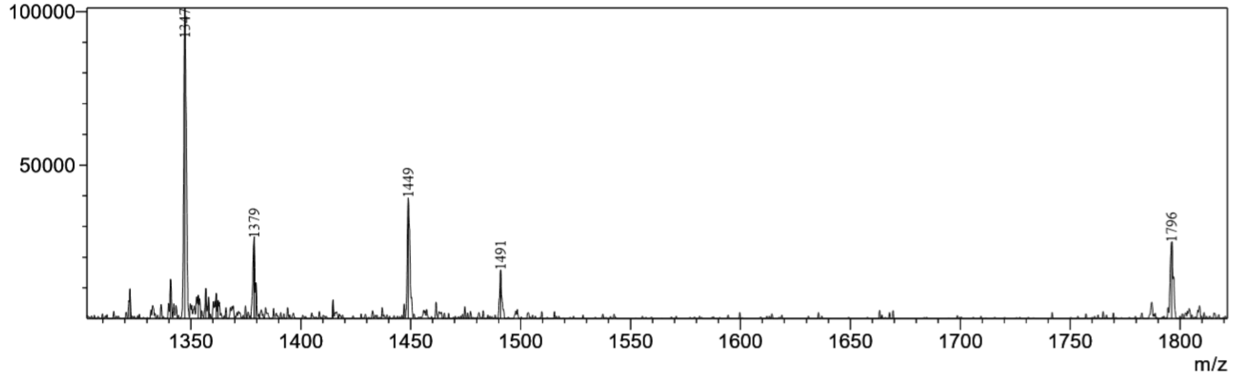

Figure S101. ESI-MS of **19**, expected m/z = 5403, observed m/z = $[M^{+}\text{-}H_2O+2H^{+}]^{+3}$ 1796, $[M^{+}\text{-}H_2O+CH_3OH+3H^{+}]^{+4}$ 1379, $[M^{+}\text{-}H_2O+3H^{+}]^{+4}$ 1347 m/z.

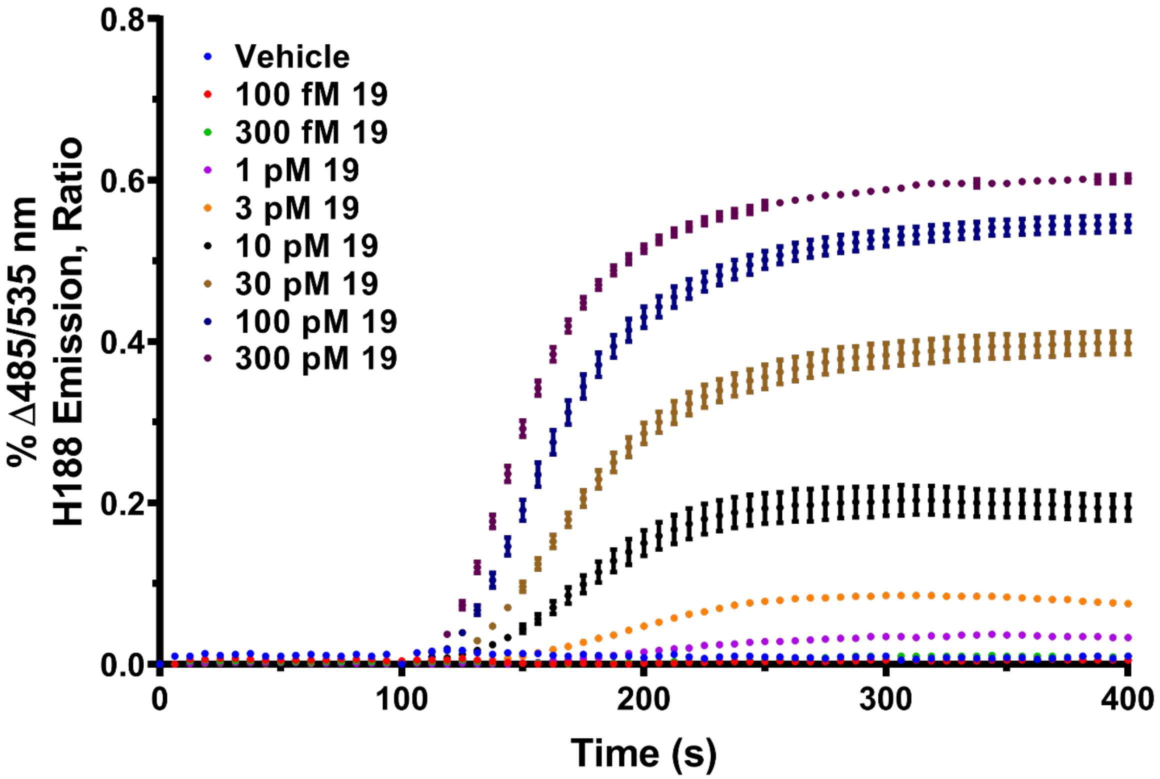

Figure S102. *In vitro* dose escalation study of **19** showing increase in cAMP levels in GLP-1R stably transfected HEK-293-H188 c20 cells.

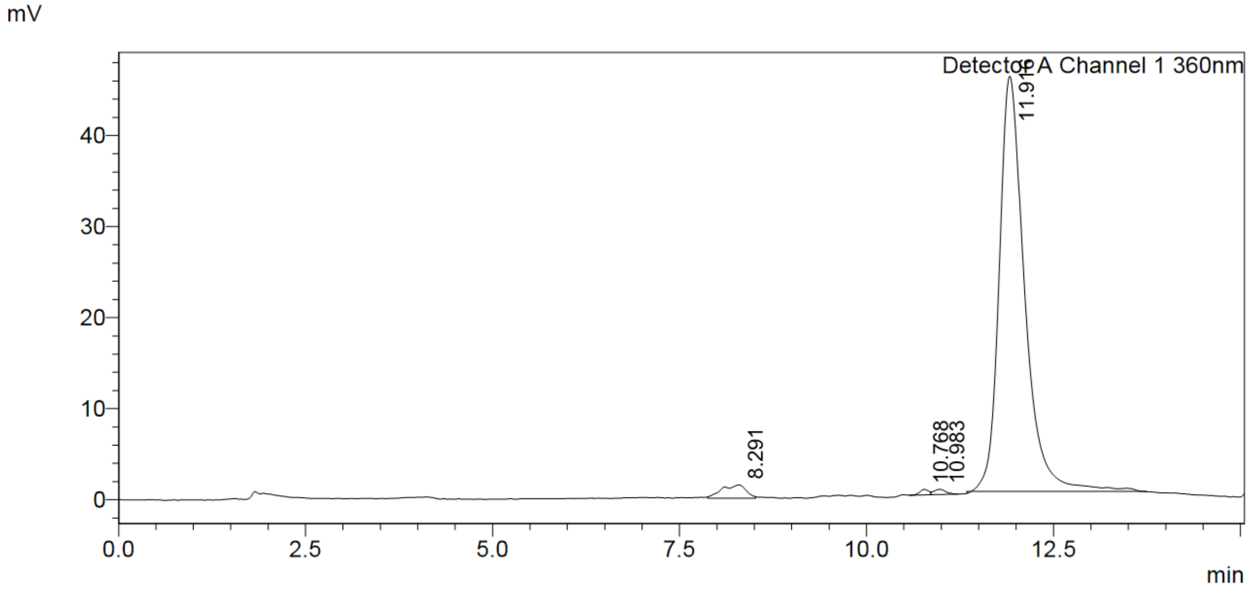

Figure S103. RP-HPLC trace showing product **27** at 11.9 min.

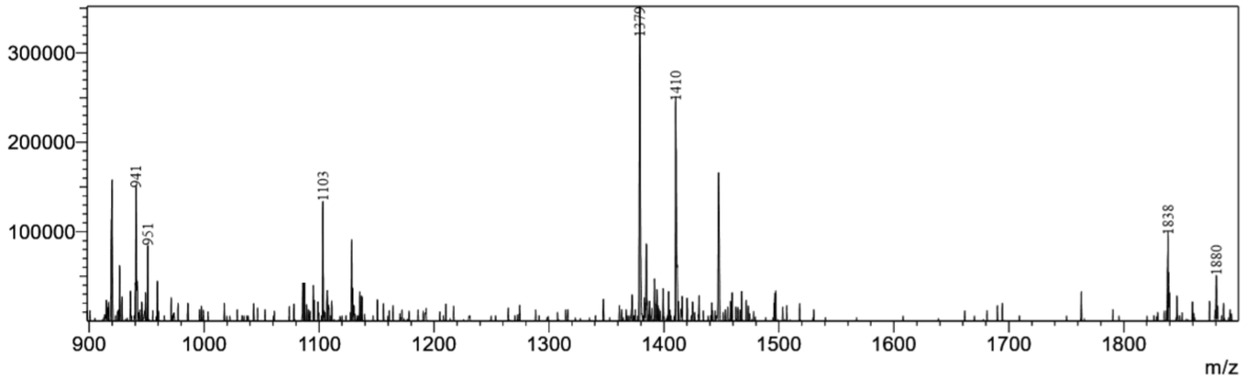

Figure S104. ESI-MS of **27**, expected m/z = 5531, observed m/z = $[M^+ - H_2O + CH_3CN + 2H^+]^{+3}$ 1880, $[M^+ - H_2O + 2H^+]^{+3}$ 1838, $[M^+ - H_2O + CH_3OH + 3H^+]^{+4}$ 1410, $[M^+ - H_2O + 3H^+]^{+4}$ 1379, $[M^+ - H_2O + 4H^+]^{+5}$ 1103, $[M^+ - H_2O + 5H^+]^{+6}$ 919 m/z.

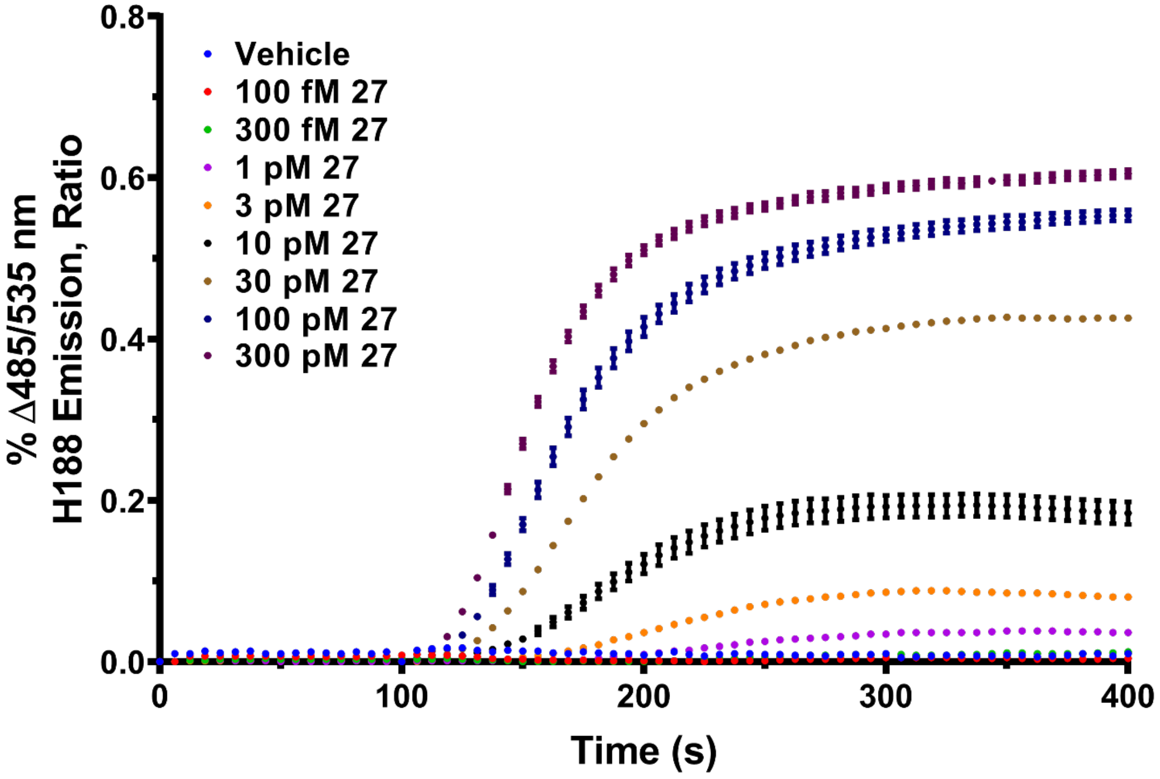

Figure S105. *In vitro* dose escalation study of **27** showing increase in cAMP levels in GLP-1R stably transfected HEK-293-H188 c20 cells.

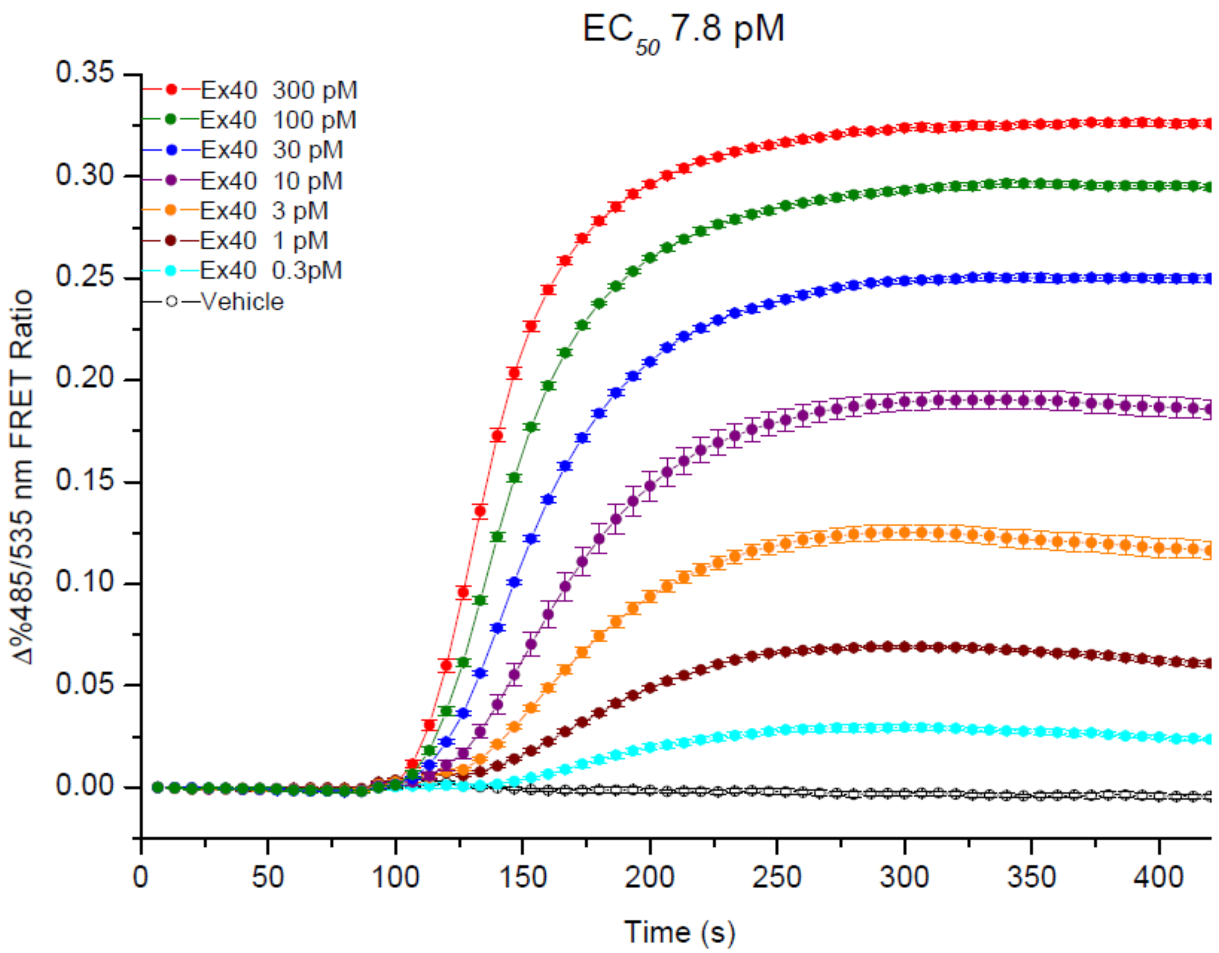

Figure 106. *In vitro* dose escalation study of Ex40 showing increase in cAMP levels in rGLP-1R transiently transfected HEK-293-H188 c24 cells.

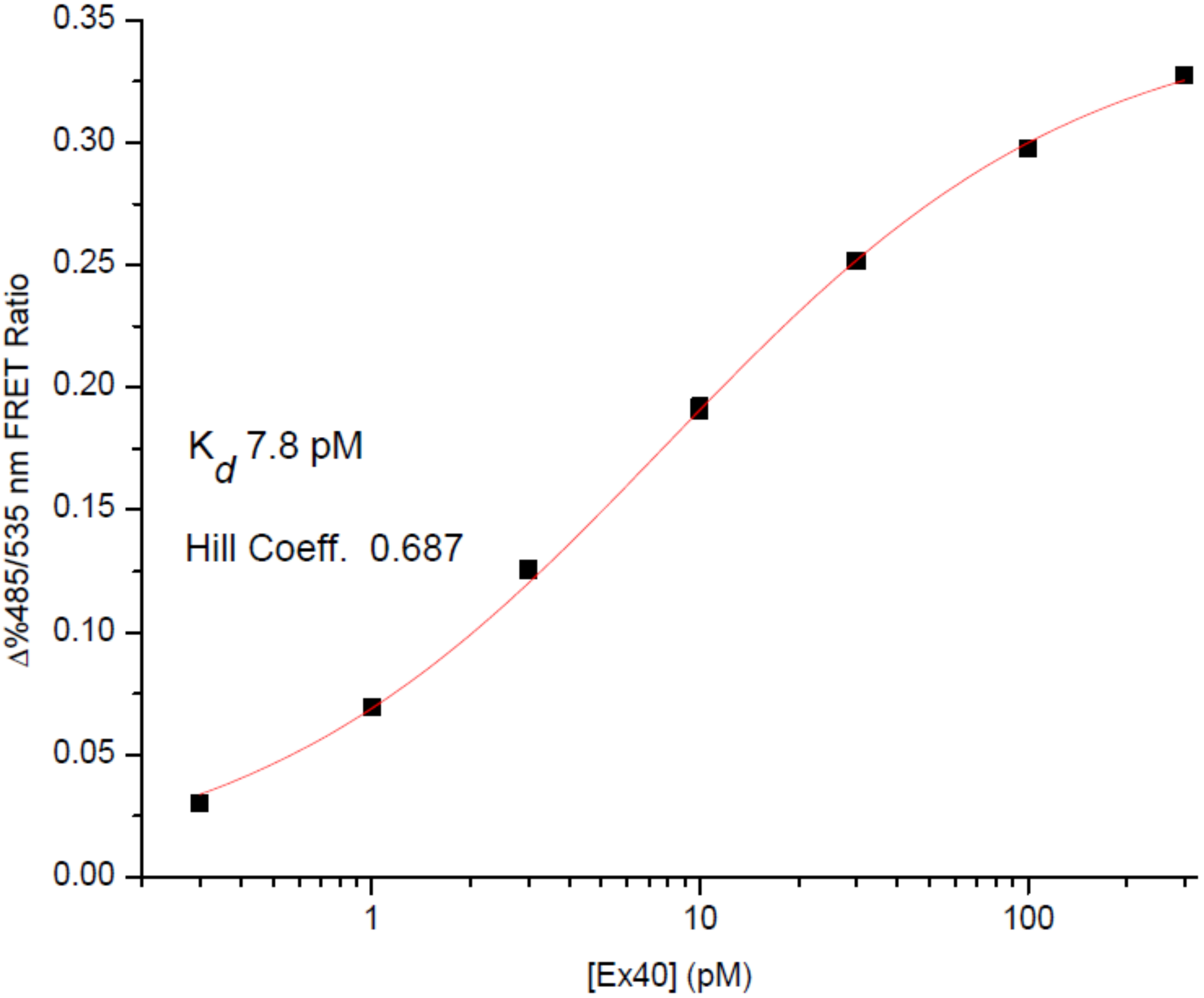

Figure 107. Non-linear regression dose response analysis with hill slope of Ex40.

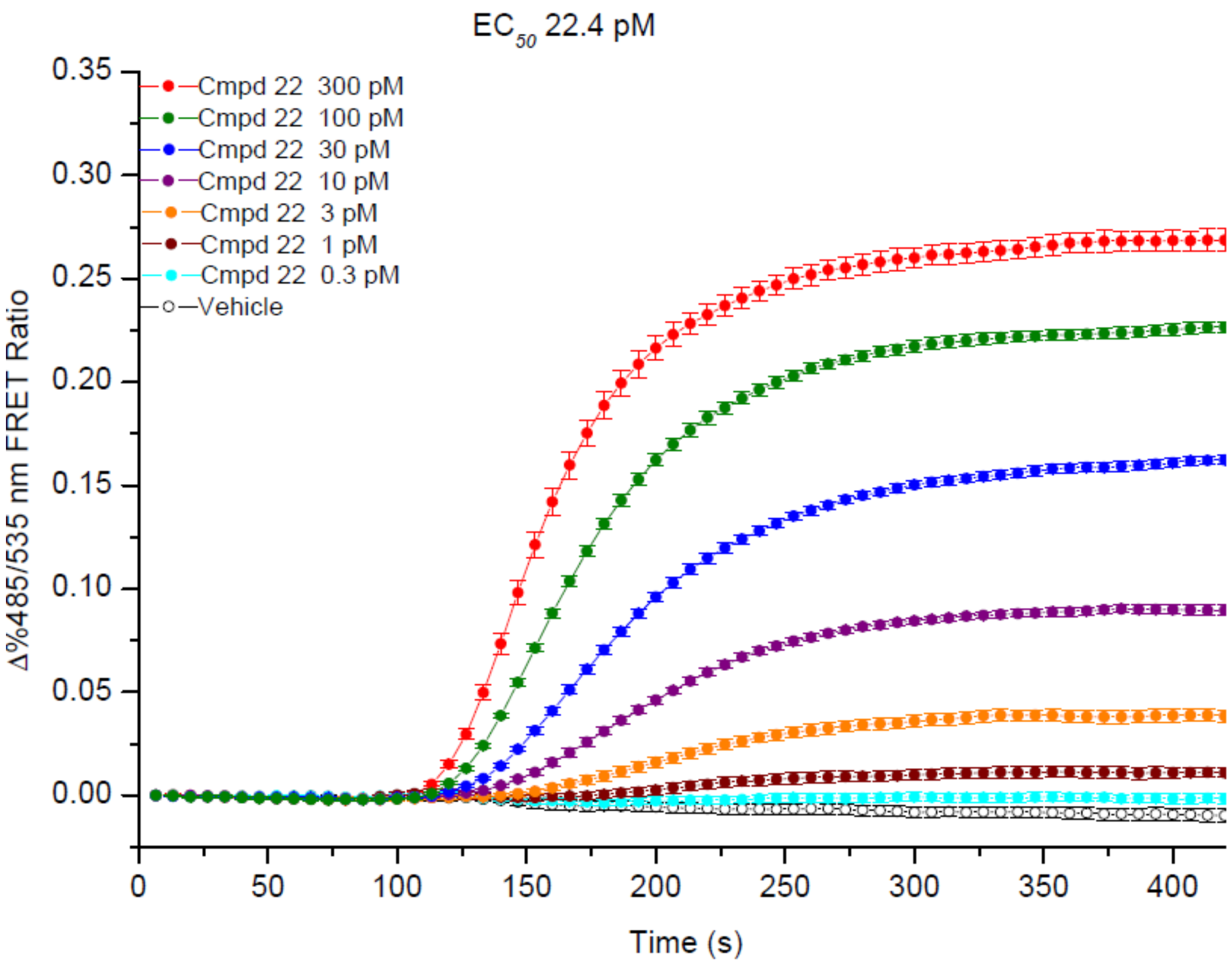

Figure 108. *In vitro* dose escalation study of **22** showing increase in cAMP levels in rGLP-1R transiently transfected HEK-293-H188 c24 cells.

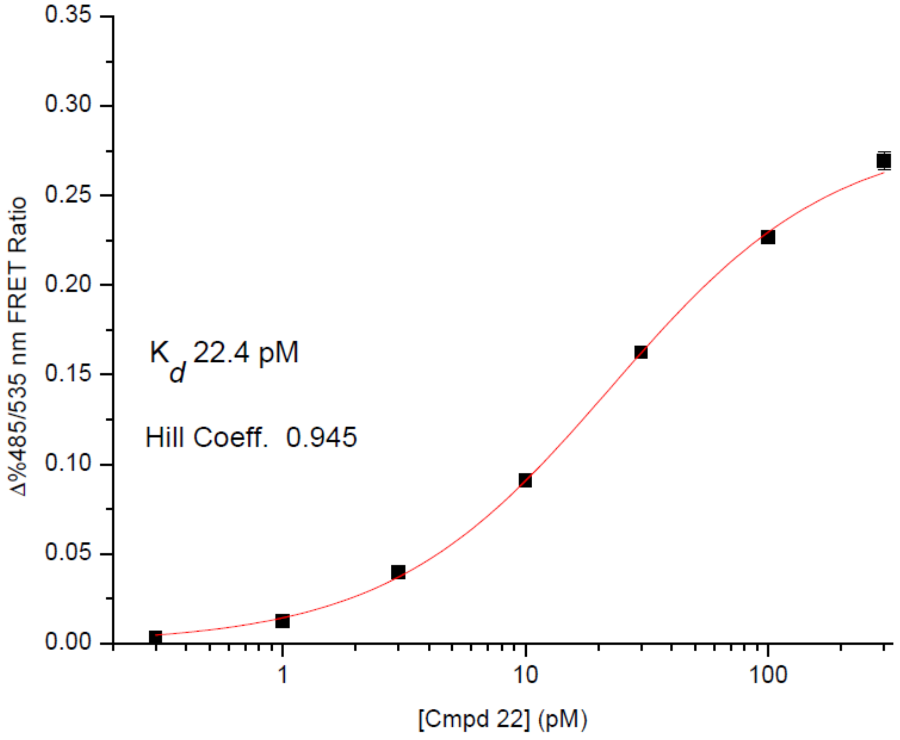

Figure 109. Non-linear regression dose response analysis with hill slope of **22**.

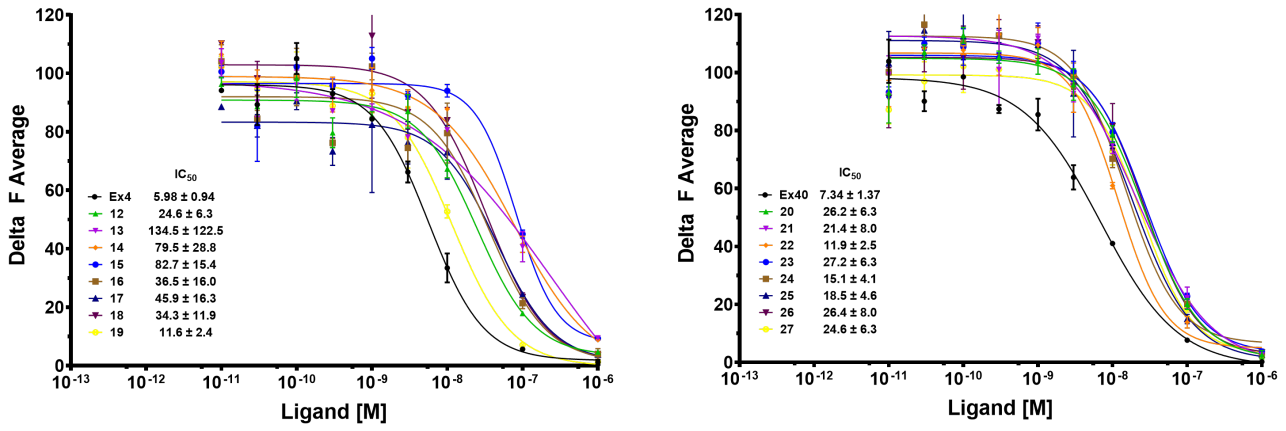

Figure S110. *In vitro* dose escalation competition binding studies of **12**-**27** compared with Ex4 and Ex40 controls against fluorescent GLP-1red.

1. Zhou, K.; Zelder, F. Identification of diastereomeric cyano-aqua cobinamides with a backbone-modified vitamin B12 derivative and with ^{1}H NMR spectroscopy. *Eur. J. Inorg. Chem*. **2011**, 53-57.
